# Freezing of gait in Parkinson's disease is related to imbalanced stopping–related cortical activity

**DOI:** 10.1093/braincomms/fcae259

**Published:** 2024-08-02

**Authors:** Helena M Cockx, Robert Oostenveld, Yuli A Flórez R, Bastiaan R Bloem, Ian G M Cameron, Richard J A van Wezel

**Affiliations:** Department of Neurobiology, Faculty of Science, Donders Institute for Brain, Cognition and Behaviour, Radboud University, 6525AJ Nijmegen, The Netherlands; Department of Neurology, Center of Expertise for Parkinson and Movement Disorders, Donders Institute for Brain, Cognition and Behaviour, Radboud University Medical Center, 6525GC Nijmegen, The Netherlands; Donders Center for Cognitive Neuroimaging, Donders Institute for Brain, Cognition and Behaviour, Radboud University, 6525EN Nijmegen, The Netherlands; NatMEG, Karolinska Institutet, 17177 Stockholm, Sweden; Department of Neurobiology, Faculty of Science, Donders Institute for Brain, Cognition and Behaviour, Radboud University, 6525AJ Nijmegen, The Netherlands; Department of Psychiatry, Maastricht University Medical Center, 6229HX Maastricht, The Netherlands; Department of Neurology, Center of Expertise for Parkinson and Movement Disorders, Donders Institute for Brain, Cognition and Behaviour, Radboud University Medical Center, 6525GC Nijmegen, The Netherlands; Department of Neurobiology, Faculty of Science, Donders Institute for Brain, Cognition and Behaviour, Radboud University, 6525AJ Nijmegen, The Netherlands; Biomedical Signals and Systems Group, Faculty of Electrical Engineering, Mathematics and Computer Science (EEMCS), University of Twente, 7522NB Enschede, The Netherlands; Domain Expert Precision Health, Nutrition & Behavior, OnePlanet Research Center, 6525EC Nijmegen, The Netherlands; Department of Neurobiology, Faculty of Science, Donders Institute for Brain, Cognition and Behaviour, Radboud University, 6525AJ Nijmegen, The Netherlands; Biomedical Signals and Systems Group, Faculty of Electrical Engineering, Mathematics and Computer Science (EEMCS), University of Twente, 7522NB Enschede, The Netherlands

**Keywords:** freezing of gait, Parkinson’s disease, functional near-infrared spectroscopy (fNIRS), cortical activity, stopping

## Abstract

Freezing of gait, characterized by involuntary interruptions of walking, is a debilitating motor symptom of Parkinson's disease that restricts people's autonomy. Previous brain imaging studies investigating the mechanisms underlying freezing were restricted to scan people in supine positions and yielded conflicting theories regarding the role of the supplementary motor area and other cortical regions. We used functional near-infrared spectroscopy to investigate cortical haemodynamics related to freezing in freely moving people. We measured functional near-infrared spectroscopy activity over multiple motor-related cortical areas in 23 persons with Parkinson's disease who experienced daily freezing (‘freezers’) and 22 age-matched controls during freezing-provoking tasks including turning and doorway passing, voluntary stops and actual freezing. Crucially, we corrected the measured signals for confounds of walking. We first compared cortical activity between freezers and controls during freezing-provoking tasks without freezing (i.e. turning and doorway passing) and during stops. Secondly, within the freezers, we compared cortical activity between freezing, stopping and freezing-provoking tasks without freezing. First, we show that turning and doorway passing (without freezing) resemble cortical activity during stopping in both groups involving activation of the supplementary motor area and prefrontal cortex, areas known for their role in inhibiting actions. During these freezing-provoking tasks, the freezers displayed higher activity in the premotor areas than controls. Secondly, we show that, during actual freezing events, activity in the prefrontal cortex was lower than during voluntary stopping. The cortical relation between the freezing-provoking tasks (turning and doorway passing) and stopping may explain their susceptibility to trigger freezing by activating a stopping mechanism. Besides, the stopping-related activity of the supplementary motor area and prefrontal cortex seems to be out of balance in freezers. In this paper, we postulate that freezing results from a paroxysmal imbalance between the supplementary motor area and prefrontal cortex, thereby extending upon the current role of the supplementary motor area in freezing pathophysiology.

## Teaser

Cockx *et al*. investigated freezing-related cortical activity during free ambulation in people with Parkinson’s disease using functional near-infrared spectroscopy. Their results suggest that freezing results from an oversensitivity to stopping signals and an imbalanced activity between the supplementary motor area and the prefrontal cortex within the stopping network.

## Graphical Abstract

**Graphical Abstract:**
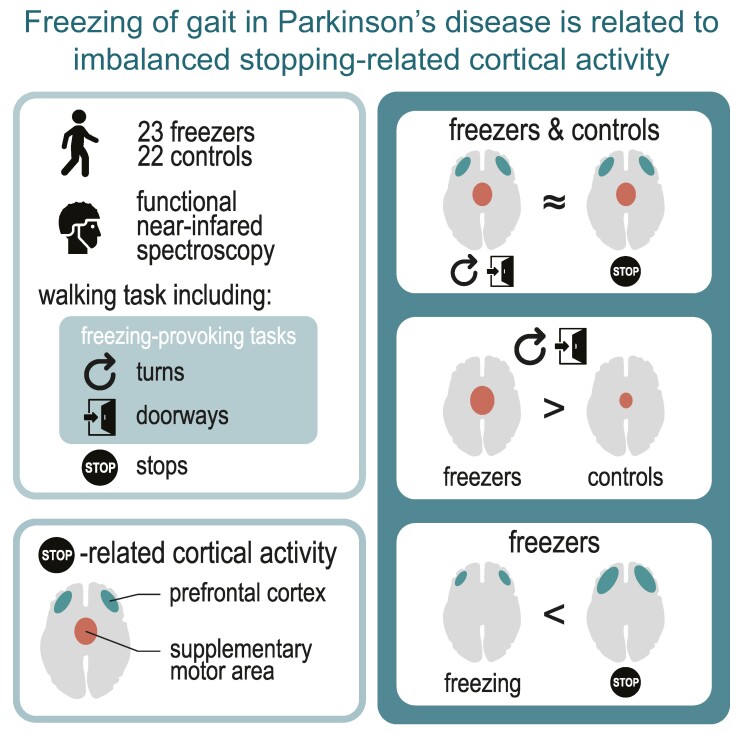

## Introduction

Normally, we initiate and stop walking as voluntary actions. However, 30–60% of the people with Parkinson's disease experience sudden interruptions of gait that are not voluntary.^1-3^ This is called ‘freezing of gait’ and is considered one of the most debilitating symptoms of Parkinson's disease,^4^ leading to reduced mobility, falls, fear of falling and social exclusion.^5-9^ Freezing occurs frequently during situations where motor programmes must be adapted, such as during turning, walking through doorways, approaching a destination and starting to walk.^4,10^ Standard dopamine replacement therapy is rarely sufficient to treat freezing, and the development of novel therapies is hampered by our limited knowledge of the underlying brain mechanisms.^11-14^ The current study aims to get to a better understanding of the cortical mechanisms underlying freezing of gait when triggered by changes in motor programmes.

Previous research converges on the hypothesis that various triggers of freezing activate an as-of-yet-unknown cortical network, eventually activating a common pathway in the basal ganglia and brainstem nuclei.^10,15,16^ Within this final pathway, the globus pallidus internus (GPi) and the substantia nigra pars reticularis (SNr) transiently increase their inhibitory output, leading to decreased activity in gait-controlling brainstem nuclei and eventually uncoordinated firing of the central pattern generators. Although the excessive GPi–SNr output seems to play a key role, there is evidence that the actual problem of freezing lies higher up in the cerebral cortex and the cortico-basal connections, which are responsible for a flexible adaptation of gait.^12,15^ Several theories have been proposed to explain this cortical mechanism (for a review, see Bardakan *et al.*^16^). For instance, excessive activation of the supplementary motor area may temporarily recruit the hyperdirect pathway, activating the GPi–SNr via the subthalamic nucleus.^17,18^ Alternatively, conflicting cortical processes in motor, cognitive and limbic cortical areas could transiently overwhelm the striatum, thereby losing its inhibitory control over the GPi.^19^

Many of the theories on freezing of gait pathophysiology are based on neuroimaging methodologies that only allowed one to study the neural correlates of gait indirectly while participants were lying supine in confined spaces. For example, functional MRI studies rely on the imagination of gait or the mimicking of gait with foot pedals while navigating through a virtual environment.^20-22^ PET scanners can only study gait on a coarse time scale as they are restricted to scan the brain after completion of a gait task.^23,24^ Although these studies have improved our understanding of freezing of gait mechanisms, the question remains on how to translate these findings to ‘real’ gait.

In the present study, we use functional near-infrared spectroscopy (fNIRS) to measure cortical activity related to freezing of gait in Parkinson's disease during free ambulation. fNIRS is a wearable neuroimaging technique that—similar to functional MRI (fMRI)—is sensitive to changes in local haemodynamics.^25,26^ It measures changes in oxygenated (HbO) and deoxygenated haemoglobin (HbR) by transmitting infrared light through the cortex at prespecified locations. Unlike previous studies that mainly focused on the prefrontal cortex,^27-30^ we assessed fNIRS activity over multiple cortical areas that have previously been associated with freezing: the premotor cortex (PMC), the supplementary motor area (SMA), the prefrontal cortex (PFC), the posterior parietal cortex (PPC), and the primary motor cortex (M1).^16^

The goal of this work was two-fold. We first investigated whether people with Parkinson's disease who experience freezing (‘freezers’) recruited different cortical areas than age-matched controls when voluntarily stopping and when successfully (i.e. without freezing) performing gait tasks involving changes of motor programmes: making 180° turns, passing through a doorway and starting to walk. We hypothesized that the people with Parkinson's disease would display increased activity in higher-order cortical areas (such as the SMA, PMC, PFC and PPC) than the controls to compensate for their loss of automatic motor control.^31-33^ Second, within the group of freezers, we examined which cortical areas showed freezing-related activity and how their activity differed from stops and from the same gait events without freezing (e.g. successful turns and doorway passages). Based on a previous fNIRS^27^ and fMRI^34^ study, we expected to observe ‘higher’ activity in the PFC during freezing than during the other gait events.

## Materials and methods

### Participants

We recruited participants (age > 18 years) with the help of ParkinsonNEXT (https://www.parkinsonnext.nl), a Dutch online platform connecting people with Parkinson's disease to researchers. We included participants who were diagnosed with idiopathic Parkinson's disease according to accepted international standards^35,36^ and who subjectively reported freezing at least once a day. We did not differentiate between OFF- or ON-state freezing as an inclusion criterion as we did not formally evaluate freezing frequency in the OFF and ON states. However, almost all participants declared to have more or the same amount of freezing OFF medication. We encouraged the participants with Parkinson's disease to bring their partners, relatives or friends to serve as healthy control. The healthy controls were matched at the group level for age and gender to the Parkinson’s disease group. We used the following exclusion criteria: comorbidities causing severe gait impairments; comorbidities that could interfere with the fNIRS recording such as structural brain lesions or previous brain surgery (including deep brain stimulation); and inability to comply with the protocol including severe cognitive impairment as judged by a clinician (HMC). Absence of objective freezing during the study protocol was not an exclusion criterion. In total, 3 of the 25 Parkinson’s disease participants did not show freezing during the protocol. Two of them were later validated as being freezers based on home-made videos.

The participants with Parkinson’s disease performed all procedures in the OFF state, following at least 12 h overnight withdrawal of anti-Parkinson medication.

A trained clinician (HMC) assessed motoric symptom severity by the Movement Disorders Society Unified Parkinson's Disease Rating Scale part III (MDS-UPDRS III)^37^ at the start of the lab visit (OFF medication). The other questionnaires and tests were completed after the gait tasks: the New Freezing of Gait Questionnaire,^38^ the Montreal Cognitive Assessment,^39^ the Trail Making Test parts A and B,^40^ the Hospital Anxiety and Depression Scale,^41,42^ and a supplementary set of questions asked for feelings of anxiety and insecurity during the different parts of the walking task (e.g. ‘I felt insecure/anxious when walking through the door’).

In total, we recruited 24 people for the healthy control (HC) group and 25 people for the Parkinson’s disease group following previous recommendations for fNIRS gait studies.^32,43^ The data from 22 HC and 23 Parkinson’s disease participants were included in the final analysis. Reasons to exclude the data from the other four participants were as follows: two HC participants were excluded because of technical issues with the motion capture system; one person with Parkinson’s disease was not able to complete the protocol due to fatigue; and one person with Parkinson’s disease showed a poor quality of the fNIRS data (>50% of the channels have poor signal quality).

The medical ethics committee of Arnhem–Nijmegen approved the study (NL70915.091.19). All procedures were conducted in accordance to the Declaration of Helsinki and the Medical Research Involving Human Subjects Act (WMO). Data handling followed the General Data Protection Regulation (EU GDPR). All participants provided written informed consent before participating. They had the possibility to opt-in for data sharing of their de-identified research data.

### Gait task

**Figure 1:**
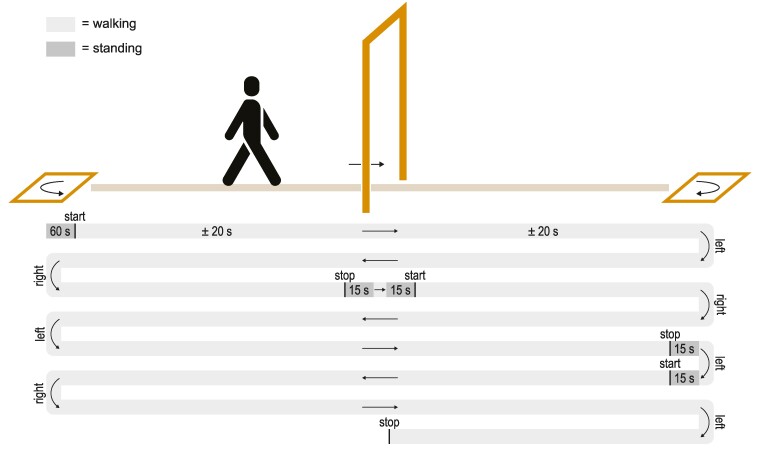
**Gait task.** Example of one run (±6.5 min) of the gait task. Participants walk up and down a corridor, halfway passing through a narrow doorway frame (60 cm wide), and at the ends making 180° turns in a square taped on the floor (50 cm wide). The distance between the doorway and the square was set at an individualized walking distance of ∼20 s. Each 2.5 corridor length, participants were instructed to stop in front of the door or in the square, to make one step through the door or turn around after 15 s, and then to resume walking after another 15 s. The direction of the turns was alternated between each side of the corridor and this direction was switched after each instructed stop. Each participant completed approximately four runs in total.

The gait task involved walking at a comfortable pace in a long corridor (Fig. 1). Halfway through the corridor, the participants passed through a narrow doorway frame of 60 cm wide. At the ends of the corridor, they made 180° turns in a 50 cm wide square taped on the floor. After every passage of two doors and two turns (i.e. every 2.5 corridor lengths), they were instructed to stop in front of the door, or in the square for 30 s. The regularity of these intervals was introduced to minimize the unpredictability of stops. Halfway through the 30 s, they were instructed to make one step through the door or to turn around for 180°, and then resume walking. All participants practiced the walking paradigm. The distance between the door and the turns was individualized based on the walking speed during the practice trial to achieve a walking duration of approximately 20 s. This ensured a stable walking baseline of fNIRS activity between two gait events (e.g. a doorway passage and a turn).^43^

To minimize cognitive load, instructions to stop or keep walking, or to turn left or right, were given approximately 2 m (i.e. ±2 s) before encountering the door or the square. The direction of the turn, i.e. leftwards or rightwards, was alternated between the squares. To guarantee an equal number of turning directions at each side of the corridor, we switched the turning directions after each instructed stop (see Fig. 1). Participants were discouraged to use any compensation strategy to improve their walking and were instructed not to talk or make extra movements such as scratching their head during the gait tasks. If the participants experienced a freezing episode, they were instructed to continue the task to the best of their ability.

In total, the participants completed four identical runs of approximately 6.5 min each, with standing upright for 60 s at the beginning of each run to avoid changes in orthostatic blood pressure that could confound the fNIRS recordings. Between each run, they could rest as long as needed. Participants walked unaided but were always accompanied by a researcher to prevent them from falling.

### Materials

#### Functional near-infrared spectroscopy

**Figure 2:**
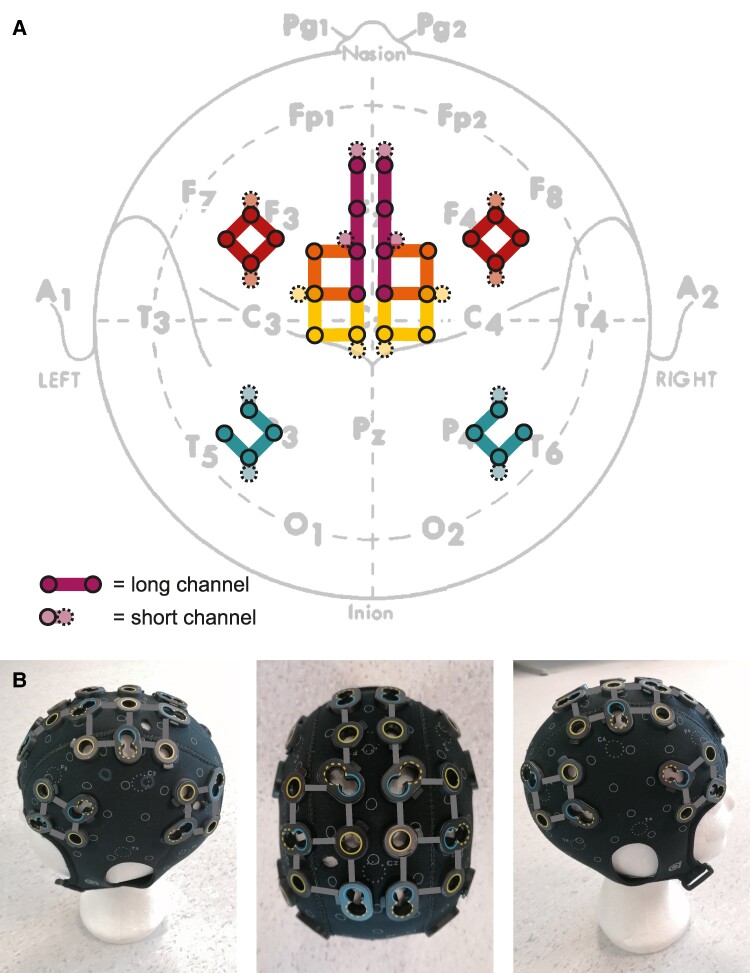
**fNIRS cap layout. (A)** Schematic representation of the cap layout indicating the positions of the long channels (30 mm interoptode distance) and short channels (10 mm interoptode distance) relative to the 10–20 EEG reference system and the main sulci. The recorded channel positions are presented in Supplementary Fig. 7 and Table 3. **(B)** Images of the cap with the detectors in blue and the sources in yellow (dashed line for low output source). M1, primary motor cortex (yellow); PMC, premotor cortex (orange); SMA, supplementary motor area (purple); PFC, prefrontal cortex (red); PPC, posterior parietal cortex (green).

Two continuous-wave fNIRS devices (Brite24, Artinis Medical Systems) were combined into one cap. Each device consisted of 8 photodiode detectors sampling at a 50 Hz rate and 16 light-emitting diode emitters with nominal wavelengths of 760 and 850 nm. These detectors and emitters (‘optodes’) were placed in a neoprene cap (headcap with print, size M or L, Artinis Medical Systems) with custom-made holes according to the layout as shown in Fig. 2. In total, there were 32 long channels with an interoptode distance of 30 mm and 16 short channels with an interoptode distances of 10 mm. Short channels only penetrate the superficial layers of the scalp and are intended to record and correct for physiological systemic artefacts such as changes in heart rate, blood pressure and breathing.^44,45^

The cap was designed to optimally cover the areas previously associated with freezing,^16,34,46-49^ however, at the same time avoiding interference between the optodes of the two devices that would happen if placed too close to each other. The cap was placed according to the 10–20 electrode placement scheme with Cz as the midpoint between the nasion and inion, and between the left and right pre-auricular points. We used an optical 3D scanner (Structure Sensor, Occipital) to record the optode positions on each participant's head.^50^ We calculated the Montreal Neurological Institute (MNI) coordinates of each channel and estimated their underlying brain regions defined by automated anatomical labelling to check whether the channels indeed corresponded to the intended regions of interest^51^ and to allow for a comparison to previous studies^16,34,46-49^ (see Supplementary material).

The fNIRS devices connected wirelessly via Bluetooth to a laptop running the Artinis recording software (OxySoft 3.2.70). The quality of the data was checked prior to recording and during the breaks in between the runs.

#### Motion capture

Seventeen inertial measurement units (MVN Awinda system, Xsens) were attached to the body with Velcro straps. The data, sampled at 60 Hz, was wirelessly transmitted to a laptop running the Xsens recording software (MVN Awinda, version 2020.0.1). This software synchronizes the 17 motion sensors and performs offline processing, resulting in full-body movement data, including the position and orientation of all body parts, as well as the acceleration and angular velocity of each sensor.

#### Video

Two video cameras (Canon Legria HFG26, sampling rate 25 Hz) were placed at each end of the corridor, ∼2 m behind the turning squares and directed towards the doorway. A third camera was mounted on a wheelchair following the participant during the gait tasks. All cameras targeted the legs and feet of the participant, to avoid unnecessary participant identification.

#### Synchronization

We synchronized the fNIRS, movement and video data offline using sync events that were sent simultaneously to the different recording devices. For fNIRS, these sync events consisted of Lab Streaming Layer (LSL) markers (https://github.com/sccn/labstreaminglayer, accessed on: 3-4-2023) that were registered by the fNIRS recording software. For the movement data, simultaneous with the LSL markers, we created Transistor-Transistor Logic pulses that were recorded by the Xsens hardware. For the video data, the sync events consisted of audio beeps (inaudible to the participant) that were recorded along with the video via the external microphone input. Given that the experimental setup spanned a corridor of about 50 m long, we sent out the sync events using ZeroMQ (https://github.com/zeromq, accessed on: 3-4-2023) over a local network connected to the different recording devices either wirelessly (video) or via ethernet cable (fNIRS, Xsens).

### Functional near-infrared spectroscopy data pre-processing

The fNIRS data were loaded into MATLAB and pre-processed using a combination of FieldTrip,^52^ Homer3^53^ and custom functions, minimally following previous recommendations for fNIRS studies.^26,43^ We used pilot data (from two young adult subjects) to construct the pre-processing pipeline and to define pre-processing parameters (e.g. for motion artefact correction) and adjusted those parameters based on the first three to five participants of our dataset.

The fNIRS data were first resampled from 50 to 60 Hz to match the movement data. For each run, we removed channels that were too low in quality, defined as a signal quality index of less than two for more than half of the time the participant was standing still.^54^ On average, 15% of the long channels (four to five channels per participant) and 16% of the short channels (two to three channels per participant) were removed. Motion artefacts were corrected with the combination of a movement artefact correction algorithm using spline interpolation (*stdv threshold* = 65; *amplitude threshold* = 0.05; *tMotion* = 0.5; *tMask* = 1)^55^ and a wavelet correction (*IQR* = 0.8).^56-59^ Optical densities were converted to haemoglobin concentration changes based on the modified Beer–Lambert–Law with a differential path length factor adapted to age and wavelength.^60^

We removed slower confounding factors, such as systemic artefacts induced by physical activity^61,62^ and head movements,^63^ by regressing out short-channel data and movement data of the head (see also Box 1).^64-66^ We first applied a 0.5 Hz low-pass filter (third-order Butterworth) to both the fNIRS and movement data to remove faster noise components (e.g. heartbeats and footsteps). For regression of the short channels, we z-transformed the signals and used the first eight components of a principal component analysis (both HbO and HbR), representing >90% of the variation in these channels. For regression of the movement data, we used the z-transformed acceleration of the head, the angular acceleration of the head and the orientation of the head relative to the neck, as we observed that participants looked down while performing the gait tasks (Supplementary Fig. 1). For each long channel, we performed an ordinary least square regression with the long channel as the dependent variable and the short channels and movement data as regressors. The residuals of the regression analysis were used for further analysis.

Box 1 **fNIRS requires careful correction for confounds of walking**

The measurement of cortical signals during gait with fNIRS is now possible, thanks to modern equipment, but still not trivial.^43^ First, walking induces large **‘*systemic physiological confounds****’* such as changes in heart rate, blood pressure and breathing patterns.^61,62^ Systemic physiological changes confound the fNIRS signals as they induce direct changes in the haemoglobin concentrations intra- and extracerebrally.^67,68^ Second, walking introduces movement artefacts due to head movements, which can be separated into direct and indirect movement artifacts.^63^ ‘***Direct movement artefacts***’ are caused by direct mechanical decoupling between the optodes and the scalp, allowing environmental light to enter the optical path, and induce spikes and baseline shifts in the signal.^69^ ‘***Indirect movement artefacts***’ are caused by local blood flow shifts due to head changes in head position and induce slower confounding signals.^66^

We corrected for the potential confounds of walking by performing a regression analysis with 16 short channels intended to only measure superficial scalp haemodynamics (systemic confounds) and with movement data of the head including acceleration, angular acceleration and orientation (indirect movement artefacts). Direct movement artefacts were corrected with a combination of spline interpolation^55^ and wavelet correction,^56^ which is currently considered the best method for direct movement artefact correction.^57-59^ Of note, we observed that participants looked down when stopping, turning, freezing and even when passing a doorway (Supplementary Fig. 1). We accounted for this by including head orientation in the regression analysis, though there is currently no standard practice for this in fNIRS research. The effects of the systemic artefact correction and indirect movement artefact correction are displayed here above and are not to be underestimated: the conclusions of this paper could have been considerably different had the confounds not been considered.

The increase in M1 activity during walking and decrease in activity during stopping are in line with our expectations and consistent with previous gait studies using SPECT, fMRI or fNIRS.^24,70-73^ The involvement of the SMA and PFC (IFG) during stopping is easy to link to the general stopping network^74^ and has also previously been associated with imagined termination of gait.^75^ Nevertheless, we cannot rule out that the current results still hold some bias. For example, we did not account for the non-instantaneousness of indirect movement artifacts^65,66^ or did not directly account for cardiorespiratory changes by simultaneously measuring heart rate, blood pressure or breathing behaviour during the protocol.^76^ Future studies should take into account these potential sources of bias.

**Figure:**
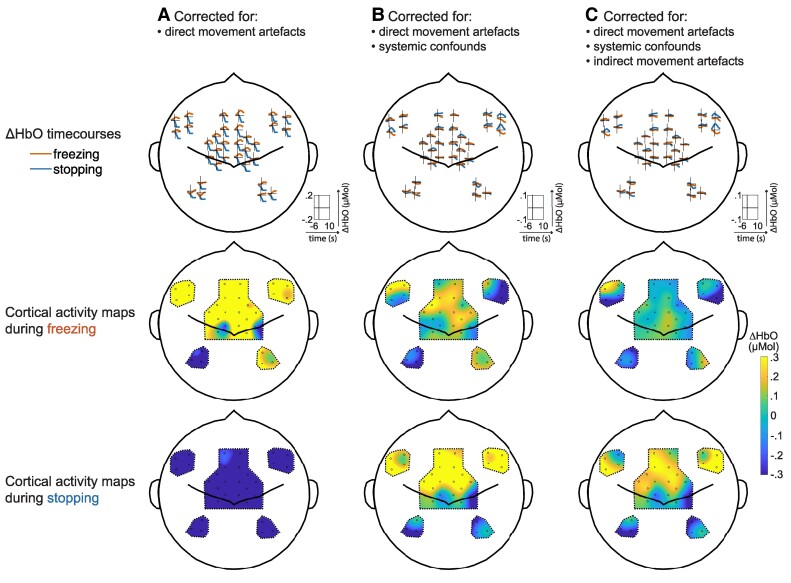


**The necessity of correction for confounds.** (A) ΔHbO time courses and cortical activity maps during freezing (red line) and stopping (blue line) when only corrected for direct movement artefacts with spline interpolation and wavelet filtering. The cortical activity maps represent the average ΔHbO response 0–3 s after freezing and stopping onset. (B) ΔHbO time courses and cortical activity maps when additionally corrected for systemic confounds with short channels. (C) ΔHbO time courses and cortical activity maps when additionally corrected for indirect movement artefacts with motion data from the head.

Subsequently, we applied a 0.01 Hz high-pass (second-order Butterworth) and a 0.1 Hz low-pass (sixth-order Butterworth) filter to remove remaining noise components. The cut-off values of these filters were chosen by checking the power spectra plots of each participant individually to optimally remove slow drifts and Mayer waves while retaining the relevant task frequency.^77^

Finally, we z-transformed each fNIRS channel to be able to average over multiple channels belonging to the same cortical region, obtaining normally distributed data. We calculated z-scores by subtracting the mean HbO or HbR values of each channel and each run, and by dividing those by their standard deviation.

### Gait events

Freezing of gait events were annotated on video by two independent trained raters (HMC and YAFR) in the ELAN software (https://archive.mpi.nl/tla/elan, accessed on: 3-4-2023*)*, as described by Gilat.^78^ We defined freezing as ‘a brief, episodic absence or marked reduction of forward progression of the feet despite the intention to walk’.^4^ The end of an episode was marked as the last toe-off after which the participant was able to perform at least two effective alternating steps.^78^ The annotations of both raters were subsequently compared and combined using FOGtool^79^ (*tolerance* = 2 s; *correction* = include). The positive agreement between the raters was 0.86 and the negative agreement 0.98, with a prevalence index of −0.74. Remaining non-overlapping annotations were discussed until consensus was reached. Freezing events were only considered for analysis when not preceded by another freezing event within 10 s. In total, 550 freezing events reached consensus, of which 104 were excluded.

We defined successful gait events as turns, doorway passages, starts or stops without any freezing event 10 s before or 10 s after the event. The successful gait events were defined by the movement data and were checked for correctness by looking at the videos. A detailed description of this process can be found in the Supplementary material and Supplementary Fig. 2. In total, 1231 normal gait events for the Parkinson’s disease group and 1596 normal gait events for the HC group could be used for final analysis.

### Statistical analysis

We analysed the fNIRS data with Bayesian hierarchical models in Rstudio (RStudio 2022.07.2; Rstudio, Inc., Boston, MA) using the brms package (version 2.18.0).^80^ Two types of statistical models were built: one to compare the cortical activity of the Parkinson’s disease group to the HC group for the various gait events when no freezing occurred (e.g. stop in Parkinson’s disease versus HC; successful turn in Parkinson’s disease versus HC) and one to compare the cortical activity during a freezing event to a voluntary stop and to a successful event of the same type as the freezing event (e.g. turning freeze versus stop versus successful turn). Note that each Parkinson’s disease participant contributed a various number of freezing events and successful gait events, yielding an imbalanced data design. Hierarchical models account for imbalanced data by ‘shrinkage’, meaning that the data from participants contributing less or more variable data are pulled towards the group mean.^81,82^ Moreover, hierarchical models estimate all effects simultaneously. This implies that we can inspect the effects of the studied factors within a model directly, for example, when looking at the cortical activity in the Parkinson’s disease and HC groups individually, thereby increasing statistical sensitivity and removing the need to correct for multiple comparisons.^81,83^

The main outcome variable was the mean HbO within a region of interest (ROI) during the different event types. For each channel, we calculated the average HbO from 0 to 3 s after the event onset (i.e. onset of stop, turn, doorway, start or freezing). For stopping and starting, we additionally calculated the average HbO from 7 to 10 s after the stopping and starting events to assess cortical activity during standing and walking. All values were baseline-corrected by subtracting the average HbO from 10 to 5 s before the event onset. This baseline was chosen to contain a stable reference signal during walking (e.g. turns, doors or stops) or standing (e.g. starts), yet before the instructions were given to stop, start or keep walking. Subsequently, we averaged the HbO values of the channels belonging to the same ROI corresponding to the layout of Fig. 2. Time courses of HbO and HbR values were visually checked to contain reliable haemodynamic responses (Supplementary Fig. 6), but no statistical analysis was performed on the HbR values.

The first type of model included a fixed intercept, representing the global activity during the gait event compared to baseline, and a fixed effect for group (sum-contrast-coded), representing the difference in activity between the Parkinson’s disease and HC groups. A random intercept for participant accounted for individual differences in cortical activity. Left and right turns were pooled as they did not display significant direction effects, nor interaction effects with the hemisphere. The second type of model included a fixed intercept (activity versus baseline), a fixed effect for condition (freeze versus stop versus successful gait event; sum-contrast-coded), and a random intercept for participant and a random slope for condition varying over participants. We only computed models for freezing triggered by turns or doorways that occurred during walking, as we did not have enough data for the other freezing types. We investigated differences in timing between the onset of the freezing events and the onset of the stop or successful gait events, to assess whether these events could be compared reliably. The turning freezing occurred mostly at the start of the turn [median (IQR): 11% (0.27%)] and the doorway freezing approximately 0.3 m before the door [median (IQR): 0.27 (0.52 0.14); Supplementary Fig. 3]. Considering the slowness of the haemodynamic signal, we concluded that the difference in timing was negligible. We executed a model for each type of event (Model Type 1) or freezing type (Model Type 2) and for each ROI separately. Additionally, to visualize the results on cortical activity maps, we calculated a model for each channel.

To assess whether fNIRS activity during the successful gait events in the Parkinson’s disease group was specifically related to freezing, we exploratively fitted a third type of model for this group. This model included a fixed intercept, a fixed effect for % time frozen (specifically triggered by the studied gait event), a fixed effect for MDS-UPDRS motor score, and a random intercept per participant. The % time frozen (i.e. the % time spent with freezing relative to the total time of the gait task) and MDS-UPDRS scores were centred and standardized before model fitting.

Posterior probabilities of the model parameters were estimated with Markov chain Monte Carlo sampling, starting from flat priors. Details on the sampling procedure, the priors, and the checks that were performed are provided in Supplementary Tables 1 and 2. We visualize the probability density functions of the estimated effects and report the 95% credibility intervals (CrIs), calculated from quantiles of the probability functions. We consider the 95% CrI as the probability threshold to make claims about the sign of the fNIRS activity (i.e. being higher or lower than its control condition).^84-86^ Note that posterior probabilities from Bayesian models directly express the belief of the underlying studied effects, given the data. They are therefore more intuitive and less prone to Type I errors than classical *P*-values from frequentist statistics, which are calculated based on hypothetical replications of the experiment.^87,88^

## Results

### Participants

Table 1 displays the characteristics of the included participants. On average, the Parkinson’s disease group (Hoehn and Yahr Stages 2–3) had a disease duration of 8.0 ± 3.9 years and an MDS-UPDRS III score of 43.7 ± 10.5 (max. score 132). They all reported to have moderate-to-severe freezing in daily life with a NFOGQ score of 19.4 ± 3.5 (max. score 28). The two groups did not differ significantly in age, gender or cognition, but the Parkinson’s disease group scored significantly higher for questions inquiring about levels of anxiety or depression.

**Table 1:** Participant characteristics

	PD (*n* = 23)	HC (*n* = 22)	*P*-value*
Age (years)	66.6 ± 8.9	65.9 ± 10.1	0.82
Sex (% man)	87%	82%	/
Hand dominance (% right-handed)	91%	86%	/
NFOGQ	19.4 ± 3.5	/	/
% time frozen	13.0 ± 17.4	/	/
MDS-UPDRS part III	43.7 ± 10.5	/	/
Disease duration (years)	8.0 ± 3.9	/	/
Levodopa Equivalent Daily Dose (mg)	1080.3 ± 345.2	/	/
Years of education	17.4 ± 5.8	15.1 ± 4.5	0.15
MoCA	25.9 ± 3.0	26.3 ± 3.2	0.67
TMT part B – A (s)	71.4 ± 103.6	43.9 ± 46.4	0.27
HADS	8.5 ± 5.2	4.2 ± 4.7	0.007
Anxiety levels	2.7 ± 12.0	0.2 ± 0.5	<0.001

Values indicate mean ± standard deviation.

MoCA, Montreal Cognitive Assessment (range, 0–30); TMT, Trail Making Test; HADS, Hospital Anxiety and Depression Scale [range, 0–42; anxiety levels (range 0–21)]; MDS-UPDRS, Movement Disorders Society's Unified Parkinson's Disease Rating Scale (range, 0–132); NFOGQ, New Freezing of Gait Questionnaire (range, 0–28); % time frozen, the % time spent with freezing relative to the total time of the gait task.

^*^ *P*-values of two-sample *t*-test.

The median number of freezing episodes per Parkinson’s disease participant that were observed during the gait task was 15 (IQR 3.25–32.75, range 0–82) including the three participants that did not experience any freezing during the protocol. Fifty per cent of the episodes were triggered by turning (in 19 of 23 participants), 24% by doorway passing (12 participants), 16% by destination freezing (9 participants), 6% by starting (5 participants) and 5% during straight walking. Regarding the phenotypical presentation, 92% of the episodes were of the trembling–shuffling type and 8% of the akinetic type (14 participants trembling–shuffling only, 1 participant akinesia only, and 5 participants mixed). The median duration of a freezing episode was 3.8 s (IQR 1.9–7.2 s, range 0.4–263.8 s).

### Parkinson’s disease versus healthy control groups

#### Stopping and standing

**Figure 3:**
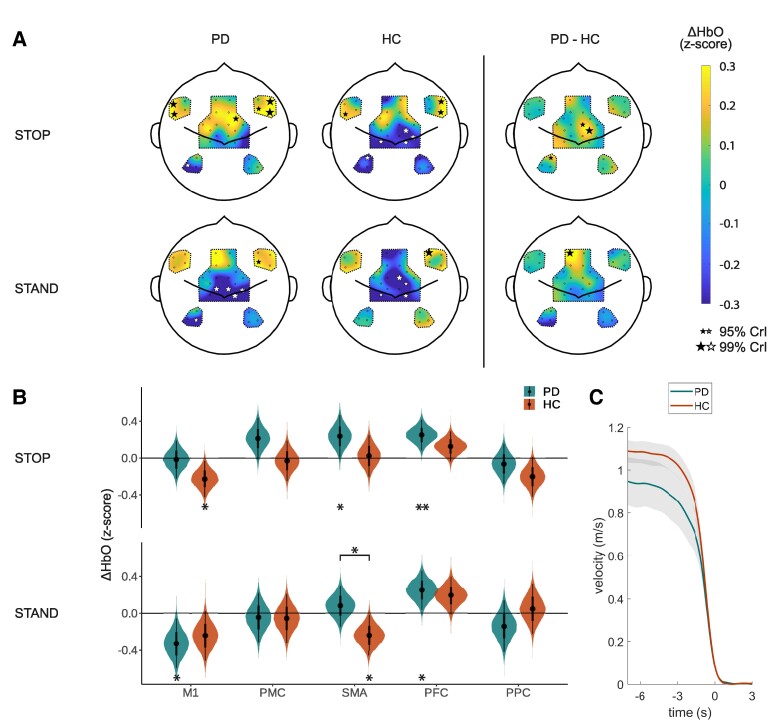
**Stopping and standing. (A)** Cortical activity maps of estimated ΔHbO responses 0–3 s after stopping (stop) and 7–10 s after stopping (stand) compared to baseline (−10 −5 s) as calculated by the Bayesian hierarchical model (N_PD_ = 21, N_HC_ = 22). The black and white stars indicate channels with 95% CrIs of the posterior probabilities excluding zero (small star) or 99% of the CrI excluding zero (large star). The size of the star scales with the probability that the estimated ΔHbO excludes zero. **(B)** Posterior probabilities of the estimated average ΔHbO responses for each ROI as calculated by the Bayesian hierarchical model (N_PD_ = 21, N_HC_ = 22). The stars underneath the violin plots indicate if the posterior probability of the estimated ΔHbO response is different from baseline; the stars above the violin plots indicate if the posterior probability of the estimated ΔHbO response differs between the groups (* = 95% CrI excluding zero; ** = 99% CrI excluding zero). (**C**) Average walking velocity for the two study groups when stopping (*t* = 0 s). The grey areas indicate the 95% confidence intervals. PD, Parkinson's disease group; HC, healthy control group; ΔHbO, change in oxygenated haemoglobin; CrI, credibility interval; ROI, region of interest; M1, primary motor cortex; PMC, premotor cortex; SMA, supplementary motor area; PFC, prefrontal cortex; PPC, posterior parietal cortex.

Figure 3A shows the cortical activity of the Parkinson’s disease and HC groups during stopping (0–3 s after stop event) and standing (7–10 s after stop event). During stops, the Parkinson’s disease group displayed widespread activity in the premotor and prefrontal areas, while the HC group showed similar but more focused activity in these areas. Posterior probabilities of the estimated ΔHbO responses in the different ROIs (Fig. 3B) revealed that the Parkinson’s disease group increased activity in the SMA [mean (95% CrI) 0.24 (0.01 0.46)] and PFC [0.25 (0.09 0.42)}, while the HC group decreased activity in the M1 during stopping [−0.23 (−0.42 −0.03)]. When standing still, M1 activity globally was decreased compared to baseline levels for both groups [global intercept −0.29 (−0.48 −0.09)]. The SMA showed substantive differences between the Parkinson’s disease group and the HC group, with higher activity in the Parkinson’s disease group than in the HC group [group 0.16 (0.00 0.31)].

On average, the Parkinson’s disease group walked slower than the HC group (Parkinson’s disease, 0.95 m/s; HC, 1.09 m/s; two-sample *t*-test, *P* = 0.04) and decelerated slower than the HC group in the last 3 s before coming to a stop (Parkinson’s disease, −0.24 m/s^2^; HC, −0.30 m/s^2^; two-sample *t*-test, *P* = 0.008; Fig. 3C).

#### Turning and doorways

**Figure 4:**
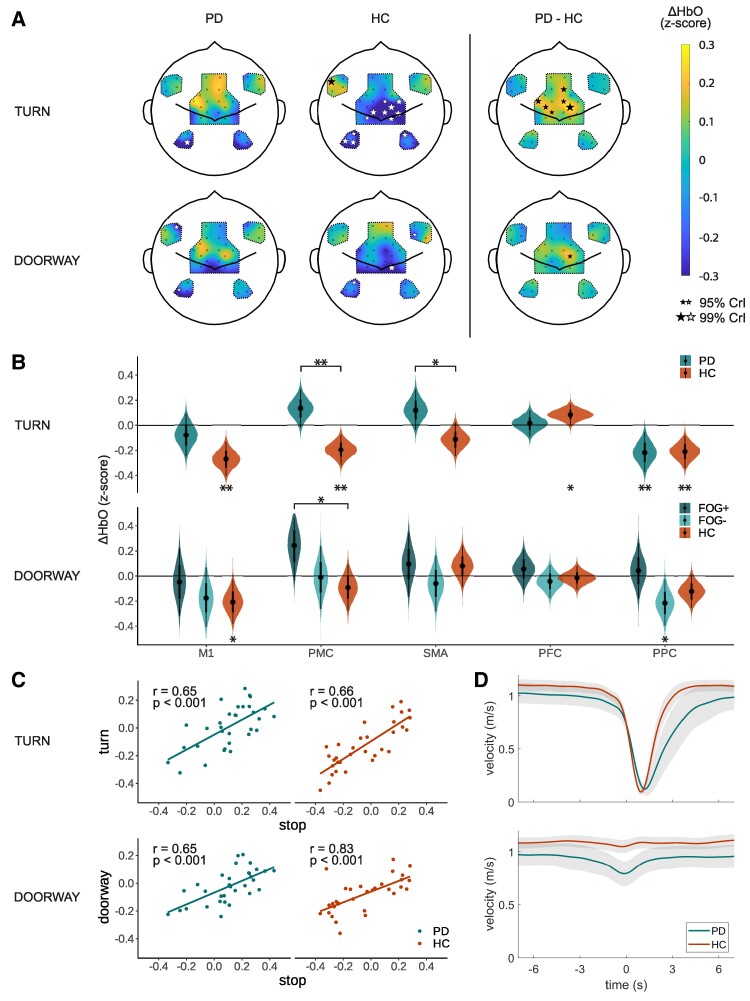
**Turns and doorways. (A)** Cortical activity maps of estimated ΔHbO responses 0–3 s after turning and 0–3 s after doorway passing compared to baseline (−10 −5 s) as calculated by the Bayesian hierarchical model (turn, N_PD_ = 16, N_HC_ = 22; doorway, N_PD_ = 22, N_HC_ = 22). The black and white stars indicate channels with 95% CrI of the posterior probabilities excluding zero (small star) or 99% of the CrI excluding zero (large star). The size of the star scales with the probability that the estimated ΔHbO excludes zero. **(B)** Posterior probabilities of the estimated average ΔHbO responses for each ROI as calculated by the Bayesian hierarchical model (turn, NPD = 16, NHC = 22; doorway, NFOG+ = 10, NFOG− = 13, NHC = 22). The stars underneath the violin plots indicate if the posterior probability of the estimated ΔHbO response is different from baseline; the stars above the violin plots indicate if the posterior probability of the estimated ΔHbO response differs between the groups (* = 95% CrI excluding zero; ** = 99% CrI excluding zero). For the doorway condition, we present the subgroup analysis which the Parkinson’s disease group split into participants that experienced doorway freezing during the study (FOG+, *n* = 10, darker green) and that did not experience doorway freezing (FOG−, *n* = 13, lighter green). The original analysis is presented in Supplementary Fig. 5. **(C)** Correlations between the estimated cortical activity of each channel during stopping (*x*-axis) and during turns/doorways (*y*-axis). Each dot represents a different channel. Given statistics are based on the Pearson correlation coefficient (*N* = 32 channels). **(D)** Average walking velocity for the two groups when turning (upper plot) and when walking through the doorway (lower plot). The shaded areas indicate the 95% CrIs. Timepoint zero represents the onset of the turn/doorway passage. For abbreviations, see Fig. 3.

The Parkinson’s disease group showed more extensive and higher activity of the premotor areas during turning than the HC group (Fig. 4A), with Bayesian statistics providing evidence for higher activity in the PMC [group, 0.17 (0.07 0.26)] and the SMA [group, 0.12 (0.01 0.23); Fig.4B]. Similarly, the Parkinson’s disease group showed higher activity during doorway passage in the PMC; however, this effect was smaller, and the 95% CrI did not exclude zero [group, 0.04 (−0.08 0.15); Supplementary Fig. 5]. Because not all participants experienced doorway freezing during the task, we performed a follow-up analysis in which we split the Parkinson’s disease group in a subgroup that experienced doorway freezing (FOG+, *n* = 12) and a subgroup that did not experience doorway freezing during the protocol (FOG−, *n* = 11). This follow-up analysis revealed a higher PMC activity in the FOG+ group than in the HC group during doorway passage [group, 0.34 (0.00 0.68); Fig. 4B].

Interestingly, we observed a remarkable resemblance between the cortical activity maps of turning and doorway passage on the one hand (Fig. 4A) and the cortical activity maps of stopping on the other hand (Fig. 3A). Therefore, we exploratively plotted the model estimates of each channel during stopping versus the model estimates of each channel during turning/doorway passage (Fig. 4C) and calculated the Pearson correlation coefficients between the two. This yielded significant correlations (*P* < 0.001) with correlation coefficients of > 0.65 in both study groups.

Analysis of the walking speed showed that the Parkinson’s disease group decelerated slower than the HC group in the 2 s ‘before’ turning (HC, −0.15 m/s^2^; Parkinson’s disease, −0.09 m/s^2^; unpaired *t*-test, *P* = 0.009) and that the Parkinson’s disease group turned slower than the HC group (Parkinson’s disease, 3.07 s; HC, 1.89 s; unpaired *t*-test, *P* < 0.001; Fig. 4D). During doorway passage, the Parkinson’s disease group significantly reduced their walking speed 2 s before encountering the doorway (−0.04 m/s^2^; one-sample *t*-test, *P* < 0.001) while the HC group maintained the same speed (−0.01 m/s^2^; one-sample *t*-test, *P* = 0.11) resulting in a significant difference between the groups (two-sample *t*-test, *P* = 0.05).

#### Starting and walking

The cortical activity during starting and walking are presented in Supplementary Fig. 4. Overall, we observed first a decrease in activity in M1 and PMC when starting to walk [M1, −0.15 (−0.28 −0.03); PMC, −0.17 (−0.29 −0.05)], which was followed by an increase in activity in M1 during walking [M1, 0.22 (0.08 0.36)]. None of the 95% CrI of the group effects excluded zero. The Parkinson’s disease group accelerated slower than the HC group in the first 3 s after the start signal (Parkinson’s disease, 0.16 m/s^2^; HC, 0.26 m/s^2^; two-sample *t*-test, *P* = 0.003; Supplementary Fig. 4C).

#### Correlations with freezing severity

We found a positive correlation between PFC activity during a normal doorway passage and the % time frozen due to doorway freezing in the Parkinson’s disease group, also after correcting for MDS-UPDRS III scores [mean (95% CrI) 0.28 (0.08 0.49)]. PFC activity during stopping was correlated with the MDS-UPDRS III score [0.17 (0.00 0.33)]. No other correlations were found in the other ROIs, nor for the other normal gait events (turning, standing, starting and walking).

### Freezing versus stopping versus successful gait events

**Fig. 5:**
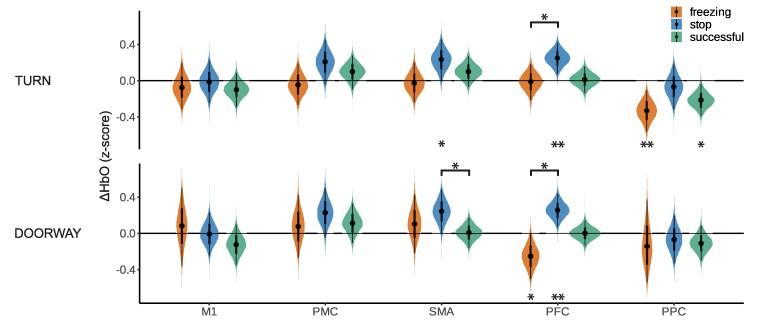
**Freezing of gait versus stopping versus successful gait events.** Posterior probabilities of the estimated average ΔHbO responses for each ROI during freezing of gait (‘freezing’, orange), stopping (‘stop’, blue) and successful gait events (‘successful’, green) as calculated by the Bayesian hierarchical model (turn, N_freezing_ = 17, N_stop_ = 21, N_successful_ = 16; doorway, N_freezing_ = 9, N_stop_ = 21, N_successful_ = 22). The stars underneath the plots indicate if the posterior probability of the estimated ΔHbO response is different from the stars above the violin plots indicate if the posterior probability of the estimated ΔHbO response differs between the groups (* = 95% CrI excluding zero; ** = 99% CrI excluding zero). The cortical activity map during freezing is displayed in Box 1. For abbreviations, see Fig. 3.

When comparing cortical activity during freezing with voluntary stops and successful gait events, we observed substantial differences in the PFC between freezing and stopping: the PFC activity was lower during freezing than during stopping, both for freezing elicited by turning and for freezing elicited by doorway passage [turn freezing, −0.26 (−0.51 0.00); doorway freezing, −0.50 (−0.82 −0.22); Fig. 5]. Additionally, PFC activity during doorway freezing was decreased compared to baseline [−0.25 (−0.51 0.00)]. The other ROIs did not show differences compared to the freezing condition with 95% CrI excluding zero.

## Discussion

We used fNIRS to investigate cortical activity related to freezing of gait during free ambulation. We compared cortical activity of people with freezing to age-matched healthy controls and—in line with our hypothesis—we observed higher and more widespread activity in the premotor areas during turning and gait termination. Similar effects were observed during doorway passing, but only when considering a subgroup that effectively experienced doorway freezing in the experiment. Interestingly, the cortical activity during turning and doorway passing was correlated with the cortical activity during stopping. Although this suggests that freezing is related to stopping, freezing was different from stopping by having ‘lower’ PFC activity. Note that this lower PFC activity is opposite from what was hypothesized from the previous literature. Taken together, we postulate that freezing might result from an imbalance between the SMA and the PFC within the stopping network. We furthermore highlight the advantage of fNIRS to investigate gait during free ambulation, but note the need for careful correction of confounds occurring during walking (Box 1).

### The premotor areas are consistently related to freezing pathophysiology

The increased premotor area activity in the Parkinson’s disease group during turning and doorway passage is in line with previous fMRI studies^22,46,47^ and one previous fNIRS study.^92^ Even while there were differences between the fMRI studies’ findings of increased or decreased activity, they all identified the SMA as a significant locus of action. The fNIRS study reported increased SMA and PMC activities during turning in people with Parkinson’s disease compared to controls, but not in freezers.^92^ However, this study did not specify whether freezing episodes were excluded from analysis. Additionally, we observed widespread SMA activity in the Parkinson’s disease group during gait termination. This SMA activity was still present 7–10 s after stopping, suggesting prolonged activation of this area after having come to a standstill.

Taken together, these results provide support for the role of the SMA in the pathophysiology of freezing. It has been hypothesized that excessive SMA recruitment leads to disrupted communication with the subthalamic nucleus (STN), leading to involuntary activation of the hyperdirect pathway, hence putting a brake on ongoing or initiating movements.^18,46,47^ We speculate that the prolonged SMA activity after coming to a stop might explain why people with Parkinson's disease have greater difficulty initiating gait, as they would require to first deactivate the hyperdirect pathway before taking the first step.^93^ Alternatively, the increased SMA activity during standing relative to the walking baseline might also represent a decreased SMA activity during walking instead.^24,94-96^

Although we did find correlations of PFC activity with freezing severity during doorway passing and with the MDS-UPDRS motor scores for successful stopping, we did not find correlations of the SMA/PMC activity with freezing severity or MDS-UPDRS. It is therefore not entirely clear whether the observed premotor overactivity relates to freezing of gait specifically, or to Parkinson's disease in general, although the subgroup analysis of the doorway freezers suggests a more freezing-specific relationship. This study was specifically designed to include persons that reported to have freezing at least daily to increase chances to capture cortical activity during actual freezing episodes. Nevertheless, future studies could also include participants without freezing, so better conclusions can be made about the nature of the premotor area overactivity.

### Freezing might be related to stopping

Stopping induced activation of the SMA and PFC in the PD group, and *post hoc* analysis revealed correlations between the cortical activation patterns during stopping and turning, and stopping and doorway passage, for both study groups. The SMA and PFC, or more specifically, the SMA and the inferior frontal gyrus (IFG), are well-known for their role in inhibiting actions.^97^ They are considered to be part of the stopping network^74,98,99^ and have previously been associated with imagined termination of gait.^75^ From the estimated channel positions (Supplementary Table 3), we can infer that our channels mainly covered this gyrus of the PFC. The correlations suggest that turning and doorway passage activate a ‘preparatory’ stopping network, hence might explain why these actions are prone to elicit freezing (note that haemodynamic responses are delayed by approximately 6 s). Such a preparatory stopping network, often referred to as the ‘proactive inhibitory control network’ and also including the SMA and IFG, has been reported to facilitate stopping behaviour in case a quick brake is required.^74,98,100^ For example, when people walk towards a door, they may not be certain about what they will encounter behind the door, or they might be unsure whether they would be able to pass through without any collisions. In this study specifically, the alternation between stopping in front of the door/in the square and continued walking might have played an additional factor.

Similar to the proactive stopping network, the ‘hold-your-horses’ principle postulates that a stopping network is activated in case multiple motor programmes are competing with each other to withhold the motor response until a final decision is made.^101^ This principle has previously been proposed as a possible explanation for freezing.^34^ However, following this principle, we would not expect the HC group to show similar correlations between doorway passing and stopping, as they have no reason to exhibit conflicting motor programmes. Another explanation for the similarity in cortical activity between turning/doorways and stopping is that both patterns represent a switch in motor programming, rather than the activation of a stopping network.

Although both groups showed correlations with their respective stopping activation patterns during doorway passage, only the Parkinson’s disease group slowed down at the door, while the HC group maintained a constant speed. This slowing of Parkinson’s disease patients is in line with previous behavioural studies showing that freezers decrease their walking speed at narrow doorways.^102-104^ Consequently, the HC group seems to be more successful in suppressing the stopping programme, or alternatively, the Parkinson’s disease group is more sensitive to the stopping signals.

### Freezing is not the same as stopping

The observation that freezing might be related to stopping prompts the question whether freezing and stopping share a similar mechanism. When directly comparing freezing to stopping, the PFC (IFG) showed distinct activity, with an increase in activity during stopping, but a decrease (doorway freezing) or no change (turning freezing) in activity during freezing. Moreover, the IFG activity during stopping correlated with the MDS-UPDRS scores (higher activity for worse Parkinson's disease symptoms). Based on this observation, we suggest that IFG activation is crucial for voluntary stopping strategies. The precise role of the IFG and SMA within the stopping network is still under debate, but IFG is thought to be critical for attentional monitoring and stop-signal detection, while SMA is thought to be a direct communicator with the STN establishing the hyperdirect pathway.^74,105^

An important note is that the relatively low PFC (IFG) activity during freezing is opposite of what has been reported previously and thus also opposite from what we had hypothesized. A previous fNIRS study measuring PFC activity during turn freezing reported increased PFC activity during freezing episodes.^27^ This study, however, did not correct for potential systemic confounds like changes in heart rate and blood pressure. Indeed, when we did not apply a short-channel correction to our data, we observed similar increases of fNIRS signals globally over the whole scalp during freezing (Box 1) and in all the short channels, measuring only superficial scalp haemodynamics. Moreover, the fNIRS signals during freezing and stopping resembled the time courses of heart rate data that have been reported previously,^106,107^ suggesting a systemic origin of the previously observed increased fNIRS activity. Another fMRI study from Shine and colleagues^34^ using virtual reality and foot pedalling reported increased blood oxygenation in the IFG during abnormally long foot pedalling latencies—which were considered the clinical correlate for a freezing episode. However, the question remains how well these motor arrests correspond to freezing during free ambulation. Another possible explanation for the discrepancy between our study and the study from Shine and colleagues is that the respective region of interest did not correspond. Nevertheless, when comparing the MNI coordinates of both studies, the highlighted area from that study was close to our optode array (Supplementary Fig. 7).

### Freezing: a paroxysmal imbalance between the supplementary motor area and inferior frontal gyrus within the stopping network?

Taken together, we propose to extend the hypothesis of excessive SMA activity leading to freezing, by introducing an extra role of the IFG within the stopping network. Based on our results—and supported by other studies—we hypothesize that perfect coordination between the SMA and the IFG is essential to effectively transmit braking signals to the STN.^105^ We compare this to the required cooperation between the switch and clutch of a (non-automatic, stick shift) car. When switching gears, this needs to be accompanied by careful control of the clutch. If not, the car stalls. Hence, we could consider freezing as a failure of compensation by the IFG. The proposed mechanism might also explain the negative influence of dual-tasking on the occurrence of freezing, as a concomitant task might interfere with the attentional capacity of the IFG.^28,29,108,109^

Evidently, the proposed model is simplistic and should be challenged by future research. For example, perturbing the SMA and/or IFG with non-invasive transcranial stimulation could shed light on the differential roles of both areas in freezing pathophysiology. Moreover, modern deep brain stimulation devices, which are now able to record STN activity, could help to elucidate the role of the STN within the stopping network, especially when combined with cortical measurements of the SMA/IFG.^110,111^ As a future application, a closed-loop deep brain stimulation system including SMA/IFG measurement could intervene during an upcoming or ongoing freezing episode by briefly suppressing the STN.^112^

## Conclusion

This study provides evidence for the role of the SMA in freezing of gait pathophysiology by investigating cortical activity during free ambulation. We extend upon this theory by suggesting that freezing arises from a paroxysmal imbalance between the SMA and the IFG. Furthermore, we showed that IFG activity is lower during freezing than during stopping, which is opposite to previous findings,^27^ but attributable to an improved correction for movement confounds in our study. Lastly, we provide recommendations for future fNIRS gait studies on how to correct for walking-induced artefacts (Box 1).

## Supplementary Material

fcae259_Supplementary_Data

## Data Availability

The raw data of all participants (excluding one that did not agree with data sharing) are organized in Brain Imaging Data Structure (BIDS) format^89-91^ and are openly shared on the Donders Repository as ‘Cortical activity measured with fNIRS related to freezing of gait in Parkinson's disease’ (https://doi.org/10.34973/k7ce-6n58). The underlying code for this study is available via https://github.com/helenacockx/freezing_stopping_fNIRS.
