# Supplementary material for: Freezing of gait in Parkinson's disease is related to imbalanced stopping–related cortical activity
Source: Brain Commun. 2024 Aug 2;6(5):fcae259. doi: 10.1093/braincomms/fcae259 (PMC11369826; doi:10.1093/braincomms/fcae259)
Supplement: fcae259_Supplementary_Data [file fcae259_supplementary_data.zip › Original_submission_manuscript.pdf]

OXFORD UNIVERSITY PRESS | Brain Communications

# Freezing of gait in Parkinson's disease is related to imbalanced stopping-related cortical activity



| | |
|---|---|
| Journal: | *Brain Communications* |
| Manuscript ID | BRAINCOM-2023-473 |
| Manuscript Type: | Original Article |
| Date Submitted by the Author: | 20-Oct-2023 |





| | |
|---|---|
| Complete List of Authors: | Cockx, Helena; Radboud University, Donders Center of Neuroscience; Department of Neurobiology; Radboudumc, Donders Institute for Brain, Cognition and Behaviour; Department of Neurology; Center of Expertise for Parkinson and Movement Disorders<br>Oostenveld, Robert; Radboud University Donders Institute for Brain Cognition and Behaviour, Donders Center for Cognitive Neuroimaging; Karolinska Institutet, NatMEG<br>Flórez R., Yuli A.; Radboud University, Donders Center for Neuroscience; Department of Neurobiology; GGZ Mondriaan<br>Bloem, Bastiaan; Radboudumc, Donders Institute for Brain, Cognition and Behaviour; Department of Neurology; Center of Expertise for Parkinson and Movement Disorders<br>Cameron, Ian G.M.; Radboud University, Donders Center of Neuroscience; Department of Neurobiology; University of Twente, Faculty of Electrical Engineering, Mathematics and Computer Science (EEMCS); Biomedical Signals and Systems Group; OnePlanet Research Center<br>van Wezel, Richard J.A.; Radboud University, Donders Center of Neuroscience; Department of Neurobiology; University of Twente, Faculty of Electrical Engineering, Mathematics and Computer Science (EEMCS); Biomedical Signals and Systems Group |





| | |
|---|---|
| Keywords: | freezing of gait, Parkinson's disease, functional near-infrared spectroscopy (fNIRS), cortical activity, stopping |



SCHOLARONE™
Manuscripts

# Freezing of gait in Parkinson's disease is related to imbalanced stopping-related cortical activity

Helena M. Cockx,*[1,2] Robert Oostenveld,[3,4] Yuli A. Flórez R,[1,5] Bastiaan R. Bloem,[2] Ian G.M. Cameron,[1,6,7] Richard J.A. van Wezel[1,6]

**Author affiliations:**

1 Department of Neurobiology, Faculty of Science, Donders Institute for Brain, Cognition and Behaviour, Radboud University, 6525AJ Nijmegen, The Netherlands

2 Department of Neurology, Center of Expertise for Parkinson and Movement Disorders, Donders Institute for Brain, Cognition and Behaviour, Radboud University Medical Center, 6525GC Nijmegen, The Netherlands

3 Donders Center for Cognitive Neuroimaging, Donders Institute for Brain, Cognition and Behaviour, Radboud University, 6525EN Nijmegen, The Netherlands

4 NatMEG, Karolinska Institutet, 17177 Stockholm, Sweden

5 GGZ Mondriaan, 6401CX Heerlen, The Netherlands

6 Biomedical Signals and Systems Group, Faculty of Electrical Engineering, Mathematics and Computer Science (EEMCS), University of Twente, 7522NB Enschede, The Netherlands

7 OnePlanet Research Center, 6525EC Nijmegen, The Netherlands

***Correspondence to:**

Helena Cockx, M.D.

Radboud University Medical Center

Renier Postlaan 4

6525GC, Nijmegen

the Netherlands

helena.cockx@radboudumc.nl

For Review Only

# Abstract

Freezing of gait, characterized by involuntary interruptions of walking, is a debilitating motor symptom of Parkinson's disease that restricts people's autonomy. Previous brain imaging studies investigating the mechanisms underlying freezing were restricted to scan people in supine positions and yielded conflicting theories regarding the role of the supplementary motor area (SMA) and other cortical regions. We used functional near-infrared spectroscopy (fNIRS), to investigate cortical hemodynamics related to freezing in freely moving people.

We measured fNIRS activity over multiple motor-related cortical areas in 23 persons with Parkinson's disease who experienced daily freezing ("freezers") and 22 age-matched controls during freezing-provoking tasks including turning and doorway passing, voluntary stops, and actual freezing. Crucially, we corrected fNIRS signals for confounds of walking. We first compared cortical activity between freezers and controls during freezing-provoking tasks without freezing (i.e., turning and doorway passing) and during stops. Secondly, within the freezers, we compared cortical activity between freezing, stopping, and freezing-provoking tasks without freezing.

First, we show that turning and doorway passing (without freezing) resemble cortical activity during stopping in both groups involving activation of the SMA and prefrontal cortex (PFC), areas known for their role in inhibiting actions. During these freezing-provoking tasks, the freezers displayed higher activity in the premotor areas than controls. Secondly, we show that, during actual freezing events, activity in the PFC was lower than during voluntary stopping.

The cortical relation between the freezing-provoking tasks (turning and doorway passing) and stopping may explain their susceptibility to trigger freezing by activating a stopping mechanism. Besides, the stopping-related activity of the SMA and PFC seems to be out of balance in freezers. In this paper, we postulate that freezing results from a paroxysmal imbalance between the SMA and PFC, thereby extending upon the current role of the SMA in freezing pathophysiology.


For Review Only

**Running title**: Freezing & imbalanced stopping activity

**Keywords:** freezing of gait; Parkinson's disease; functional near-infrared spectroscopy (fNIRS); cortical activity; stopping

**Abbreviations:** CrI = credibility interval; fNIRS = functional near-infrared spectroscopy; GPI = globus pallidus internus; HbO = oxygenated hemoglobin; HbR = deoxygenated hemoglobin; HC = healthy control; IFG = inferior frontal gyrus; M1 = primary motor cortex; MDS-UPDRS III = Movement Disorder Society's Unified Parkinson's Disease Rating Scale part III; MNI = Montreal Neural Institute; PD = Parkinson's disease; PFC = prefrontal cortex; PMC = premotor cortex; PPC = posterior parietal cortex; ROI = region of interest; SMA = supplementary motor area; SNR = substantia nigra pars reticularis; STN = subthalamic nucleus

For Review Only

# Introduction

Normally, we initiate and stop walking as voluntary actions. However, 30-60% of the people with Parkinson's disease experience sudden interruptions of gait that are not voluntary.[1-3] This is called *freezing of gait* and is considered one of the most debilitating symptoms of Parkinson's disease,[4] leading to reduced mobility, falls, fear of falling, and social exclusion.[5-9] Freezing occurs frequently during situations where motor programs must be adapted, such as during turning, walking through doorways, approaching a destination, and starting to walk.[4,10] Standard dopamine-replacement therapy is rarely sufficient to treat freezing, and the development of novel therapies is hampered by our limited knowledge of the underlying brain mechanisms.[11-14] The current study aims to get to a better understanding of the cortical mechanisms underlying freezing of gait when triggered by changes in motor programs.

Previous research converges on the hypothesis that various triggers of freezing activate an as-of-yet-unknown cortical network, eventually activating a common pathway in the basal ganglia and brainstem nuclei.[10,15,16] Within this final pathway, the globus pallidus internus (GPi) and the substantia nigra pars reticularis (SNr) transiently increase their inhibitory output, leading to decreased activity in gait-controlling brainstem nuclei and eventually uncoordinated firing of the central pattern generators. Although the excessive GPi-SNr output seems to play a key role, there is evidence that the actual problem of freezing lies higher up in the cerebral cortex and the cortico-basal connections, which are responsible for a flexible adaptation of gait.[12,15] Several theories have been proposed to explain this cortical mechanism (for a review, see[16]). For instance, excessive activation of the supplementary motor area may temporarily recruit the hyperdirect pathway, activating the GPi-SNr via the subthalamic nucleus.[17,18] Alternatively, conflicting cortical processes in motor, cognitive, and limbic cortical areas could transiently overwhelm the striatum, thereby losing its inhibitory control over the GPi.[19]

For Review Only

Many of the theories on freezing of gait pathophysiology are based on neuroimaging methodologies that only allowed one to study the neural correlates of gait indirectly while participants were lying supine in confined spaces. For example, functional MRI studies rely on the imagination of gait or the mimicking of gait with foot pedals while navigating through a virtual environment.[20-22] PET scanners can only study gait on a coarse time scale as they are restricted to scan the brain after completion of a gait task.[23,24] Although these studies have improved our understanding of freezing of gait mechanisms, the question remains on how to translate these findings to “real” gait.

In the present study, we use functional near-infrared spectroscopy (fNIRS) to measure cortical activity related to freezing of gait in Parkinson’s disease during free ambulation. fNIRS is a wearable neuroimaging technique that – similar to functional MRI – is sensitive to changes in local hemodynamics.[25,26] It measures changes in oxygenated (HbO) and deoxygenated (HbR) hemoglobin by transmitting infrared light through the cortex at prespecified locations. Unlike previous studies that mainly focused on the prefrontal cortex,[27-30] we assessed fNIRS activity over multiple cortical areas which have previously been associated with freezing: the premotor cortex (PMC), the supplementary motor area (SMA), the prefrontal cortex (PFC), the posterior parietal cortex (PPC), and the primary motor cortex (M1).[16]

For Review Only

The goal of this work was two-fold. We first investigated whether people with Parkinson’s who experience freezing (“freezers”) recruited different cortical areas than age-matched controls when voluntary stopping and when successfully (i.e., without freezing) performing gait tasks involving changes of motor programs: making 180° turns, passing through a doorway, and starting to walk. Second, within the group of freezers, we examined which cortical areas showed freezing-related activity and how their activity differed from stops and from the same gait events without freezing (e.g., successful turns and doorway passages).

# Materials and methods

## Participants

We recruited participants (age >18 years) with the help of ParkinsonNEXT (https://www.parkinsonnext.nl), a Dutch online platform connecting people with Parkinson's to researchers. We included participants who were diagnosed with idiopathic Parkinson's disease according to accepted international standards[31,32] and who subjectively reported freezing at least once a day. We did not differentiate between OFF or ON state freezing as an inclusion criterium. We encouraged the participants with Parkinson's disease (PD) to bring their partners, relatives, or friends to serve as healthy control. The healthy controls (HC) were matched at the group level for age and gender to the PD group. We used the following exclusion criteria: comorbidities causing severe gait impairments; comorbidities that could interfere with the fNIRS recording such as structural brain lesions or previous brain surgery (including deep brain stimulation); and inability to comply with the protocol including severe cognitive impairment as judged by a clinician (HMC). Absence of objective freezing during the study protocol was not an exclusion criterium. In total, three of the 25 PD participants did not show freezing during the protocol. Two of them were later validated as being freezers based on home-made videos.

The participants with PD performed all procedures in the OFF state, following at least 12 hours overnight withdrawal of anti-Parkinson medication.

A trained clinician (HMC) assessed motoric symptom severity by the Movement Disorders Society Unified Parkinson's Disease Rating Scale part III (MDS-UPDRS III)[33] at the start of the lab visit (OFF medication). The other questionnaires and tests were completed after the gait tasks: the new freezing of gait questionnaire,[34] the Montreal Cognitive Assessment,[35] the Trail Making Test part A and B,[36] the Hospital Anxiety and Depression Scale,[37,38] and a

For Review Only

supplementary set of questions asked for feelings of anxiety and insecurity during the different parts of the walking task (e.g., "*I felt insecure/anxious when walking through the door.*").

In total, we recruited 24 people for the HC group and 25 people for the PD group following previous recommendations for fNIRS gait studies.[39,40] The data from 22 HC and 23 PD participants were included in the final analysis. Reasons to exclude the data from the other four participants were as follows: two HC participants were excluded because of technical issues with the motion capture system; one person with PD was not able to complete the protocol due to fatigue; one person with PD showed a poor quality of the fNIRS data (>50 % of the channels with poor signal quality, see later).

The medical ethics committee Arnhem-Nijmegen approved of the study (NL70915.091.19). All procedures were conducted in the respect of the Declaration of Helsinki and the Medical Research Involving Human Subjects Act (WMO). Data handling followed the General Data Protection Regulation (EU GDPR). All participants provided written informed consent before participating. They had the possibility to opt-in for data sharing of their de-identified research data.

For Review Only

## Gait task

The gait task involved walking at a comfortable pace in a long corridor (Fig. 1). Halfway through the corridor, the participants passed through a narrow doorway frame of 60 cm wide. At the ends of the corridor, they made 180° turns in a 50 cm wide square taped on the floor. After every passage of two doors and two turns (i.e., every 2.5 corridor lengths) they were instructed to stop in front of the door, or in the square for 30 s. The regularity of these intervals was introduced to minimize the unpredictability of stops. Halfway through the 30 s, they were instructed to make one step through the door or to turn around for 180 degrees, and then resume walking. All participants practiced the walking paradigm. The distance between the door and

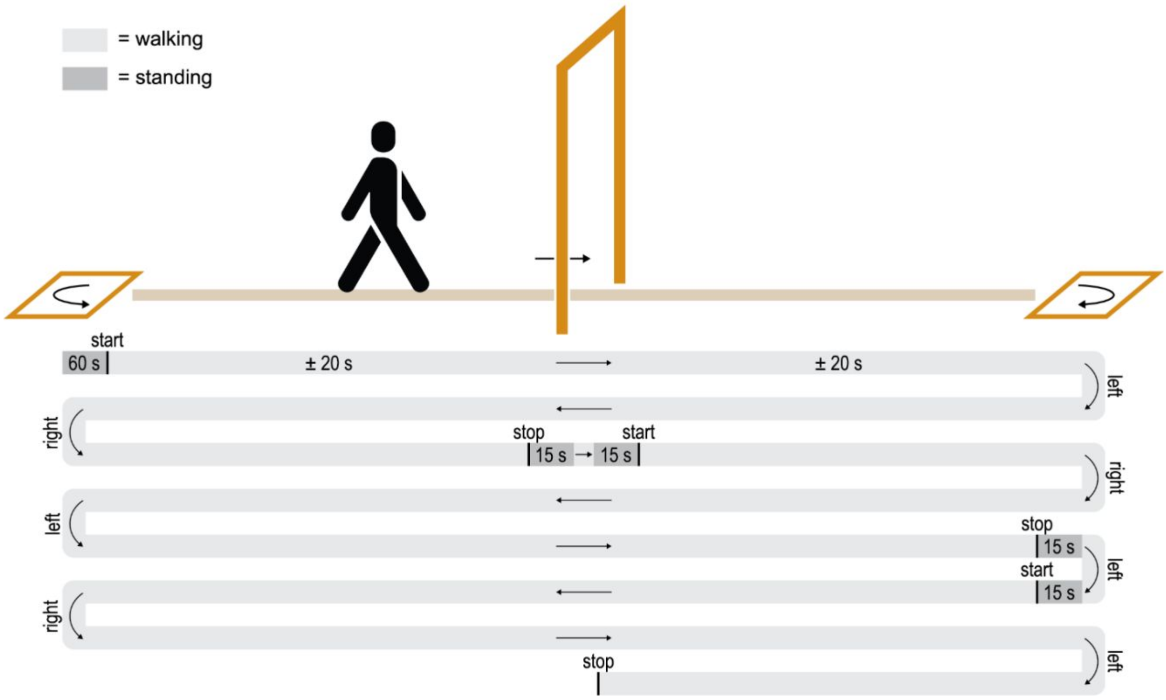


Fig. 1: Gait task. Example of one run (+- 6.5 min) of the gait task. Participants walk up and down a corridor, halfway passing through a narrow doorway frame (60 cm wide), and at the ends making 180° turns in a square taped on the floor (50 cm wide). The distance between the doorway and the square was set at an individualized walking distance of approximately 20 s. Each 2.5 corridor length, participants were instructed to stop in front of the door or in the square, to make one step through the door or turn around after 15 s, and then to resume walking after another 15 s. The direction of the turns was alternated between each side of the corridor and this direction was switched after each instructed stop. Each participant completed approximately four runs in total.

the turns was individualized based on the walking speed during the practice trial to achieve a walking duration of approximately 20 s. This ensured a stable walking baseline of fNIRS activity between two gait events (e.g., a doorway passage and a turn).[39]

To minimize cognitive load, instructions to stop or keep walking, or to turn left or right, were given approximately two meters (i.e., ± 2 s) before encountering the door or the square. The direction of the turn, i.e., leftwards or rightwards, was alternated between the squares. To guarantee an equal number of turning directions at each side of the corridor, we switched the turning directions after each instructed stop (see Fig. 1). Participants were discouraged to use any compensation strategy to improve their walking and were instructed not to talk or make extra movements such as scratching their head during the gait tasks.

In total, the participants completed four identical runs of approximately 6.5 minutes each, with standing upright for 60 s at the beginning of each run to avoid changes in orthostatic blood pressure that could confound the fNIRS recordings. Between each run, they could rest as long as needed. Participants walked unaided but were always accompanied by a researcher to prevent them from falling.

# Materials

## fNIRS

Two continuous-wave fNIRS devices (Brite24, Artinis Medical Systems) were combined into one cap. Each device consisted of eight photo-diode detectors sampling at a 50 Hz rate and sixteen light-emitting diode emitters with nominal wavelengths of 760 and 850 nm. These detectors and emitters ("optodes") were placed in a neoprene cap (headcap with print, size M or L, Artinis Medical Systems) with custom-made holes according to the layout as shown in Fig. 2. In total, there were 32 long channels with an interoptode distance of 30 mm and 16 short channels with an interoptode distances of 10 mm. Short channels only penetrate the superficial layers of the scalp and are intended

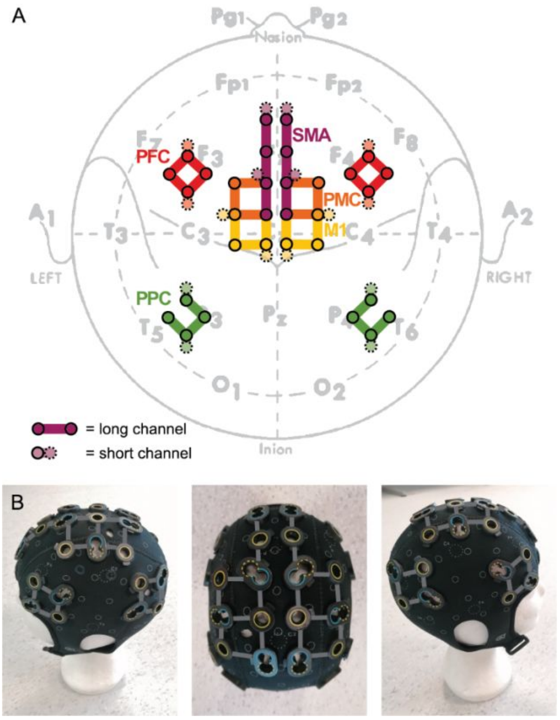


Fig. 2: fNIRS cap layout. (A) Schematic representation of the cap layout indicating the positions of the long channels (30 mm interoptode distance) and short channels (10 mm interoptode distance) relative to the 10-20 EEG reference system and the main sulci. The recorded channel positions are presented in supplementary Fig. 7 and supplementary Table 3. (B) Images of the cap with the detectors in blue and the sources in yellow (dashed line for low output source). (M1 = primary motor cortex (yellow); PMC = premotor cortex (orange); SMA = supplementary motor area (purple); PFC = prefrontal cortex (red); PPC = posterior parietal cortex (green)).

to record and correct for physiological systemic artifacts such as changes in heart rate, blood pressure, and breathing.[41,42]

The cap was designed to optimally cover the areas previously associated with freezing[16,43-47], however, at the same time avoiding interference between the optodes of the two devices that would happen if placed too close to each other. The cap was placed according to the 10-20 electrode placement scheme with Cz as the midpoint between the nasion and inion, and between the left and right pre-auricular point. We used an optical 3D scanner (Structure Sensor, Occipital) to record the optode positions on each participant's head.[48] We calculated the Montreal Neurological Institute (MNI) coordinates of each channel and estimated their underlying brain regions defined by automated anatomical labelling[49] (see supplementary material).

The fNIRS devices connected wirelessly via Bluetooth to a laptop running the Artinis recording software (OxySoft 3.2.70). The quality of the data was checked prior to recording and during the breaks in between the runs.

## Motion capture

Seventeen inertial measurement units (MVN Awinda system, Xsens) were attached to the body with Velcro straps. The data, sampled at 60 Hz, was wirelessly transmitted to a laptop running the Xsens recording software (MVN Awinda, version 2020.0.1). This software synchronizes the 17 motion sensors and performs offline processing, resulting in full-body movement data, including the position and orientation of all body parts, as well as the acceleration and angular velocity of each sensor.

For Review Only

## Video

Two video cameras (Canon Legria HFG26, sampling rate 25 Hz) were placed at each end of the corridor, approximately two meters behind the turning squares and directed towards the doorway. A third camera was mounted on a wheelchair following the participant during the gait tasks. All cameras targeted the legs and feet of the participant, to avoid unnecessary participant identification.

## Synchronization

We synchronized the fNIRS, movement, and video data offline using sync events that were sent simultaneously to the different recording devices. For fNIRS, these sync events consisted of Lab Streaming Layer markers (LSL) (https://github.com/sccn/labstreaminglayer, *accessed on*: 3-4-2023) which were registered by the fNIRS recording software. For the movement data, simultaneous with the LSL markers we created TTL pulses which were recorded by the Xsens hardware. For the video data, the sync events consisted of audio beeps (inaudible to the participant) that were recorded along with the video via the external microphone input. Given that the experimental setup spanned a corridor of about 50 m long, we sent out the sync events using ZeroMQ (https://github.com/zeromq, *accessed on:* 3-4-2023) over a local network connected to the different recording devices either wirelessly (video) or via ethernet cable (fNIRS, Xsens).

For Review Only

# fNIRS Data Preprocessing

The fNIRS data was loaded into MATLAB and preprocessed using a combination of FieldTrip,[50] Homer3,[51] and custom functions, minimally following previous recommendations for fNIRS studies.[26,39] We used pilot data (from two young adult subjects) to construct the preprocessing pipeline and to define preprocessing parameters (e.g., for motion artifact

correction) and adjusted those parameters based on the first three to five participants of our dataset.

The fNIRS data was first resampled from 50 to 60 Hz to match the movement data. For each run, we removed channels that were too low in quality, defined as a signal quality index of less than two for more than half of the time the participant was standing still.[52] On average, 15% of the long channels (4-5 channels per participant), and 16% of the short channels (2-3 channels per participant) were removed. Motion artifacts were corrected with the combination of a movement artifact correction algorithm using spline interpolation (*stdv threshold* = 65; *amplitude threshold* = 0.05; *tMotion* = 0.5; *tMask* = 1)[53] and a wavelet correction (*IQR* = 0.8).[54-57] Optical densities were converted to hemoglobin concentration changes based on the modified Beer-Lambert-Law with a differential pathlength factor adapted to age and wavelength.[58]

We removed slower confounding factors, such as systemic artifacts induced by physical activity[59,60] and head movements,[61] by regressing out short channel data and movement data of the head (see also Box 1).[61-63] We first applied a 0.5 Hz low pass filter (3rd order Butterworth) to both the fNIRS and movement data to remove faster noise components (e.g., heartbeats, footsteps). For regression of the short channels, we z-transformed the signals and used the first eight components of a principal component analysis (both HbO and HbR), representing >90% of the variation in these channels. For regression of the movement data, we used the z-transformed acceleration of the head, the angular acceleration of the head, and the orientation of the head relative to the neck, as we observed that participants looked down while performing the gait tasks (Suppl. Fig. 1). For each long channel, we performed an ordinary least square regression with the long channel as the dependent variable and the short channels and movement data as regressors. The residuals of the regression analysis were used for further analysis.

For Review Only

Subsequently, we applied a 0.01 Hz high pass ($2^{nd}$ order Butterworth) and a 0.1 Hz low pass ($6^{th}$ order Butterworth) filter to remove remaining noise components. The cut-off values of these filters were chosen by checking the power spectra plots of each participant individually to optimally remove slow drifts and Mayer waves while retaining the relevant task frequency.[64]

Finally, we z-transformed each fNIRS channel to be able to average over multiple channels belonging to the same cortical region, obtaining normally distributed data. We calculated z-scores by subtracting the mean HbO or HbR values of each channel and each run, and by dividing those by their standard deviation.

# Gait Events

Freezing of gait events were annotated on video by two independent trained raters (HMC and YAFR) in the ELAN software (https://archive.mpi.nl/tla/elan, *accessed on:* 3-4-2023*)*, as described by Gilat.[65] The annotations of both raters were subsequently compared and combined using FOGtool[66] (*tolerance* = 2 s; *correction* = include). The positive agreement between the raters was 0.86 and the negative agreement 0.98, with a prevalence index of -0.74. Remaining non-overlapping annotations were discussed until consensus was reached. Freezing events were only considered for analysis when not preceded by another freezing event within 10 s. In total, 550 freezing events reached consensus, of which 104 were excluded.

We defined successful gait events as turns, doorway passages, starts, or stops without any freezing event 10 s before or 10 s after the event. The successful gait events were defined by the movement data and were checked for correctness by looking at the videos. A detailed description of this process can be found in the supplementary material. In total, 1231 normal gait events for the PD group and 1596 normal gait events for the HC group could be used for final analysis.

For Review Only

## Statistics

We analyzed the fNIRS data with Bayesian hierarchical models in Rstudio (RStudio 2022.07.2; Rstudio, Inc., Boston, MA) using the brms package (version 2.18.0).[67] Two types of statistical models were built: one to compare the cortical activity of the PD group to the HC group for the various gait events when no freezing occurred (e.g., stop in PD vs HC; successful turn in PD vs HC); and one to compare the cortical activity during a freezing event to a voluntary stop and to a successful event of the same type as the freezing event (e.g., turning freeze vs stop vs successful turn). Note that each PD participant contributed a various number of freezing events and successful gait events, yielding an imbalanced data design. Hierarchical models account for imbalanced data by *shrinkage,* meaning that the data from participants contributing less or more variable data are pulled towards the group mean.[68,69] Moreover, hierarchical models estimate all effects simultaneously. This implies that we can inspect the effects of the studied factors within a model directly, for example, when looking at the cortical activity in the PD and HC groups individually, thereby increasing statistical sensitivity and removing the need to correct for multiple comparisons.[68,70]

The main outcome variable was the mean HbO within a region of interest (ROI) during the different event types. For each channel, we calculated the average HbO from 0 to 3 s after the event onset (i.e., onset of stop, turn, doorway, start, or freezing). For stopping and starting, we additionally calculated the average HbO from 7 to 10 s after the stopping and starting events to assess cortical activity during standing and walking. All values were baseline-corrected by subtracting the average HbO from 10 to 5 s before the event onset. This baseline was chosen to contain a stable reference signal during walking (e.g., turns, doors, stops) or standing (e.g., starts), yet before the instructions were given to stop, start, or keep walking. Subsequently, we averaged the HbO values of the channels belonging to the same ROI corresponding to the layout of Fig. 2. Time courses of HbO and HbR values were visually checked to contain reliable

For Review Only

hemodynamic responses (Supplementary Fig. 6), but no statistical analysis was performed on the HbR values.

The first type of model included a fixed intercept, representing the global activity during the gait event compared to baseline, and a fixed effect for group (sum-contrast-coded), representing the difference in activity between the PD and HC group. A random intercept for participant accounted for individual differences in cortical activity. Left and right turns were pooled as they did not display significant direction effects, nor interaction effects with the hemisphere. The second type of model included a fixed intercept (activity-vs-baseline), a fixed effect for condition (freeze vs stop vs successful gait event; sum-contrast-coded), and a random intercept for participant and a random slope for condition varying over participants. We only computed models for freezing triggered by turns or doorways that occurred during walking, as we did not have enough data for the other freezing types. We investigated differences in timing between the onset of the freezing events and the onset of the stop or successful gait events, to assess whether these events could be compared reliably. The turning freezing occurred mostly at the start of the turn (median [IQR]: 11% [0 27%]) and the doorway freezing approximately 0.3 m before the door (median [IQR]: 0.27 [0.52 0.14]) (Suppl. Fig. 2). Considering the slowness of the hemodynamic signal, we concluded that the difference in timing was negligible. We executed a model for each type of event (model type 1) or freezing type (model type 2), and for each ROI separately. Additionally, to visualize the results on cortical activity maps, we calculated a model for each channel.

To assess whether fNIRS activity during the successful gait events in the PD group was specifically related to freezing, we exploratively fitted a third type of model for this group. This model included a fixed intercept, a fixed effect for % time frozen (specifically triggered by the studied gait event), a fixed effect for MDS-UPDRS motor score, and a random intercept per

For Review Only

participant. The % time frozen and MDS-UPDRS scores were centered and standardized before model fitting.

Posterior probabilities of the model parameters were estimated with Markov chain Monte Carlo sampling, starting from flat priors. Details on the sampling procedure, the priors, and the checks that were performed are provided in the supplementary material. We visualize the probability density functions of the estimated effects and report the 95% credibility intervals (CrI), calculated from quantiles of the probability functions. We consider the 95% CrI as the probability threshold to make claims about the sign of the fNIRS activity (i.e., being higher or lower than its control condition).[71-73] Note that posterior probabilities from Bayesian models directly express the belief of the underlying studied effects, given the data. They are therefore more intuitive and less prone to type I errors than classical p-values from frequentist statistics, which are calculated based on hypothetical replications of the experiment.[74,75]

# Data availability

The raw data of all participants (excluding one that did not agree with data sharing) are organized in Brain Imaging Data Structure (BIDS) format[76,77] and are openly shared on the Donders Repository as "Cortical activity measured with fNIRS related to freezing of gait in Parkinson's disease" (https://doi.org/10.34973/k7ce-6n58). [Note to the editor and reviewers: the data is anonymously accessible for review purposes via the URL: https://data.donders.ru.nl/login/reviewer-246998468/fb3458de-637d-402e-a27a-9ae386c4decd. The permanent link to the data will become openly accessible after acceptance of the manuscript. The underlying code for this study is available to the reviewers and editor upon request and will be made openly accessible after acceptance of the manuscript.]

For Review Only

# Results

## Participants

Table 1 displays the characteristics of the included participants. On average, the PD group (Hoehn & Yahr stage 2-3) had a disease duration of 8.0 ± 3.9 years and an MDS-UPDRS III score of 43.7 ± 10.5 (max. score: 132). They all reported to have moderate to severe freezing in daily life with a NFOGQ score of 19.4 ± 3.5 (max. score: 28). The two groups did not differ significantly in age, gender, or cognition, but the PD group scored significantly higher for questions inquiring about levels of anxiety or depression.

Table 1: Participant characteristics

| | PD (n = 23) | HC (n = 22) | *p*-value* |
|---|---|---|---|
| Age (years) | 66.6 ± 8.9 | 65.9 ± 10.1 | 0.82 |
| Sex (% man) | 87% | 82% | / |
| Hand dominance (% right-handed) | 91% | 86% | / |
| NFOGQ | 19.4 ± 3.5 | / | / |
| % time frozen | 13.0 ± 17.4 | / | / |
| MDS-UPDRS part III | 43.7 ± 10.5 | / | / |
| Disease duration (years) | 8.0 ± 3.9 | / | / |
| Levodopa Equivalent Daily Dose (mg) | 1080.3 ± 345.2 | / | / |
| Years of education | 17.4 ± 5.8 | 15.1 ± 4.5 | 0.15 |
| MoCA | 25.9 ± 3.0 | 26.3 ± 3.2 | 0.67 |
| TMT part B – A (s) | 71.4 ± 103.6 | 43.9 ± 46.4 | 0.27 |
| HADS | 8.5 ± 5.2 | 4.2 ± 4.7 | 0.007 |
| Anxiety levels | 2.7 ± 12.0 | 0.2 ± 0.5 | < 0.001 |

Values indicate mean ± standard deviation. **p*-values of two-sample t-test. (MoCA = Montreal Cognitive Assessment (range: 0 – 30), TMT = Trail Making Test, HADS = Hospital Anxiety and Depression Scale (range 0 – 42), anxiety levels (range 0 – 21), MDS-UPDRS = Movement Disorders Society's Unified Parkinson's Disease Rating Scale (range: 0 – 132), NFOGQ = New Freezing of Gait Questionnaire (range 0 – 28))

The median number of freezing episodes per PD participant that were observed during the gait task was 15 (IQR: [3.25 – 32.75], range: 0 – 82) including the three participants that did not experience any freezing during the protocol. 50% of the episodes were triggered by turning (in 19 of 23 participants), 24% by doorway passing (12 participants), 16% by destination freezing (9 participants), 6% by starting (5 participants), and 5% during straight walking. Regarding the phenotypical presentation, 92% of the episodes were of the trembling-shuffling type and 8% of the akinetic type (14 participants trembling-shuffling only, 1 participant akinesia only, and 5

For Review Only

participants mixed). The median duration of a freezing episode was 3.8 s (IQR: [1.9 – 7.2 s], range: 0.4 – 263.8 s).

## PD vs HC groups

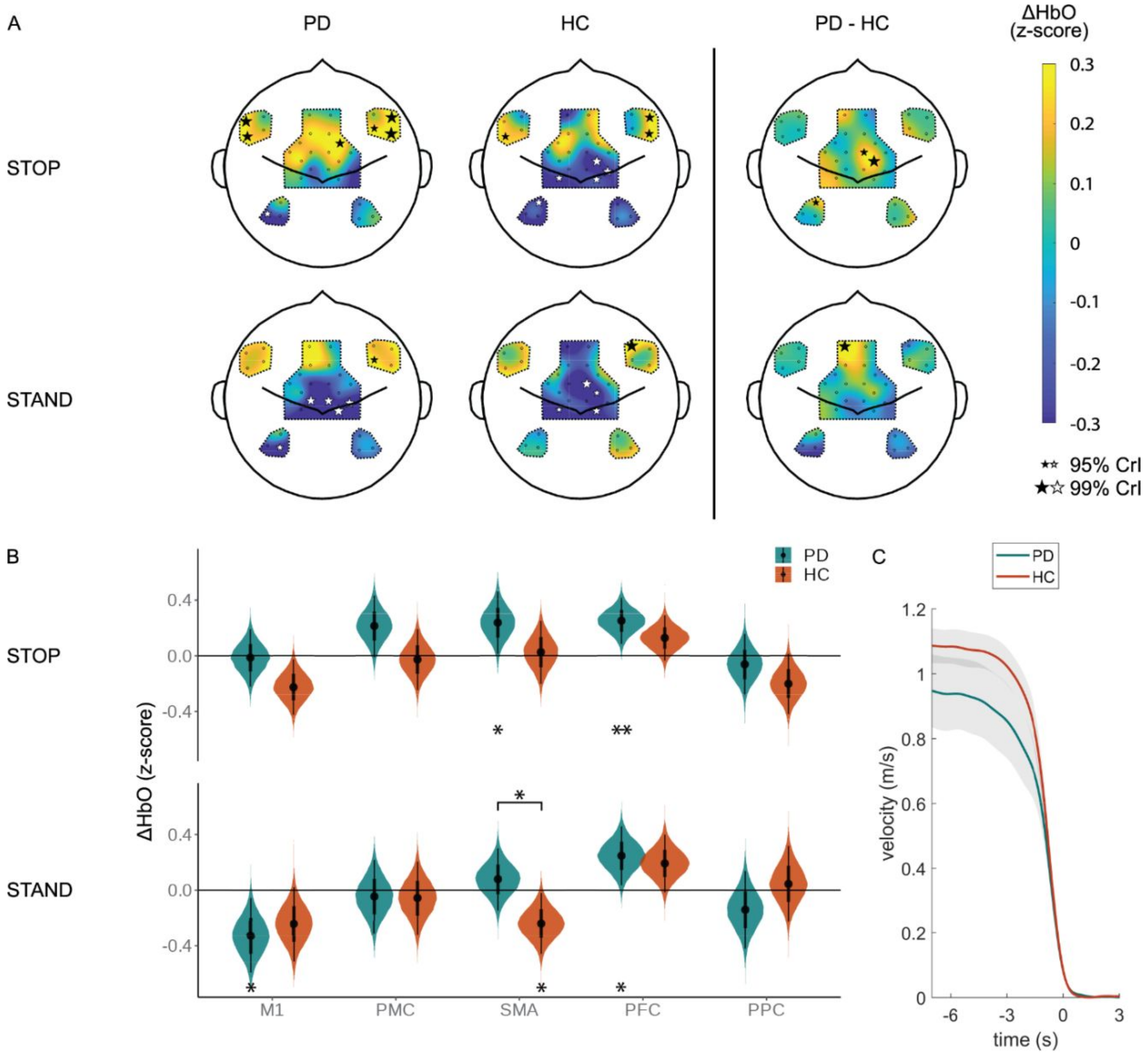


Fig. 3: Stopping and standing. (A) Cortical activity maps of estimated ΔHbO responses 0 to 3 s after stopping (stop) and 7 to 10 s after stopping (stand) compared to baseline (-10 -5 s). The black and white stars indicate channels with 95% credibility intervals (CrI) of the posterior probabilities excluding zero (small star) or 99% of the CrI excluding zero (large star). The size of the star scales with the probability that the estimated ΔHbO excludes zero. (B) Posterior probabilities of the estimated average ΔHbO responses for each ROI. The stars underneath the violin plots indicate if the posterior probability of the estimated ΔHbO response is different from baseline; the stars above the violin plots indicate if the posterior probability of the estimated ΔHbO response differs between the groups (* = 95% CrI excluding zero; ** = 99% CrI excluding zero). (C) Average walking velocity for the two study groups when stopping (t = 0 s). The grey areas indicate the 95% confidence intervals. (PD = Parkinson's Disease group; HC = healthy control group; ΔHbO = change in oxygenated hemoglobin; CrI = credibility interval; ROI = region of interest; M1: primary motor cortex; PMC: premotor cortex; SMA: supplementary motor area; PFC: prefrontal cortex; PPC: posterior parietal cortex)

## Stopping and standing

Fig. 3A shows the cortical activity of the PD and HC group during stopping (0 to 3 s after stop event) and standing (7 to 10 s after stop event). During stops, the PD group displayed widespread activity in the premotor and prefrontal areas, while the HC group showed similar but more focused activity in these areas. Posterior probabilities of the estimated ΔHbO responses in the different ROIs (Fig. 3B), revealed that the PD group increased activity in the SMA (mean [95% credibility interval (CrI)]: 0.24 [0.01 0.46]) and PFC (0.25 [0.09 0.42]), while the HC group decreased activity in the M1 during stopping (-0.23 [-0.42 -0.03]). When standing still, M1 activity globally was decreased compared to baseline levels for both groups (global intercept: -0.29 [-0.48 -0.09]). The SMA showed substantive differences between the PD group and the HC group, with higher activity in the PD group than in the HC group (group: 0.16 [0.00 0.31]).

On average, the PD group walked slower than the HC group (PD: 0.95 m/s; HC: 1.09 m/s; two-sample t-test: $p = 0.04$), and decelerated slower than the HC group in the last 3 s before coming to a stop (PD: -0.24 m/s²; HC: -0.30 m/s²; two-sample t-test: $p = 0.008$) (Fig. 3C).

## Turning and doorways

The PD group showed more extensive and higher activity of the premotor areas during turning than the HC group (Fig. 4A), with Bayesian statistics providing evidence for higher activity in the PMC (group: 0.17 [0.07 0.26]) and the SMA (group: 0.12 [0.01 0.23]) (Fig.4B). Similarly, the PD group showed higher activity during doorway passage in the PMC, however, this effect was smaller, and the 95% CrI did not exclude zero (group: 0.04 [-0.08 0.15]) (Suppl. Fig. 5). Because not all participants experienced doorway freezing during the task, we performed a

For Review Only

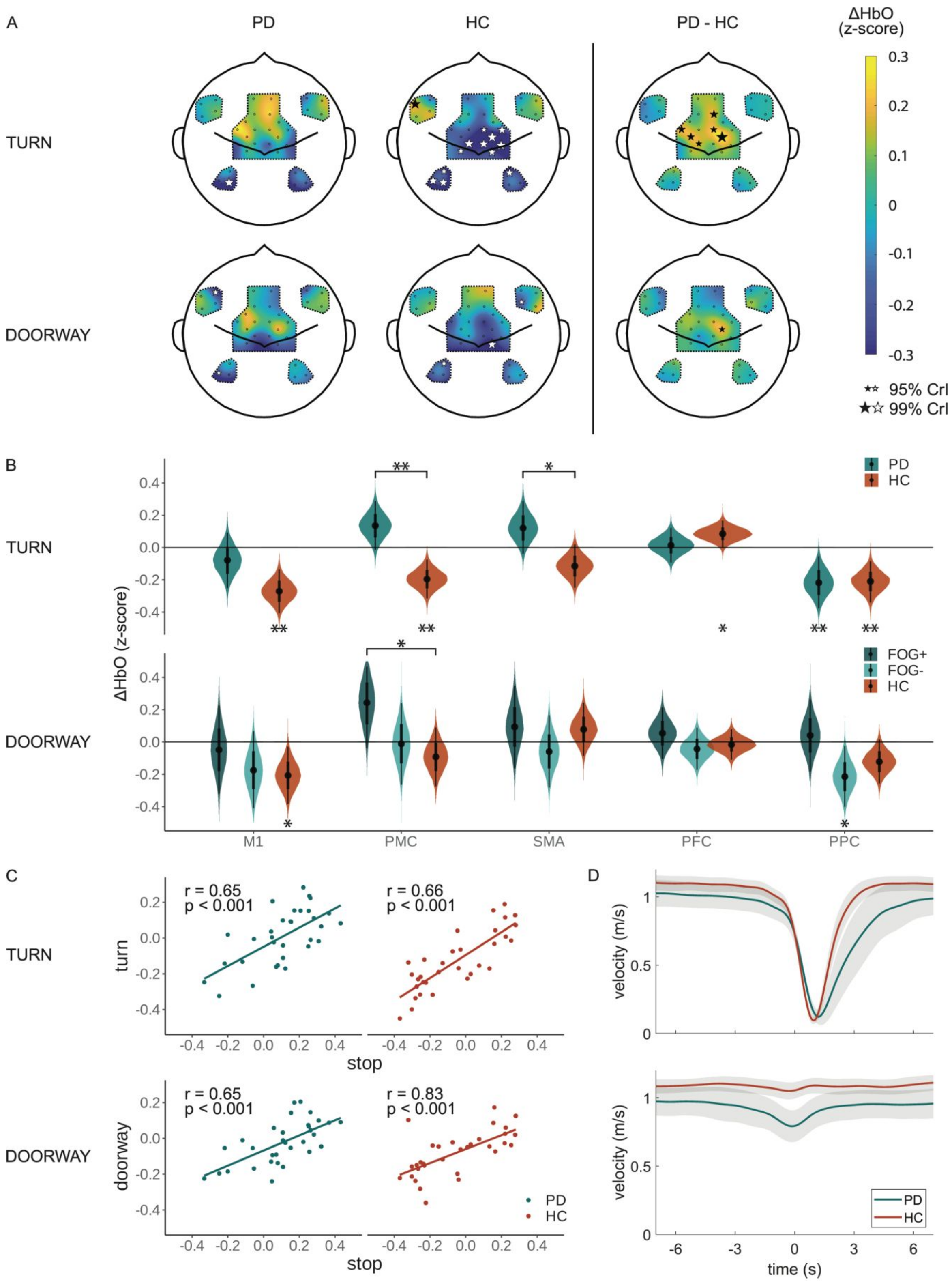


Fig. 4: Turns and doorways. (A) Cortical activity maps of estimated ΔHbO responses 0 to 3 s after turning and 0 to 3 s after doorway passing compared to baseline (-10 -5 s). The black and white stars indicate channels with 95% credibility interval (CrI) of the posterior probabilities excluding zero (small star) or 99% of the CrI excluding zero (large star). The size of the star scales with the probability that the estimated ΔHbO excludes zero. (B) Posterior probabilities of the estimated average ΔHbO responses for each ROI. The stars underneath the violin plots indicate if the posterior probability of the estimated ΔHbO response is different from baseline; the stars above the violin plots indicate if the posterior probability of the estimated ΔHbO response differs between the groups (* = 95% CrI excluding zero; ** = 99% CrI excluding zero). For the doorway condition we present the subgroup analysis which the PD group split into participants that experienced doorway freezing during the study (FOG+, $n$ = 10, darker green) and that did not experience doorway freezing (FOG-, $n$ = 13, lighter green). [illegible] Supplementary Fig. 5

https://mc.manuscriptcentral.com/braincom

(C) correlations between the estimated cortical activity of each channel during stopping (x-axis) and during turns/doorways (y-axis). Each dot represents a different channel. (D) Average walking velocity for the two groups when turning (upper plot) and when walking through the doorway (lower plot). The grey areas indicate the 95% confidence intervals. Timepoint zero represents the onset of the turn/doorway passage. For abbreviations: see Fig. 3.

follow-up analysis in which we split the PD group in a subgroup that experienced doorway freezing (FOG+, $n = 12$) and a subgroup that did not experience doorway freezing during the protocol (FOG-, $n = 11$). This follow-up analysis revealed a higher PMC activity in the FOG+ group than in the HC group during doorway passage (group: 0.34 [0.00 0.68]) (Fig. 4B).

Interestingly, we observed a remarkable resemblance between the cortical activity maps of turning and doorway passage on the one hand (Fig. 4A) and the cortical activity maps of stopping on the other hand (Fig. 3A). Therefore, we exploratively plotted the model estimates of each channel during stopping vs the model estimates of each channel during turning/doorway passage (Fig. 4C) and calculated the Pearson correlation coefficients between the two. This yielded significant correlations ($p < 0.001$) with correlation coefficients of > 0.65 in both study groups.

Analysis of the walking speed showed that the PD group decelerated slower than the HC group in the 2 s *before* turning (HC: -0.15 m/s$^2$; PD: -0.09 m/s$^2$; unpaired t-test: $p = 0.009$), and that the PD group turned slower than the HC group (PD: 3.07 s; HC: 1.89 s; unpaired t-test: $p < 0.001$) (Fig. 4D). During doorway passage, the PD group significantly reduced their walking speed 2 s before encountering the doorway (-0.04 m/s$^2$; one-sample t-test: $p < 0.001$) while the HC group maintained the same speed (-0.01 m/s$^2$; one-sample t-test: $p = 0.11$) resulting in a significant difference between the groups (two-sample t-test: $p = 0.05$).

## Starting and walking

The cortical activity during starting and walking are presented in Supplementary Fig. 4. Overall, we observed first a decrease in activity in M1 and PMC when starting to walk (M1: -0.15 [-

For Review Only

422 0.28 -0.03], PMC: -0.17 [-0.29 -0.05]) which was followed by an increase in activity in M1 423 during walking (M1: 0.22 [0.08 0.36]). None of the 95% CrI of the group effects excluded zero. 424 The PD group accelerated slower than the HC group in the first 3 s after the start signal (PD: 425 0.16 m/s²; HC: 0.26 m/s²; two-sample t-test: $p = 0.003$) (Supplementary Fig. 4C).

### 426 Correlations with freezing severity

427 We found a positive correlation between PFC activity during a normal doorway passage and 428 the % time frozen due to doorway freezing in the PD group, also after correcting for MDS-429 UPDRS III scores (mean [95% CrI]: 0.28 [0.08 0.49]). PFC activity during stopping was 430 correlated with the MDS-UPDRS III score (0.17 [0.00 0.33]). No other correlations were found 431 in the other ROIs, nor for the other normal gait events (turning, standing, starting, walking).

## 432 Freezing vs stopping vs successful gait events

433 When comparing cortical activity during freezing with voluntary stops and successful gait 434 events, we observed substantial differences in the PFC between freezing and stopping: the PFC 435 activity was lower during freezing than during stopping, both for freezing elicited by turning 436 and for freezing elicited by doorway passage (turn freezing: -0.26 [-0.51 0.00]; doorway 437 freezing: -0.50 [-0.82 -0.22]) (Fig. 5). Additionally, PFC activity during doorway freezing was 438 decreased compared to baseline (-0.25 [-0.51 0.00]). The other ROIs did not show differences 439 compared to the freezing condition with 95% CrI excluding zero.

For Review Only

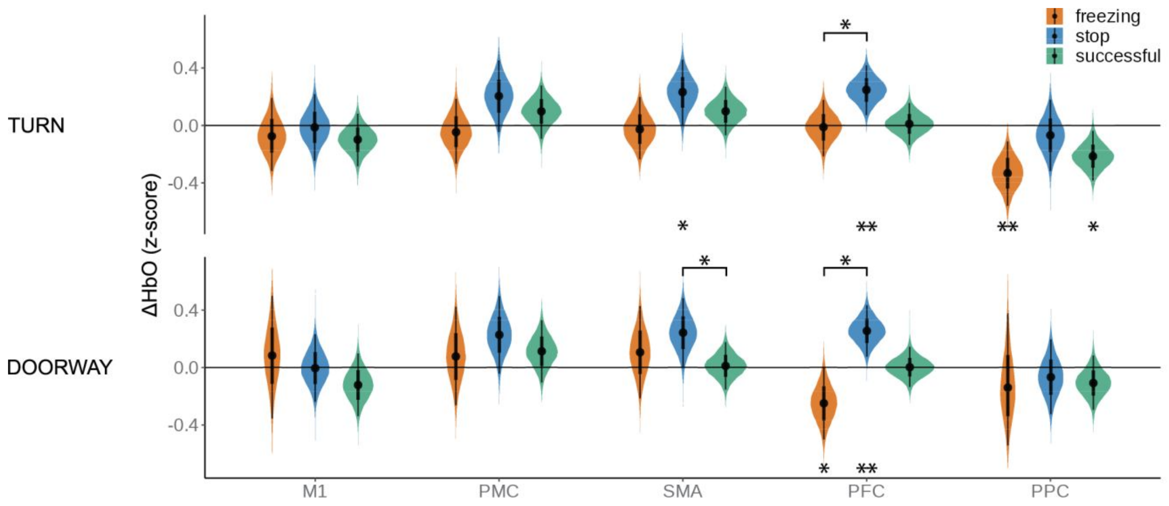


Fig. 5: Freezing of gait vs stopping vs successful gait events. Posterior probabilities of the estimated average ΔHbO responses for each ROI during freezing of gait (*freezing*, orange), stopping (*stop*, blue), and successful gait events (*successful*, green). The stars underneath the plots indicate if the posterior probability of the estimated ΔHbO response is different from ; the stars above the violin plots indicate if the posterior probability of the estimated ΔHbO response differs between the groups (* = 95% CrI excluding zero; ** = 99% CrI excluding zero). The cortical activity map during freezing is displayed in Box 1. For abbreviations: see Fig. 3.

# Discussion

We used fNIRS to investigate cortical activity related to freezing of gait during free ambulation. We compared cortical activity of people with freezing to age-matched healthy controls and observed higher and more widespread activity in the premotor areas during turning and gait termination. Similar effects were observed during doorway passing, but only when considering a subgroup that effectively experienced doorway freezing in the experiment. Interestingly, the cortical activity during turning and doorway passing was correlated with the cortical activity during stopping. Although this suggests that freezing is related to stopping, freezing was different from stopping by having lower PFC activity. Taken together, we postulate that freezing might result from an imbalance between the SMA and the PFC within the stopping network. We furthermore highlight the advantage of fNIRS to investigate gait during free ambulation, but note the need for careful correction of confounds occurring during walking (Box 1).

## The premotor areas are consistently related to freezing pathophysiology

The increased premotor area activity in the PD group during turning and doorway passage is in line with previous fMRI studies[44,45,78] and one previous fNIRS study.[79] Even while there were differences between the fMRI studies' findings of increased or decreased activity, they all identified the SMA as a significant locus of action. The fNIRS study reported increased SMA and PMC activity during turning in people with PD compared to controls, but not in freezers.[79] However, this study did not specify whether freezing episodes were excluded from analysis. Additionally, we observed widespread SMA activity in the PD group during gait termination. This SMA activity was still present 7 to 10 s after stopping, suggesting prolonged activation of this area after having come to a standstill.

Taken together, these results provide support for the role of the SMA in the pathophysiology of freezing. It has been hypothesized that excessive SMA recruitment leads to disrupted communication with the subthalamic nucleus (STN), leading to involuntary activation of the hyperdirect pathway, hence putting a brake on ongoing or initiating movements.[18,44,45] We speculate that the prolonged SMA activity after coming to a stop might explain why people with Parkinson’s disease have greater difficulty initiating gait, as they would require to first deactivate the hyperdirect pathway before taking the first step.[80] Alternatively, the increased SMA activity during standing relative to the walking baseline might also represent a decreased SMA activity during walking instead.[24,81-83]

Although we did find correlations of PFC activity with freezing severity during doorway passing and with the MDS-UPDRS motor scores for successful stopping, we did not find correlations of the SMA/PMC activity with freezing severity or MDS-UPDRS. It is therefore not entirely clear whether the observed premotor overactivity relates to freezing of gait



https://mc.manuscriptcentral.com/braincom

specifically, or to Parkinson's disease in general, although the subgroup analysis of the doorway freezers suggests a more freezing-specific relationship. This study was specifically designed to include persons that reported to have freezing at least daily to increase chances to capture cortical activity during actual freezing episodes. Nevertheless, future studies could also include participants without freezing, so better conclusions can be made about the nature of the premotor area overactivity.

## Freezing might be related to stopping

Stopping induced activation of the SMA and PFC in the PD group, and post hoc analysis revealed correlations between the cortical activation patterns during stopping and turning, and stopping and doorway passage, for both study groups. The SMA and PFC, or more specifically, the SMA and the Inferior Frontal Gyrus (IFG), are well-known for their role in inhibiting actions.[84] They are considered to be part of the stopping network,[85-87] and have previously been associated with imagined termination of gait.[88] From the estimated channel positions (Supplementary Table 3), we can infer that our channels mainly covered this gyrus of the PFC. The correlations suggest that turning and doorway passage activate a *preparatory* stopping network, hence might explain why these actions are prone to elicit freezing (note that hemodynamic responses are delayed by approximately 6 s). Such a preparatory stopping network, often referred to as the *proactive inhibitory control network* and also including the SMA and IFG, has been reported to facilitate stopping behavior in case a quick brake is required.[85,86,89] For example, when people walk towards a door, they may not be certain about what they will encounter behind the door, or they might be unsure whether they would be able to pass through without any collisions. In this study specifically, the alternation between stopping in front of the door/in the square and continued walking might have played an additional factor.

For Review Only

Similar to the proactive stopping network, the "hold-your-horses" principle postulates that a stopping network is activated in case multiple motor programs are competing with each other to withhold the motor response until a final decision is made.[90] This principle has previously been proposed as a possible explanation for freezing.[43] However, following this principle, we would not expect the HC group to show similar correlations between doorway passing and stopping, as they have no reason to exhibit conflicting motor programs. Another explanation for the similarity in cortical activity between turning/doorways and stopping is that both patterns represent a switch in motor programming, rather than the activation of a stopping network.

Although both groups showed correlations with their respective stopping activation patterns during doorway passage, only the PD group slowed down at the door, while the HC group maintained a constant speed. This slowing of PD patients is in line with previous behavioral studies showing that freezers decrease their walking speed at narrow doorways.[91-93] Consequently, the HC group seems to be more successful in suppressing the stopping program, or alternatively, the PD group is more sensitive to the stopping signals.

For Review Only

## Freezing is not the same as stopping

The observation that freezing might be related to stopping, prompts the question whether freezing and stopping share a similar mechanism. When directly comparing freezing to stopping, the PFC (IFG) showed distinct activity, with an increase in activity during stopping, but a decrease (doorway freezing) or no change (turning freezing) in activity during freezing. Moreover, the IFG activity during stopping correlated with the MDS-UPDRS scores (higher activity for worse Parkinson's disease symptoms). Based on this observation, we suggest that IFG activation is crucial for voluntary stopping strategies. The precise role of the IFG and SMA within the stopping network is still under debate, but IFG is thought to be critical for attentional

monitoring and stop-signal detection, while SMA is thought to be a direct communicator with the STN establishing the hyperdirect pathway.[85,94]

An important note is that the relatively low PFC (IFG) activity during freezing is opposite of what has been reported previously. A previous fNIRS study measuring PFC activity during turn freezing reported increased PFC activity during freezing episodes.[27] This study, however, did not correct for potential systemic confounds like changes in heart rate, blood pressure, etc. Indeed, when we did not apply a short channel correction to our data, we observed similar increases of fNIRS signals globally over the whole scalp during freezing (Box 1) and in all the short channels, measuring only superficial scalp hemodynamics. Moreover, the fNIRS signals during freezing and stopping resembled the time courses of heart rate data that have been reported previously[95,96], suggesting a systemic origin of the previously observed increased fNIRS activity. Another fMRI study using virtual reality and foot pedaling reported increased BOLD responses in the IFG during abnormally long foot pedaling latencies – which were considered the clinical correlate for a freezing episode.[43] However, the question remains how well these motor arrests correspond to freezing during free ambulation. Another possible explanation for the discrepancy between our study and the study from Shine and colleagues is that the respective region of interest did not correspond. Nevertheless, when comparing the MNI coordinates of both studies, the highlighted area from that study was close to our optode array (Supplementary Fig. 7).

For Review Only

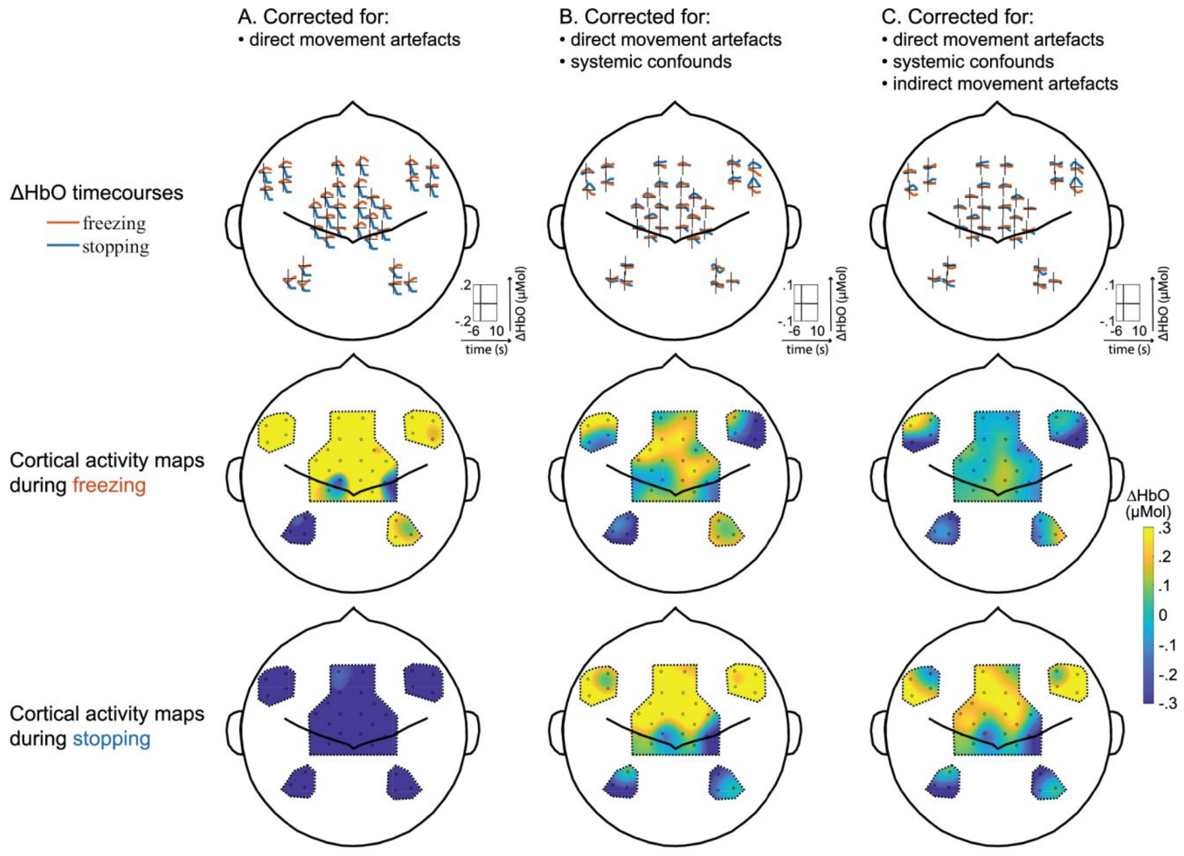


**The necessity of correction for confounds. (A)** ΔHbO time courses and cortical activity maps during freezing (red line) and stopping (blue line) when only corrected for direct movement artefacts with spline interpolation and wavelet filtering. The cortical activity maps represent the average ΔHbO response 0 to 3 s after freezing and stopping onset. **(B)** ΔHbO time courses and cortical activity maps when additionally corrected for systemic confounds with short channels. **(C)** ΔHbO time courses and cortical activity maps when additionally corrected for indirect movement artefacts with motion data of the head.

The measurement of cortical signals during gait with fNIRS is now possible thanks to modern equipment, but still not trivial.[40] First, walking induces large ***systemic physiological confounds*** such as changes in heart rate, blood pressure, and breathing patterns.[60,61] Systemic physiological changes confound the fNIRS signals as they induce direct changes in the hemoglobin concentrations intra- and extracerebrally.[98,99] Second, walking introduces movement artifacts due to head movements, which can be separated into direct and indirect movement artifacts.[62] ***Direct movement artifacts*** are caused by direct mechanical decoupling between the optodes and the scalp, allowing environmental light to enter the optical path, and induce spikes and baseline shifts in the signal.[100] ***Indirect movement artifacts*** are caused by local blood flow shifts due to head changes in head position and induce slower confounding signals.[62]

We corrected for the potential confounds of walking by performing a regression analysis with 16 short channels intended to only measure superficial scalp hemodynamics (*systemic confounds*), and with movement data of the head including acceleration, angular acceleration, and orientation (*indirect movement artifacts*). *Direct movement artifacts* were corrected with a combination of spline interpolation[54] and wavelet correction,[55] which is currently considered the best method for direct movement artifact correction.[56-58] Of note, we observed that participants looked down when stopping, turning, freezing, and even when passing a doorway (Suppl. Fig. 1). We accounted for this by including head orientation in the regression analysis, though there is currently no standard practice for this in fNIRS research. The effects of the systemic artifact correction and indirect movement artefact correction are displayed here above and are not to be underestimated: the conclusions of this paper could have been considerably different, had the confounds not been considered.

The increase in M1 activity during walking and decrease in activity during stopping is in line with our expectations and consistent with previous gait studies using SPECT, fMRI, or fNIRS.[24,101-104] The involvement of the SMA and PFC (IFG) during stopping is easy to link to the general stopping network,[86] and has also previously been associated with imagined termination of gait.[89] Nevertheless, we cannot rule out that the current results still hold some bias. For example, we did not account for the non-instantaneousness of indirect movement artifacts,[62,64] or did not directly account for cardiorespiratory changes by simultaneously measuring heart rate, blood pressure, or breathing behavior during the protocol.[105] Future studies should take into account these potential sources of bias.

Box 1: fNIRS requires careful correction for confounds of walking.

## Freezing: a paroxysmal imbalance between the SMA and IFG within the stopping network?

Taken together, we propose to extend the hypothesis of excessive SMA activity leading to freezing, by introducing an extra role of the IFG within the stopping network. Based on our results – and supported by other studies – we hypothesize that perfect coordination between the SMA and the IFG is essential to effectively transmit braking signals to the STN.[94] We compare this to the required cooperation between the switch and clutch of a (nonautomatic, stick shift) car. When switching gears, this needs to be accompanied by careful control of the clutch. If not, the car stalls. Hence, we could consider freezing as a failure of compensation by the IFG. The proposed mechanism might also explain the negative influence of dual-tasking on the occurrence of freezing, as a concomitant task might interfere with the attentional capacity of the IFG.[28,29,97,98]

For Review Only

Evidently, the proposed model is simplistic and should be challenged by future research. For example, perturbing the SMA and/or IFG with non-invasive transcranial stimulation could shed light on the differential roles of both areas in freezing pathophysiology. Moreover, modern deep brain stimulation devices, which are now able to record STN activity, could help to elucidate the role of the STN within the stopping network, especially when combined with cortical measurements of the SMA/IFG.[99,100] As a future application, a closed-loop deep brain stimulation system including SMA/IFG measurement could intervene during an upcoming or ongoing freezing episode by briefly suppressing the STN.[101]

## Conclusion

This study provides evidence for the role of the SMA in freezing of gait pathophysiology by investigating cortical activity during free ambulation. We extend upon this theory by suggesting

that freezing arises from a paroxysmal imbalance between the SMA and the IFG. Furthermore, we showed that IFG activity is lower during freezing than during stopping, which is opposite to previous findings,[27] but attributable to an improved correction for movement confounds in our study. Lastly, we provide recommendations for future fNIRS gait studies on how to correct for walking-induced artifacts (Box 1).

For Review Only

# Acknowledgements

First and foremost, we are grateful to all research participants who dedicated time and energy to make this study possible. We also thank patient-researchers Jan Gouman and Marc Bontjer who critically appraised the study design and results. We extend a thank you to Freek Nieuwhof for his valuable assistance during the initiation of the study, to David Mehler for his precious feedback on the performed statistics, and to Liucija Svinkunaite from Artinis Medical Systems and Günther Windau for their technical assistance with the experimental set-up. Finally, we are grateful to Ecaterina Savenco, Merel Tabor, Arne van Setten, Renée Jobse, Paula Koenders, Lennard van den Berg, and all other students for their assistance with the measurements.

# Funding

This work was supported by the Operational Program European Regional Development Fund (OP ERDF) of the European Union under the "PROMPT" project (PROJ-00872). The PROMPT project is a collaborative grant between two universities (Radboud University and the University of Twente), and three companies (Artinis Medical Systems, ANT Neuro, and Orikami). The Radboudumc Center of Expertise for Parkinson & Movement Disorders was supported by a center of excellence grant of the Parkinson's Foundation. The funders or companies played no role in study design, data collection, analysis and interpretation of data, or the writing of this manuscript.


For Review Only

# Competing interests

All authors declare no financial or non-financial competing interests.

# Supplementary material

Supplementary material is available at *Brain* online.

# Author Contribution Statement

HMC was responsible for the conceptualization and design of the study. She collected the research data, annotated the freezing of gait episodes on video, performed the analyses, created the figures, and was the major writer of the manuscript. RO contributed to the concept and design of the study, supervised the data analysis, and reviewed and edited the manuscript. YAFR annotated freezing of gait episodes on video and reviewed and edited the manuscript. BRB contributed to the funding acquisition, and the concept and design of the study. He reviewed and edited the manuscript. IGMC contributed to the funding acquisition, and the concept and design of the study. He supervised the data analysis, and reviewed and edited the manuscript. RJAW was responsible for the funding acquisition, he contributed to the concept and design of the study, supervised the data analysis, and reviewed and edited the manuscript. All authors read and approved the final manuscript.

For Review Only

# References


1 Ge, H. L. *et al.* The prevalence of freezing of gait in Parkinson's disease and in patients with different disease durations and severities. *Chin Neurosurg J* **6**, 17, doi:10.1186/s41016-020-00197-y (2020).

2 Forsaa, E. B., Larsen, J. P., Wentzel-Larsen, T. & Alves, G. A 12-year population-based study of freezing of gait in Parkinson's disease. *Parkinsonism Relat Disord* **21**, 254-258, doi:10.1016/j.parkreldis.2014.12.020 (2015).

3 Tosserams, A., Mazaheri, M., Vart, P., Bloem, B. R. & Nonnekes, J. Sex and freezing of gait in Parkinson's disease: a systematic review and meta-analysis. *J. Neurol.* **268**, 125-132, doi:10.1007/s00415-020-10117-w (2021).

4 Nutt, J. G. *et al.* Freezing of gait: moving forward on a mysterious clinical phenomenon. *Lancet Neurol* **10**, 734-744, doi:10.1016/S1474-4422(11)70143-0 (2011).

5 Moore, O., Peretz, C. & Giladi, N. Freezing of gait affects quality of life of peoples with Parkinson's disease beyond its relationships with mobility and gait. *Mov Disord* **22**, 2192-2195, doi:10.1002/mds.21659 (2007).

6 Bloem, B. R., Hausdorff, J. M., Visser, J. E. & Giladi, N. Falls and freezing of gait in Parkinson's disease: a review of two interconnected, episodic phenomena. *Mov Disord* **19**, 871-884, doi:10.1002/mds.20115 (2004).

7 Kerr, G. K. *et al.* Predictors of future falls in Parkinson disease. *Neurology* **75**, 116-124, doi:10.1212/WNL.0b013e3181e7b688 (2010).

8 Rahman, S., Griffin, H. J., Quinn, N. P. & Jahanshahi, M. Quality of life in Parkinson's disease: the relative importance of the symptoms. *Mov Disord* **23**, 1428-1434, doi:10.1002/mds.21667 (2008).

9 Perez-Lloret, S. *et al.* Prevalence, determinants, and effect on quality of life of freezing of gait in Parkinson disease. *JAMA Neurol* **71**, 884-890, doi:10.1001/jamaneurol.2014.753 (2014).

10 Weiss, D. *et al.* Freezing of gait: understanding the complexity of an enigmatic phenomenon. *Brain* **143**, 14-30, doi:10.1093/brain/awz314 (2020).

11 Gilat, M. *et al.* Freezing of gait: Promising avenues for future treatment. *Parkinsonism Relat Disord* **52**, 7-16, doi:10.1016/j.parkreldis.2018.03.009 (2018).

12 Lewis, S. *et al.* Stepping up to meet the challenge of freezing of gait in Parkinson's disease. *Transl Neurodegener* **11**, 23, doi:10.1186/s40035-022-00298-x (2022).

13 Lewis, S. J. G. *et al.* Addressing the Challenges of Clinical Research for Freezing of Gait in Parkinson's Disease. *Mov. Disord.*, doi:10.1002/mds.28837 (2021).

14 Nonnekes, J. *et al.* Freezing of gait: a practical approach to management. *Lancet Neurol* **14**, 768-778, doi:10.1016/S1474-4422(15)00041-1 (2015).

15 Lewis, S. J. & Shine, J. M. The Next Step: A Common Neural Mechanism for Freezing of Gait. *Neuroscientist* **22**, 72-82, doi:10.1177/1073858414559101 (2016).

16 Bardakan, M. M. *et al.* Imaging the neural underpinnings of freezing of gait in Parkinson's disease. *NeuroImage: Clinical* **35**, doi:10.1016/j.nicl.2022.103123 (2022).

17 Jacobs, J. V., Nutt, J. G., Carlson-Kuhta, P., Stephens, M. & Horak, F. B. Knee trembling during freezing of gait represents multiple anticipatory postural adjustments. *Exp. Neurol.* **215**, 334-341, doi:10.1016/j.expneurol.2008.10.019 (2009).

18 Jacobs, J. V., Lou, J. S., Kraakevik, J. A. & Horak, F. B. The supplementary motor area contributes to the timing of the anticipatory postural adjustment during step initiation in participants with and without Parkinson's disease. *Neuroscience* **164**, 877-885, doi:10.1016/j.neuroscience.2009.08.002 (2009).


For Review Only

19 Lewis, S. J. & Barker, R. A. A pathophysiological model of freezing of gait in Parkinson's disease. *Parkinsonism Relat Disord* **15**, 333-338, doi:10.1016/j.parkreldis.2008.08.006 (2009).

20 Snijders, A. H. *et al.* Gait-related cerebral alterations in patients with Parkinson's disease with freezing of gait. *Brain* **134**, 59-72, doi:10.1093/brain/awq324 (2011).

21 Shine, J. M., Ward, P. B., Naismith, S. L., Pearson, M. & Lewis, S. J. Utilising functional MRI (fMRI) to explore the freezing phenomenon in Parkinson's disease. *J Clin Neurosci* **18**, 807-810, doi:10.1016/j.jocn.2011.02.003 (2011).

22 Peterson, D. S., Pickett, K. A., Duncan, R. P., Perlmutter, J. S. & Earhart, G. M. Brain activity during complex imagined gait tasks in Parkinson disease. *Clin Neurophysiol* **125**, 995-1005, doi:10.1016/j.clinph.2013.10.008 (2014).

23 Matsui, H. *et al.* Three-dimensional stereotactic surface projection study of freezing of gait and brain perfusion image in Parkinson's disease. *Mov Disord* **20**, 1272-1277, doi:10.1002/mds.20520 (2005).

24 Hanakawa, T. *et al.* Mechanisms underlying gait disturbance in Parkinson's disease: a single photon emission computed tomography study. *Brain* **122 ( Pt 7)**, 1271-1282, doi:10.1093/brain/122.7.1271 (1999).

25 Herold, F. *et al.* Functional near-infrared spectroscopy in movement science: a systematic review on cortical activity in postural and walking tasks. *Neurophotonics* **4**, 041403, doi:10.1117/1.NPh.4.4.041403 (2017).

26 Menant, J. C. *et al.* A consensus guide to using functional near-infrared spectroscopy in posture and gait research. *Gait Posture* **82**, 254-265, doi:10.1016/j.gaitpost.2020.09.012 (2020).

27 Maidan, I. *et al.* Changes in oxygenated hemoglobin link freezing of gait to frontal activation in patients with Parkinson disease: an fNIRS study of transient motor-cognitive failures. *J Neurol* **262**, 899-908, doi:10.1007/s00415-015-7650-6 (2015).

28 Vitorio, R., Stuart, S. & Mancini, M. Executive Control of Walking in People With Parkinson's Disease With Freezing of Gait. *Neurorehabil Neural Repair* **34**, 1138-1149, doi:10.1177/1545968320969940 (2020).

29 Belluscio, V., Stuart, S., Bergamini, E., Vannozzi, G. & Mancini, M. The Association between Prefrontal Cortex Activity and Turning Behavior in People with and without Freezing of Gait. *Neuroscience* **416**, 168-176, doi:10.1016/j.neuroscience.2019.07.024 (2019).

30 Dagan, M. *et al.* Dopaminergic therapy and prefrontal activation during walking in individuals with Parkinson's disease: does the levodopa overdose hypothesis extend to gait? *J. Neurol.* **268**, 658-668, doi:10.1007/s00415-020-10089-x (2021).

31 Postuma, R. B. *et al.* MDS clinical diagnostic criteria for Parkinson's disease. *Mov Disord* **30**, 1591-1601, doi:10.1002/mds.26424 (2015).

32 Postuma, R. B. *et al.* Validation of the MDS clinical diagnostic criteria for Parkinson's disease. *Mov Disord* **33**, 1601-1608, doi:10.1002/mds.27362 (2018).

33 Goetz, C. G. *et al.* Movement Disorder Society-sponsored revision of the Unified Parkinson's Disease Rating Scale (MDS-UPDRS): scale presentation and clinimetric testing results. *Mov Disord* **23**, 2129-2170, doi:10.1002/mds.22340 (2008).

34 Nieuwboer, A. *et al.* Reliability of the new freezing of gait questionnaire: agreement between patients with Parkinson's disease and their carers. *Gait Posture* **30**, 459-463, doi:10.1016/j.gaitpost.2009.07.108 (2009).

35 Nasreddine, Z. S. *et al.* The Montreal Cognitive Assessment, MoCA: a brief screening tool for mild cognitive impairment. *J Am Geriatr Soc* **53**, 695-699, doi:10.1111/j.1532-5415.2005.53221.x (2005).


For Review Only

36 Gaudino, E. A., Geisler, M. W. & Squires, N. K. Construct validity in the Trail Making Test: what makes Part B harder? *J Clin Exp Neuropsychol* **17**, 529-535, doi:10.1080/01688639508405143 (1995).

37 Pimenta, M. *et al.* Anxiety Independently Contributes to Severity of Freezing of Gait in People With Parkinson's Disease. *J Neuropsychiatry Clin Neurosci* **31**, 80-85, doi:10.1176/appi.neuropsych.17090177 (2019).

38 Martens, K. A. E. *et al.* Anxiety is associated with freezing of gait and attentional set-shifting in Parkinson's disease: A new perspective for early intervention. *Gait Posture* **49**, 431-436, doi:10.1016/j.gaitpost.2016.07.182 (2016).

39 Vitorio, R., Stuart, S., Rochester, L., Alcock, L. & Pantall, A. fNIRS response during walking - Artefact or cortical activity? A systematic review. *Neurosci Biobehav Rev* **83**, 160-172, doi:10.1016/j.neubiorev.2017.10.002 (2017).

40 Stuart, S. *et al.* Cortical activity during walking and balance tasks in older adults and in people with Parkinson's disease: A structured review. *Maturitas* **113**, 53-72, doi:10.1016/j.maturitas.2018.04.011 (2018).

41 Sato, T. *et al.* Reduction of global interference of scalp-hemodynamics in functional near-infrared spectroscopy using short distance probes. *Neuroimage* **141**, 120-132, doi:10.1016/j.neuroimage.2016.06.054 (2016).

42 Yucel, M. A. *et al.* Short separation regression improves statistical significance and better localizes the hemodynamic response obtained by near-infrared spectroscopy for tasks with differing autonomic responses. *Neurophotonics* **2**, 035005, doi:10.1117/1.NPh.2.3.035005 (2015).

43 Shine, J. M. *et al.* Exploring the cortical and subcortical functional magnetic resonance imaging changes associated with freezing in Parkinson's disease. *Brain* **136**, 1204-1215, doi:10.1093/brain/awt049 (2013).

44 Matar, E. *et al.* Identifying the neural correlates of doorway freezing in Parkinson's disease. *Hum Brain Mapp* **40**, 2055-2064, doi:10.1002/hbm.24506 (2019).

45 Gilat, M. *et al.* Brain activation underlying turning in Parkinson's disease patients with and without freezing of gait: a virtual reality fMRI study. *NPJ Parkinsons Dis* **1**, 15020, doi:10.1038/npjparkd.2015.20 (2015).

46 Ehgoetz Martens, K. A. *et al.* The functional network signature of heterogeneity in freezing of gait. *Brain* **141**, 1145-1160, doi:10.1093/brain/awy019 (2018).

47 Vercruysse, S. *et al.* The neural correlates of upper limb motor blocks in Parkinson's disease and their relation to freezing of gait. *Cereb Cortex* **24**, 3154-3166, doi:10.1093/cercor/bht170 (2014).

48 Homolle, S. & Oostenveld, R. Using a structured-light 3D scanner to improve EEG source modeling with more accurate electrode positions. *J Neurosci Methods* **326**, 108378, doi:10.1016/j.jneumeth.2019.108378 (2019).

49 Tzourio-Mazoyer, N. *et al.* Automated anatomical labeling of activations in SPM using a macroscopic anatomical parcellation of the MNI MRI single-subject brain. *Neuroimage* **15**, 273-289, doi:10.1006/nimg.2001.0978 (2002).

50 Oostenveld, R., Fries, P., Maris, E. & Schoffelen, J. M. FieldTrip: Open source software for advanced analysis of MEG, EEG, and invasive electrophysiological data. *Comput Intell Neurosci* **2011**, 156869, doi:10.1155/2011/156869 (2011).

51 Huppert, T. J., Diamond, S. G., Franceschini, M. A. & Boas, D. A. HomER: a review of time-series analysis methods for near-infrared spectroscopy of the brain. *Appl Opt* **48**, D280-298, doi:10.1364/ao.48.00d280 (2009).

52 Sappia, M. S., Hakimi, N., Colier, W. & Horschig, J. M. Signal quality index: an algorithm for quantitative assessment of functional near infrared spectroscopy signal quality. *Biomed Opt Express* **11**, 6732-6754, doi:10.1364/BOE.409317 (2020).

For Review Only

53 Scholkmann, F., Spichtig, S., Muehlemann, T. & Wolf, M. How to detect and reduce movement artifacts in near-infrared imaging using moving standard deviation and spline interpolation. *Physiol Meas* **31**, 649-662, doi:10.1088/0967-3334/31/5/004 (2010).

54 Molavi, B. & Dumont, G. A. Wavelet-based motion artifact removal for functional near-infrared spectroscopy. *Physiol Meas* **33**, 259-270, doi:10.1088/0967-3334/33/2/259 (2012).

55 Di Lorenzo, R. *et al.* Recommendations for motion correction of infant fNIRS data applicable to multiple data sets and acquisition systems. *Neuroimage* **200**, 511-527, doi:10.1016/j.neuroimage.2019.06.056 (2019).

56 Brigadoi, S. *et al.* Motion artifacts in functional near-infrared spectroscopy: a comparison of motion correction techniques applied to real cognitive data. *Neuroimage* **85 Pt 1**, 181-191, doi:10.1016/j.neuroimage.2013.04.082 (2014).

57 Cooper, R. J. *et al.* A systematic comparison of motion artifact correction techniques for functional near-infrared spectroscopy. *Front Neurosci* **6**, 147, doi:10.3389/fnins.2012.00147 (2012).

58 Scholkmann, F. & Wolf, M. General equation for the differential pathlength factor of the frontal human head depending on wavelength and age. *J Biomed Opt* **18**, 105004, doi:10.1117/1.JBO.18.10.105004 (2013).

59 Koenraadt, K. L., Roelofsen, E. G., Duysens, J. & Keijsers, N. L. Cortical control of normal gait and precision stepping: an fNIRS study. *Neuroimage* **85 Pt 1**, 415-422, doi:10.1016/j.neuroimage.2013.04.070 (2014).

60 Parvataneni, K., Ploeg, L., Olney, S. J. & Brouwer, B. Kinematic, kinetic and metabolic parameters of treadmill versus overground walking in healthy older adults. *Clin Biomech (Bristol, Avon)* **24**, 95-100, doi:10.1016/j.clinbiomech.2008.07.002 (2009).

61 Von Lühmann, A., Boukouvalas, Z., Müller, K.-R. & Adalı, T. A new blind source separation framework for signal analysis and artifact rejection in functional Near-Infrared Spectroscopy. *Neuroimage* **200**, 72-88, doi:10.1016/j.neuroimage.2019.06.021 (2019).

62 Santosa, H., Zhai, X., Fishburn, F., Sparto, P. J. & Huppert, T. J. Quantitative comparison of correction techniques for removing systemic physiological signal in functional near-infrared spectroscopy studies. *Neurophotonics* **7**, 035009, doi:10.1117/1.NPh.7.3.035009 (2020).

63 Von Lühmann, A., Li, X., Müller, K.-R., Boas, D. A. & Yücel, M. A. Improved physiological noise regression in fNIRS: A multimodal extension of the General Linear Model using temporally embedded Canonical Correlation Analysis. *Neuroimage* **208**, 116472, doi:10.1016/j.neuroimage.2019.116472 (2020).

64 Pinti, P., Scholkmann, F., Hamilton, A., Burgess, P. & Tachtsidis, I. Current Status and Issues Regarding Pre-processing of fNIRS Neuroimaging Data: An Investigation of Diverse Signal Filtering Methods Within a General Linear Model Framework. *Front Hum Neurosci* **12**, 505, doi:10.3389/fnhum.2018.00505 (2018).

65 Gilat, M. How to Annotate Freezing of Gait from Video: A Standardized Method Using Open-Source Software. *J Parkinsons Dis* **9**, 821-824, doi:10.3233/JPD-191700 (2019).

66 Cockx, H., Klaver, E., Tjepkema-Cloostermans, M., van Wezel, R. & Nonnekes, J. The Gray Area of Freezing of Gait Annotation: A Guideline and Open-Source Practical Tool. *Mov Disord Clin Pract* **9**, 1099-1104, doi:10.1002/mdc3.13556 (2022).

67 Burkner, P. C. brms: An R Package for Bayesian Multilevel Models Using Stan. *J Stat Softw* **80**, 1-28, doi:10.18637/jss.v080.i01 (2017).

68 Gelman, A., Hill, J. & Yajima, M. Why We (Usually) Don't Have to Worry About Multiple Comparisons. *J Res Educ Eff* **5**, 189-211, doi:10.1080/19345747.2011.618213 (2012).


For Review Only

69 Matzke, D., Boehm, U. & Vandekerckhove, J. Bayesian inference for psychology, part III: Parameter estimation in nonstandard models. *Psychon Bull Rev* **25**, 77-101, doi:10.3758/s13423-017-1394-5 (2018).

70 Algermissen, J. & Mehler, D. M. A. May the power be with you: are there highly powered studies in neuroscience, and how can we get more of them? *J Neurophysiol* **119**, 2114-2117, doi:10.1152/jn.00765.2017 (2018).

71 Gelman, A. & Tuerlinckx, F. A. Type S error rates for classical and Bayesian single and multiple comparison procedures. *Comput Stat* **15**, 373-390, doi:DOI 10.1007/s001800000040 (2000).

72 Taquet, M., Peters, J. M. & Warfield, S. K. 95-106 (Springer International Publishing).

73 Makowski, D., Ben-Shachar, M. S., Chen, S. H. A. & Ludecke, D. Indices of Effect Existence and Significance in the Bayesian Framework. *Front Psychol* **10**, 2767, doi:10.3389/fpsyg.2019.02767 (2019).

74 Wagenmakers, E. J. A practical solution to the pervasive problems of p values. *Psychon Bull Rev* **14**, 779-804, doi:Doi 10.3758/Bf03194105 (2007).

75 Wagenmakers, E.-J. *et al.* Bayesian inference for psychology. Part I: Theoretical advantages and practical ramifications. *Psychon Bull Rev* **25**, 35-57, doi:10.3758/s13423-017-1343-3 (2018).

76 Gorgolewski, K. J. *et al.* The brain imaging data structure, a format for organizing and describing outputs of neuroimaging experiments. *Sci Data* **3**, 160044, doi:10.1038/sdata.2016.44 (2016).

77 Luke, R. *et al. fNIRS-BIDS, the Brain Imaging Data Structure Extended to Functional Near-Infrared Spectroscopy* (OSFpreprints, 2023).

78 Peterson, D. S., Pickett, K. A., Duncan, R. P., Perlmutter, J. S. & Earhart, G. M. Brain activity during complex imagined gait tasks in Parkinson disease. *Clin. Neurophysiol.* **125**, 995-1005, doi:10.1016/j.clinph.2013.10.008 (2014).

79 Feng, H. *et al.* Cortical activation and functional connectivity during locomotion tasks in Parkinson's disease with freezing of gait. *Front Aging Neurosci* **15**, 1068943, doi:10.3389/fnagi.2023.1068943 (2023).

80 Brugger, F. *et al.* Altered activation and connectivity of the supplementary motor cortex at motor initiation in Parkinson's disease patients with freezing. *Clin Neurophysiol* **131**, 2171-2180, doi:10.1016/j.clinph.2020.05.023 (2020).

81 Sabatini, U. *et al.* Cortical motor reorganization in akinetic patients with Parkinson's disease: a functional MRI study. *Brain* **123 ( Pt 2)**, 394-403, doi:10.1093/brain/123.2.394 (2000).

82 Shine, J. M. *et al.* Differential neural activation patterns in patients with Parkinson's disease and freezing of gait in response to concurrent cognitive and motor load. *PLoS One* **8**, e52602, doi:10.1371/journal.pone.0052602 (2013).

83 Peterson, D. S., Pickett, K. A., Duncan, R., Perlmutter, J. & Earhart, G. M. Gait-related brain activity in people with Parkinson disease with freezing of gait. *PLoS One* **9**, e90634, doi:10.1371/journal.pone.0090634 (2014).

84 Simmonds, D. J., Pekar, J. J. & Mostofsky, S. H. Meta-analysis of Go/No-go tasks demonstrating that fMRI activation associated with response inhibition is task-dependent. *Neuropsychologia* **46**, 224-232, doi:10.1016/j.neuropsychologia.2007.07.015 (2008).

85 Aron, A. R. From reactive to proactive and selective control: developing a richer model for stopping inappropriate responses. *Biol Psychiatry* **69**, e55-68, doi:10.1016/j.biopsych.2010.07.024 (2011).


For Review Only

86 Zhang, F. & Iwaki, S. Common Neural Network for Different Functions: An Investigation of Proactive and Reactive Inhibition. *Front Behav Neurosci* **13**, 124, doi:10.3389/fnbeh.2019.00124 (2019).

87 Witt, K. The Subthalamic Nucleus in Impulsivity. *Decision Neuroscience: An Integrative Perspective*, 315-325, doi:10.1016/B978-0-12-805308-9.00025-7 (2017).

88 Wang, J. *et al.* Functional MRI in the assessment of cortical activation during gait-related imaginary tasks. *J Neural Transm (Vienna)* **116**, 1087-1092, doi:10.1007/s00702-009-0269-y (2009).

89 van den Wildenberg, W. P. M., Ridderinkhof, K. R. & Wylie, S. A. Towards Conceptual Clarification of Proactive Inhibitory Control: A Review. *Brain Sciences* **12**, doi:10.3390/brainsci12121638 (2022).

90 Frank, M. J. Hold your horses: a dynamic computational role for the subthalamic nucleus in decision making. *Neural Netw* **19**, 1120-1136, doi:10.1016/j.neunet.2006.03.006 (2006).

91 Almeida, Q. J. & Lebold, C. A. Freezing of gait in Parkinson's disease: a perceptual cause for a motor impairment? *J Neurol Neurosurg Psychiatry* **81**, 513-518, doi:10.1136/jnnp.2008.160580 (2010).

92 Cowie, D., Limousin, P., Peters, A., Hariz, M. & Day, B. L. Doorway-provoked freezing of gait in Parkinson's disease. *Mov Disord* **27**, 492-499, doi:10.1002/mds.23990 (2012).

93 Beck, E. N., Ehgoetz Martens, K. A. & Almeida, Q. J. Freezing of Gait in Parkinson's Disease: An Overload Problem? *PLoS One* **10**, e0144986, doi:10.1371/journal.pone.0144986 (2015).

94 Sebastian, A., Forstmann, B. U. & Matzke, D. Towards a model-based cognitive neuroscience of stopping - a neuroimaging perspective. *Neurosci Biobehav Rev* **90**, 130-136, doi:10.1016/j.neubiorev.2018.04.011 (2018).

95 Maidan, I. *et al.* Heart rate changes during freezing of gait in patients with Parkinson's disease. *Mov Disord* **25**, 2346-2354, doi:10.1002/mds.23280 (2010).

96 Cockx, H. *et al.* Dealing with the heterogeneous presentations of freezing of gait: how reliable are the freezing index and heart rate for freezing detection? *J. Neuroeng. Rehabil.* **20**, doi:10.1186/s12984-023-01175-y (2023).

97 Vandenbossche, J. *et al.* Freezing of gait in Parkinson's disease: disturbances in automaticity and control. *Front Hum Neurosci* **6**, 356, doi:10.3389/fnhum.2012.00356 (2012).

98 Wu, T., Hallett, M. & Chan, P. Motor automaticity in Parkinson's disease. *Neurobiol Dis* **82**, 226-234, doi:10.1016/j.nbd.2015.06.014 (2015).

99 Pozzi, N. G. *et al.* Freezing of gait in Parkinson's disease reflects a sudden derangement of locomotor network dynamics. *Brain* **142**, 2037-2050, doi:10.1093/brain/awz141 (2019).

100 Georgiades, M. J. *et al.* Hitting the brakes: pathological subthalamic nucleus activity in Parkinson's disease gait freezing. *Brain* **142**, 3906-3916, doi:10.1093/brain/awz325 (2019).

101 Petrucci, M. N. *et al.* Neural closed-loop deep brain stimulation for freezing of gait. *Brain Stimul* **13**, 1320-1322, doi:10.1016/j.brs.2020.06.018 (2020).

For Review Only

# Figure legends

**Fig. 1: Gait task.** Example of one run (+- 6.5 min) of the gait task. Participants walk up and down a corridor, halfway passing through a narrow doorway frame (60 cm wide), and at the ends making 180° turns in a square taped on the floor (50 cm wide). The distance between the doorway and the square was set at an individualized walking distance of approximately 20 s. Each 2.5 corridor length, participants were instructed to stop in front of the door or in the square, to make one step through the door or turn around after 15 s, and then to resume walking after another 15 s. The direction of the turns was alternated between each side of the corridor and this direction was switched after each instructed stop. Each participant completed approximately four runs in total.

For Review Only

**Fig. 2: fNIRS cap layout. (A)** Schematic representation of the cap layout indicating the positions of the long channels (30 mm interoptode distance) and short channels (10 mm interoptode distance) relative to the 10-20 EEG reference system and the main sulci. The recorded channel positions are presented in supplementary Fig. 7 and supplementary Table 3. **(B)** Images of the cap with the detectors in blue and the sources in yellow (dashed line for low output source). (M1 = primary motor cortex (yellow); PMC = premotor cortex (orange); SMA = supplementary motor area (purple); PFC = prefrontal cortex (red); PPC = posterior parietal cortex (green)).

**Fig. 3: Stopping and standing. (A)** Cortical activity maps of estimated ΔHbO responses 0 to 3 s after stopping (stop) and 7 to 10 s after stopping (stand) compared to baseline (-10 -5 s). The black and white stars indicate channels with 95% credibility intervals (CrI) of the posterior probabilities excluding zero (small star) or 99% of the CrI excluding zero (large star). The size of the star scales with the probability that the estimated ΔHbO excludes zero. **(B)** Posterior probabilities of the estimated average ΔHbO responses for each ROI. The stars underneath the

violin plots indicate if the posterior probability of the estimated ΔHbO response is different from baseline; the stars above the violin plots indicate if the posterior probability of the estimated ΔHbO response differs between the groups (* = 95% CrI excluding zero; ** = 99% CrI excluding zero). **(C)** Average walking velocity for the two study groups when stopping (t = 0 s). The grey areas indicate the 95% confidence intervals. (PD = Parkinson's Disease group; HC = healthy control group; ΔHbO = change in oxygenated hemoglobin; CrI = credibility interval; ROI = region of interest; M1: primary motor cortex; PMC: premotor cortex; SMA: supplementary motor area; PFC: prefrontal cortex; PPC: posterior parietal cortex)

**Fig. 4: Turns and doorways. (A)** Cortical activity maps of estimated ΔHbO responses 0 to 3 s after turning and 0 to 3 s after doorway passing compared to baseline (-10 -5 s). The black and white stars indicate channels with 95% credibility interval (CrI) of the posterior probabilities excluding zero (small star) or 99% of the CrI excluding zero (large star ). The size of the star scales with the probability that the estimated ΔHbO excludes zero. **(B)** Posterior probabilities of the estimated average ΔHbO responses for each ROI. The stars underneath the violin plots indicate if the posterior probability of the estimated ΔHbO response is different from baseline; the stars above the violin plots indicate if the posterior probability of the estimated ΔHbO response differs between the groups (* = 95% CrI excluding zero; ** = 99% CrI excluding zero). For the doorway condition we present the subgroup analysis which the PD group split into participants that experienced doorway freezing during the study (FOG+, $n$ = 10, darker green) and that did not experience doorway freezing (FOG-, $n$ = 13, lighter green). The original analysis is presented in Supplementary Fig. 5. **(C)** correlations between the estimated cortical activity of each channel during stopping (x-axis) and during turns/doorways (y-axis). Each dot represents a different channel. **(D)** Average walking velocity for the two groups when turning (upper plot) and when walking through the doorway (lower plot). The

For Review Only

grey areas indicate the 95% confidence intervals. Timepoint zero represents the onset of the turn/doorway passage. For abbreviations: see Fig. 3.

**Fig. 5: Freezing of gait vs stopping vs successful gait events.** Posterior probabilities of the estimated average ΔHbO responses for each ROI during freezing of gait (*freezing*, orange), stopping (*stop*, blue), and successful gait events (*successful*, green). The stars underneath the plots indicate if the posterior probability of the estimated ΔHbO response is different from ; the stars above the violin plots indicate if the posterior probability of the estimated ΔHbO response differs between the groups (* = 95% CrI excluding zero; ** = 99% CrI excluding zero). The cortical activity map during freezing is displayed in Box 1. For abbreviations: see Fig. 3.

**Box 1**: fNIRS requires careful correction for confounds of walking.

For Review Only

# Tables

Table 1: Participant characteristics

| | PD (n = 23) | HC (n = 22) | *p*-value* |
|---|---|---|---|
| Age (years) | 66.6 ± 8.9 | 65.9 ± 10.1 | 0.82 |
| Sex (% man) | 87% | 82% | / |
| Hand dominance (% right-handed) | 91% | 86% | / |
| NFOGQ | 19.4 ± 3.5 | / | / |
| % time frozen | 13.0 ± 17.4 | / | / |
| MDS-UPDRS part III | 43.7 ± 10.5 | / | / |
| Disease duration (years) | 8.0 ± 3.9 | / | / |
| Levodopa Equivalent Daily Dose (mg) | 1080.3 ± 345.2 | / | / |
| Years of education | 17.4 ± 5.8 | 15.1 ± 4.5 | 0.15 |
| MoCA | 25.9 ± 3.0 | 26.3 ± 3.2 | 0.67 |
| TMT part B – A (s) | 71.4 ± 103.6 | 43.9 ± 46.4 | 0.27 |
| HADS | 8.5 ± 5.2 | 4.2 ± 4.7 | 0.007 |
| Anxiety levels | 2.7 ± 12.0 | 0.2 ± 0.5 | < 0.001 |

Values indicate mean ± standard deviation. **p*-values of two-sample t-test. (MoCA = Montreal Cognitive Assessment (range: 0 – 30), TMT = Trail Making Test, HADS = Hospital Anxiety and Depression Scale (range 0 – 42), anxiety levels (range 0 – 21), MDS-UPDRS = Movement Disorders Society's Unified Parkinson's Disease Rating Scale (range: 0 – 132), NFOGQ = New Freezing of Gait Questionnaire (range 0 – 28))

For Review Only

# Supplementary Material

## Head angles during the various gait events

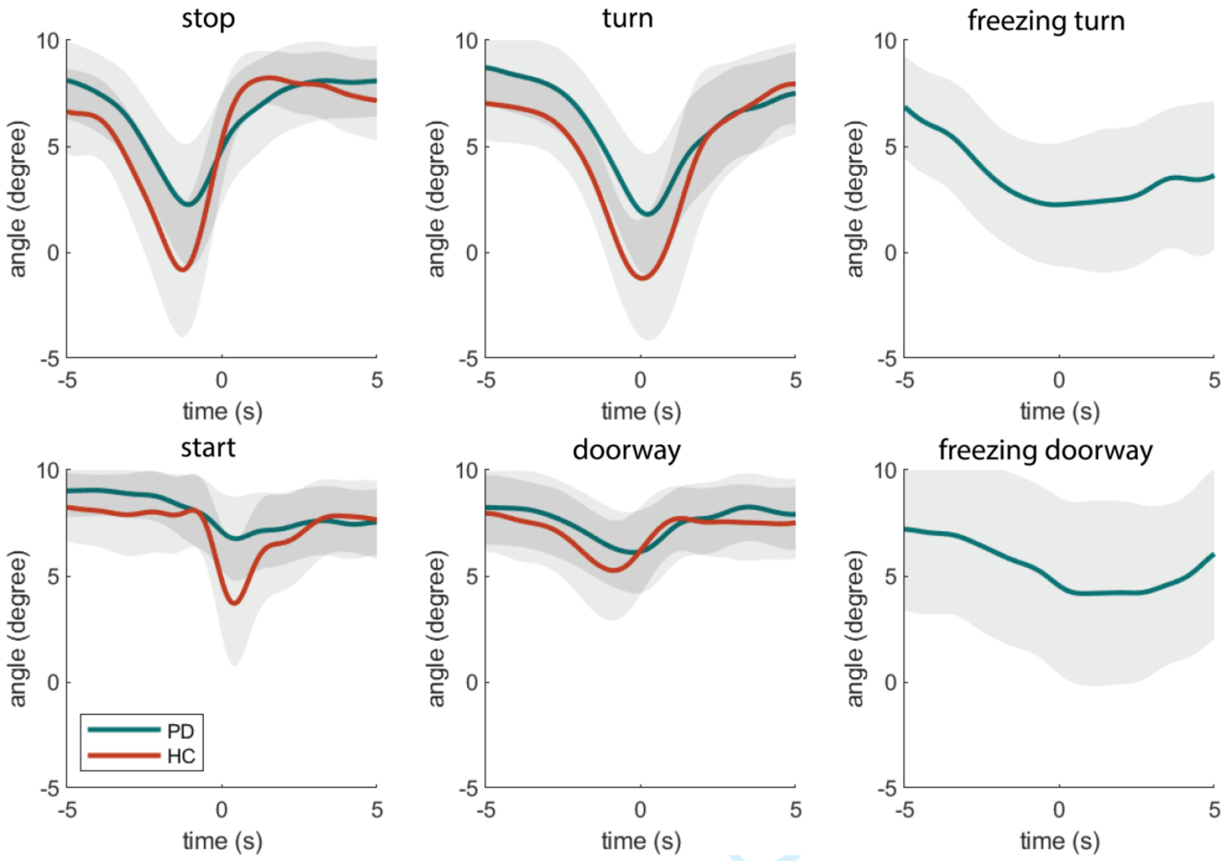


**Supplementary Fig. 1: Head tilt during various gait events.** The angles represent the angle of the head relative to the neck with a positive angle indicating an upward gaze and a negative angle indicating a downward gaze. (PD = Parkinson's disease; HC = healthy controls)

## Definitions of the successful gait events

### Start/stops

Start/stop events were defined as an increase/decrease in horizontal pelvis velocity over a threshold of 0.1 m/s after a start/stop signal was given. The pelvis velocity data was first low pass filtered with a 0.5 Hz 3rd order Butterworth filter.

## Doorway passage

The doorway passage events were determined as the crossing point of the center of mass position halfway the gait trajectory. Because position data derived from acceleration data (IMUs) can be subjective to drifts, all doorway events were checked and, if necessary, adjusted based on the video recordings.

## Turns

The begin and end of the turns were determined based on the orientation of the pelvis as follows: the orientation data of the pelvis was first converted from quaternions to Euler angles, unwrapped, and low pass filtered with a 5 Hz 3rd order Butterworth filter. The turns were then detected by finding the moments where the pelvis crossed 90 degrees. To define the begin and end of turns, we fitted a regression line before and after the turn to estimate the “normal” pelvis orientation during straight walking (Supplementary Fig. 2).[1,2] The begin and end of the turn were then calculated as the timepoints where the pelvis orientation crossed this regression line with more than 5°.

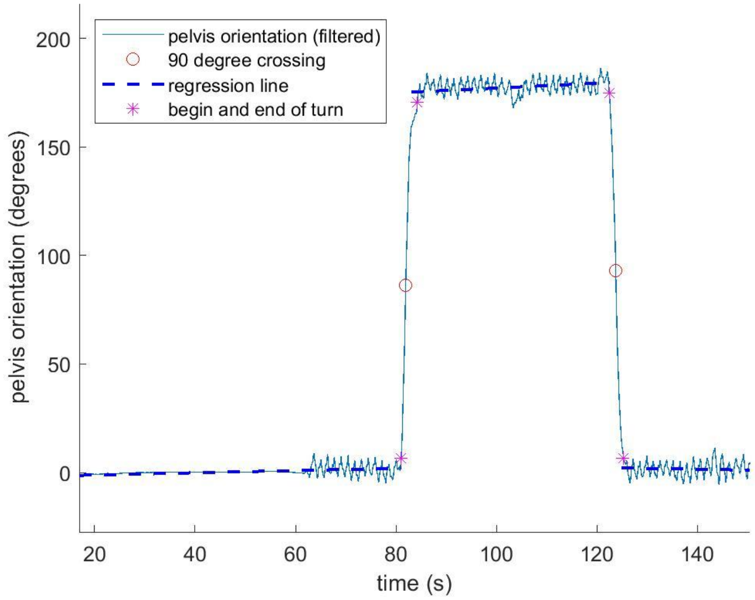


**Supplementary Fig. 2: Example of turn detection algorithm.**



For Review Only

# Onset of freezing during turning and doorway passing

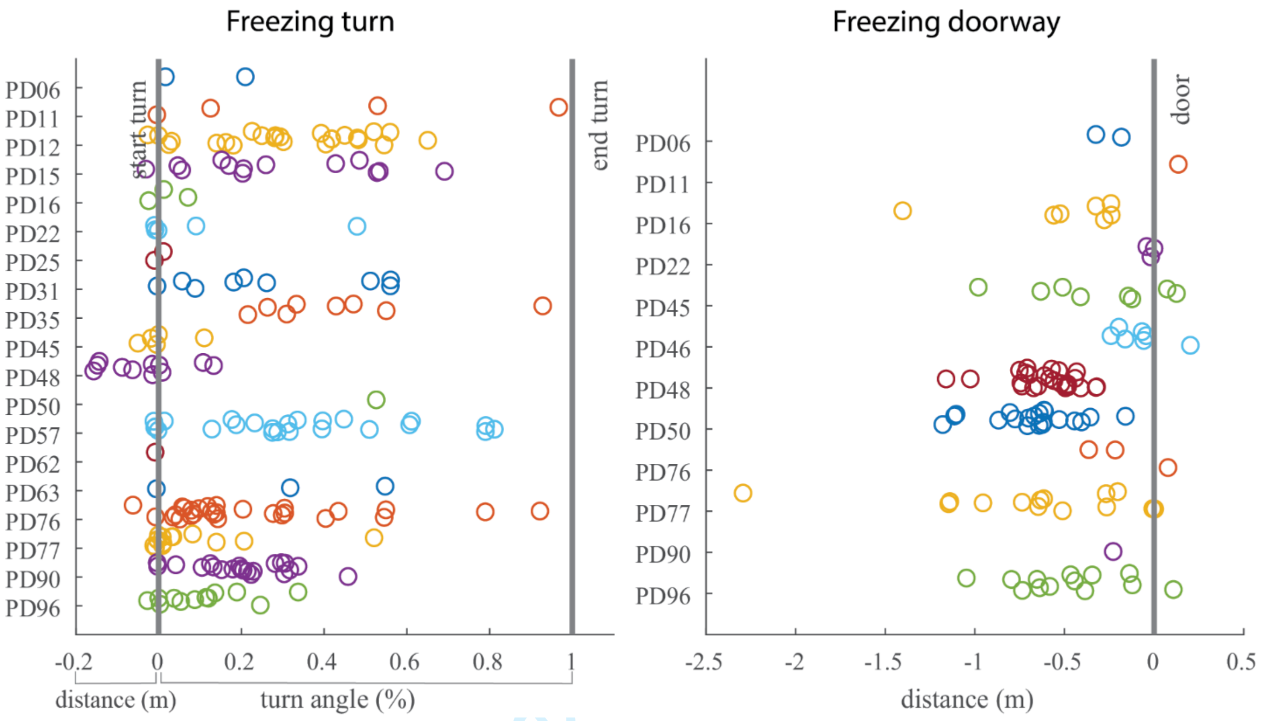


**Supplementary Fig. 3: onset of the freezing events relative to the turn/doorway passage.** Each circle represents a freezing episode and each participant is shown in a different color. The onset of the freezing events during turning is expressed in % of the turn angle with 180° corresponding to 100%. If the freezing occurred prior to the onset of the pelvis rotation, we expressed it as the distance (in m) from the onset of the pelvis rotation. The onset of the freezing at the doorway passage is expressed as the distance from the doorway (in m).

# Details of Bayesian hierarchical model fitting

All models were fitted using the packages brms[3] (version 2.18.0) in Rstudio (version 2022.07.2) using Markov chain Monte Carlo (MCMC) sampling. For each model, four chains were run with 10,000 iterations per chain of which 500 served as warm-up. We adapted the sampling behavior to avoid divergent transitions by increasing *adapt_delta* to 0.99. Convergence of the chains was checked with caterpillar plots and Gelman-Rubin statistics (Rhat in Supplementary Table 1-2). R-values were all close to 1.00. There were no divergent transitions. The effective sample sizes were all above 10,000,[4] except for the doorway condition PMC region (> 8,000). Note that for this condition we performed a subgroup analysis splitting the group in freezers and non-freezers (FOG+ vs FOG- vs HC). The effective sample sizes of this analysis were all above 10,000 (Supplementary Table 1b).

There was no previous research available to motivate an informed prior, so default flat priors from brms were used. We performed a sensitivity analysis by refitting the models starting from normal distributed priors for the fixed effects, centered at 0 and with a standard deviation of 1 (representing a z-distribution like the transformed fNIRS data). Visual comparison by superimposing the probability density functions did not reveal substantial differences between the two sets of priors.

For Review Only

**Supplementary Table 1a: details on MCMC sampling of type 1 models**

| Condition | ROI | Parameter | Rhat | ESS_ratio | ESS | n_diverg |
|---|---|---|---|---|---|---|
| start | M1 | Intercept | 1.00 | 0.70 | 26575 | 0 |
| | | Group | 1.00 | 0.66 | 25267 | 0 |
| | PMC | Intercept | 1.00 | 0.71 | 26941 | 0 |
| | | Group | 1.00 | 0.72 | 27216 | 0 |
| | SMA | Intercept | 1.00 | 0.94 | 35626 | 0 |
| | | Group | 1.00 | 0.96 | 36299 | 0 |
| | PFC | Intercept | 1.00 | 0.87 | 33105 | 0 |
| | | Group | 1.00 | 0.80 | 30287 | 0 |
| | PPC | Intercept | 1.00 | 1.04 | 39379 | 0 |
| | | Group | 1.00 | 1.16 | 43939 | 0 |
| walking | M1 | Intercept | 1.00 | 0.59 | 22566 | 0 |
| | | Group | 1.00 | 0.48 | 18229 | 0 |
| | PMC | Intercept | 1.00 | 0.75 | 28577 | 0 |
| | | Group | 1.00 | 0.77 | 29176 | 0 |
| | SMA | Intercept | 1.00 | 0.71 | 27153 | 0 |
| | | Group | 1.00 | 0.73 | 27587 | 0 |
| | PFC | Intercept | 1.00 | 0.90 | 34141 | 0 |
| | | Group | 1.00 | 0.79 | 29950 | 0 |
| | PPC | Intercept | 1.00 | 0.45 | 17070 | 0 |
| | | Group | 1.00 | 0.44 | 16691 | 0 |
| stop | M1 | Intercept | 1.00 | 0.86 | 32801 | 0 |
| | | Group | 1.00 | 0.85 | 32397 | 0 |
| | PMC | Intercept | 1.00 | 0.79 | 30191 | 0 |
| | | Group | 1.00 | 0.77 | 29427 | 0 |
| | SMA | Intercept | 1.00 | 0.65 | 24703 | 0 |
| | | Group | 1.00 | 0.61 | 23239 | 0 |
| | PFC | Intercept | 1.00 | 0.72 | 27286 | 0 |
| | | Group | 1.00 | 0.71 | 26939 | 0 |
| | PPC | Intercept | 1.00 | 0.77 | 29196 | 0 |
| | | Group | 1.00 | 0.68 | 25668 | 0 |
| standing | M1 | Intercept | 1.00 | 0.46 | 17494 | 0 |
| | | Group | 1.00 | 0.50 | 18986 | 0 |
| | PMC | Intercept | 1.00 | 0.52 | 19574 | 0 |
| | | Group | 1.00 | 0.53 | 20014 | 0 |
| | SMA | Intercept | 1.00 | 0.79 | 30147 | 0 |
| | | Group | 1.00 | 0.84 | 31909 | 0 |
| | PFC | Intercept | 1.00 | 0.62 | 23534 | 0 |
| | | Group | 1.00 | 0.61 | 23266 | 0 |
| | PPC | Intercept | 1.00 | 0.37 | 13881 | 0 |
| | | Group | 1.00 | 0.34 | 12893 | 0 |
| turn | M1 | Intercept | 1.00 | 0.61 | 23045 | 0 |
| | | Group | 1.00 | 0.63 | 24073 | 0 |
| | PMC | Intercept | 1.00 | 0.79 | 29883 | 0 |
| | | Group | 1.00 | 0.76 | 28711 | 0 |
| | SMA | Intercept | 1.00 | 0.59 | 22578 | 0 |
| | | Group | 1.00 | 0.62 | 23479 | 0 |
| | PFC | Intercept | 1.00 | 1.41 | 53601 | 0 |
| | | Group | 1.00 | 1.23 | 46648 | 0 |

For Review Only

| | | | | | | |
|---|---|---|---|---|---|---|
| | PPC | Intercept | 1.00 | 0.63 | 24066 | 0 |
| | | Group | 1.00 | 0.62 | 23482 | 0 |
| door | M1 | Intercept | 1.00 | 0.30 | 11516 | 0 |
| | | Group | 1.00 | 0.36 | 13567 | 0 |
| | PMC | Intercept | 1.00 | 0.24 | 9222 | 0 |
| | | Group | 1.00 | 0.23 | 8738 | 0 |
| | SMA | Intercept | 1.00 | 0.35 | 13413 | 0 |
| | | Group | 1.00 | 0.35 | 13271 | 0 |
| | PFC | Intercept | 1.00 | 1.20 | 45552 | 0 |
| | | Group | 1.00 | 1.08 | 40911 | 0 |
| | PPC | Intercept | 1.00 | 0.42 | 16107 | 0 |
| | | Group | 1.00 | 0.39 | 14943 | 0 |

ROI = Region of Interest; Rhat = Gelman-Rubin statistics; ESS = effective sampling size; n_diverg = number of divergent transitions

**Supplementary Table 1b: details on MCMC sampling of type 1 model with subgroup analysis (doorway passing; FOG+ vs FOG- vs HC)**

For Review Only

| Condition | ROI | Parameter | Rhat | ESS_ratio | ESS | n_diverg |
|---|---|---|---|---|---|---|
| door_subgr. | M1 | Intercept | 1.00 | 0.37 | 14097 | 0 |
| | | Group1 | 1.00 | 0.30 | 11542 | 0 |
| | | Group2 | 1.00 | 0.32 | 12073 | 0 |
| | PMC | Intercept | 1.00 | 0.30 | 11257 | 0 |
| | | Group1 | 1.00 | 0.26 | 9981 | 0 |
| | | Group2 | 1.00 | 0.27 | 10138 | 0 |
| | SMA | Intercept | 1.00 | 0.57 | 21587 | 0 |
| | | Group1 | 1.00 | 0.48 | 18199 | 0 |
| | | Group2 | 1.00 | 0.49 | 18708 | 0 |
| | PFC | Intercept | 1.00 | 0.85 | 32248 | 0 |
| | | Group1 | 1.00 | 0.83 | 31684 | 0 |
| | | Group2 | 1.00 | 0.78 | 29586 | 0 |
| | PPC | Intercept | 1.00 | 0.49 | 18485 | 0 |
| | | Group1 | 1.00 | 0.42 | 16080 | 0 |
| | | Group2 | 1.00 | 0.39 | 14933 | 0 |

ROI = Region of Interest; Rhat = Gelman-Rubin statistics; ESS = effective sampling size; n_diverg = number of divergent transitions

**Supplementary Table 2: details on MCMC sampling of type 2 models**

| Condition | ROI | Parameter | Rhat | ESS_ratio | ESS | n_diverg |
|---|---|---|---|---|---|---|
| FOGturn | M1 | Intercept | 1.00 | 0.43 | 16155 | 0 |
| | | Condition1 | 1.00 | 0.58 | 22044 | 0 |
| | | Condition2 | 1.00 | 0.71 | 27060 | 0 |
| | PMC | Intercept | 1.00 | 0.52 | 19799 | 0 |
| | | Condition1 | 1.00 | 0.93 | 35207 | 0 |
| | | Condition2 | 1.00 | 0.95 | 36086 | 0 |
| | SMA | Intercept | 1.00 | 0.51 | 19407 | 0 |
| | | Condition1 | 1.00 | 0.63 | 24032 | 0 |
| | | Condition2 | 1.00 | 0.65 | 24717 | 0 |
| | PFC | Intercept | 1.00 | 0.77 | 29414 | 0 |
| | | Condition1 | 1.00 | 0.56 | 21264 | 0 |
| | | Condition2 | 1.00 | 0.68 | 25951 | 0 |
| | PPC | Intercept | 1.00 | 0.47 | 17967 | 0 |

| | | | | | | |
|---|---|---|---|---|---|---|
| | | Condition1 | 1.00 | 0.62 | 23743 | 0 |
| | | Condition2 | 1.00 | 0.59 | 22403 | 0 |
| FOGdoor | M1 | Intercept | 1.00 | 0.43 | 16244 | 0 |
| | | Condition1 | 1.00 | 0.39 | 14729 | 0 |
| | | Condition2 | 1.00 | 0.44 | 16774 | 0 |
| | PMC | Intercept | 1.00 | 0.30 | 11501 | 0 |
| | | Condition1 | 1.00 | 0.52 | 19686 | 0 |
| | | Condition2 | 1.00 | 0.55 | 20817 | 0 |
| | SMA | Intercept | 1.00 | 0.41 | 15505 | 0 |
| | | Condition1 | 1.00 | 0.64 | 24363 | 0 |
| | | Condition2 | 1.00 | 0.73 | 27691 | 0 |
| | PFC | Intercept | 1.00 | 0.85 | 32195 | 0 |
| | | Condition1 | 1.00 | 0.75 | 28423 | 0 |
| | | Condition2 | 1.00 | 0.78 | 29819 | 0 |
| | PPC | Intercept | 1.00 | 0.41 | 15726 | 0 |
| | | Condition1 | 1.00 | 0.36 | 13830 | 0 |
| | | Condition2 | 1.00 | 0.49 | 18443 | 0 |

ROI = Region of Interest; Rhat = Gelman-Rubin statistics; ESS = effective sampling size; n_diverg = number of divergent transitions

For Review Only

# Starting & walking

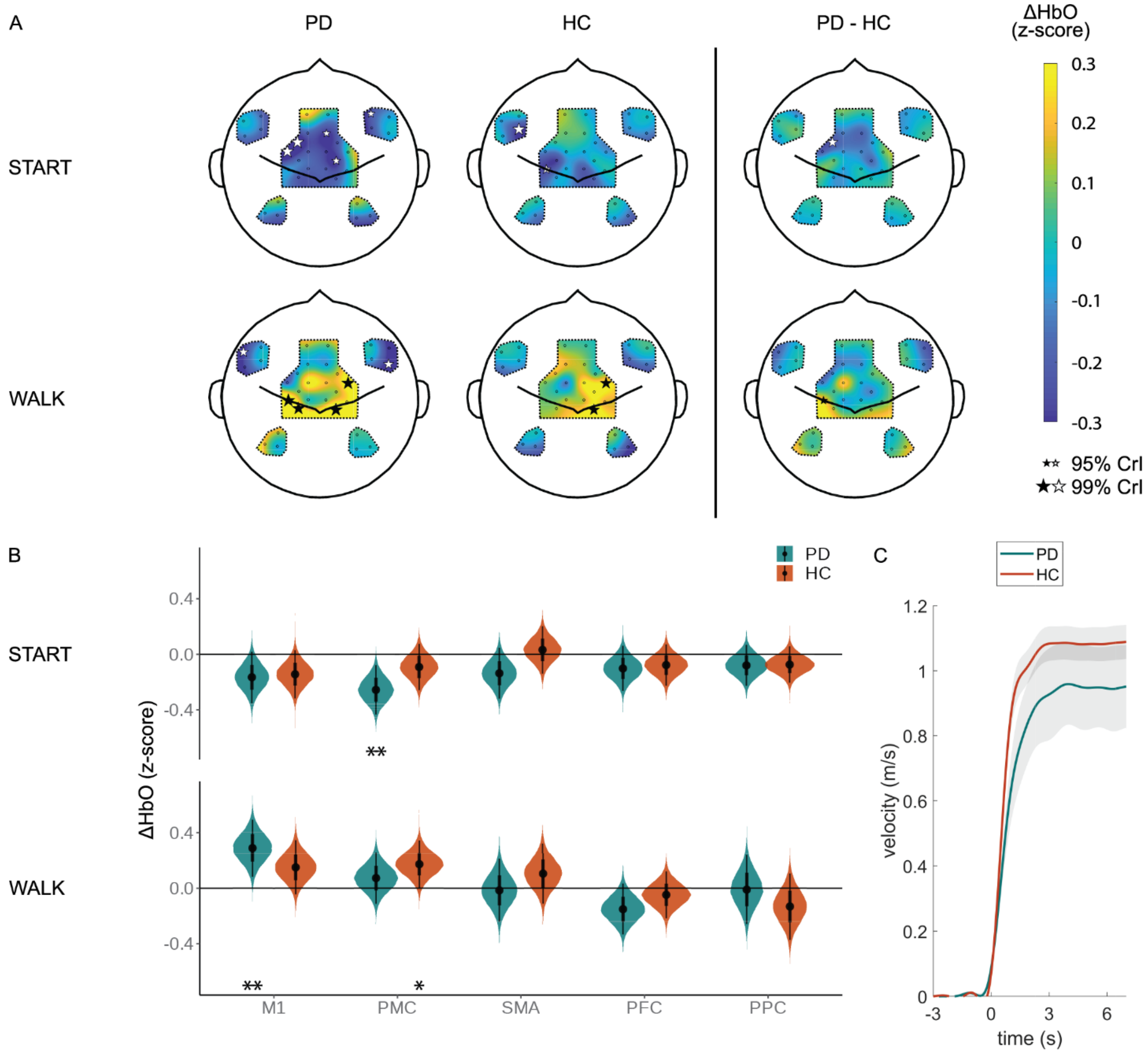


**Supplementary Fig. 4: Starting and walking.** (**A**) Cortical activity maps of estimated ΔHbO responses 0 to 3 s after starting to walk (start) and 7 to 10 s after starting to walk (walk) compared to baseline (-10 -5 s). The black and white stars indicate channels with 95% credibility interval (CrI) of the posterior probabilities excluding zero (small star) or 99% of the CrI excluding zero (large star). The size of the star scales with the probability that the estimated ΔHbO excludes zero. (**B**) Posterior probabilities of the estimated average ΔHbO responses for each ROI. The stars underneath the violin plots indicate if the posterior probability of the estimated ΔHbO response is different from baseline; the stars above the violin plots indicate if the posterior probability of the

estimated ΔHbO response differs between the groups (* = 95% CrI excluding zero; ** = 99% CrI excluding zero). **(C)** Average walking velocity for the two study groups when starting to walk (t = 0 s). The grey areas indicate the 95% confidence intervals. (PD = Parkinson's Disease group; HC = healthy control group; ΔHbO = change in oxygenated hemoglobin; CrI = credibility interval; ROI = region of interest; M1: primary motor cortex; PMC: premotor cortex; SMA: supplementary motor area; PFC: prefrontal cortex; PPC: posterior parietal cortex)

For Review Only

# Doorways analysis without subgroups for PD

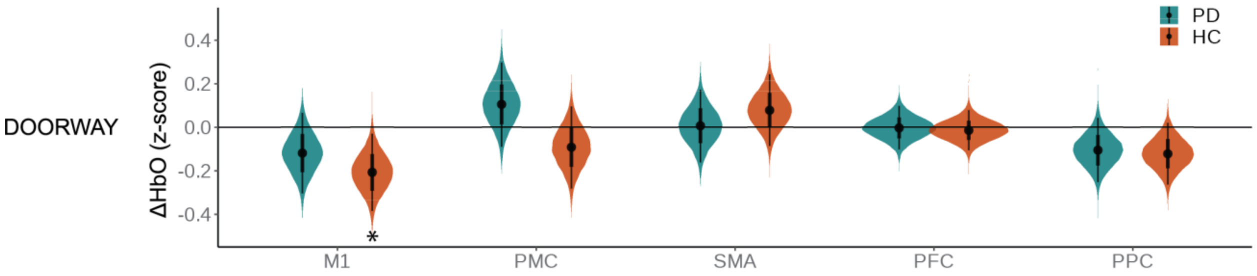


**Supplementary Fig. 5: Doorway passing (original analysis without subgroups for PD).** Posterior probabilities of the estimated average ΔHbO responses for each ROI. The stars underneath the violin plots indicate if the posterior probability of the estimated ΔHbO response is different from baseline; the stars above the violin plots indicate if the posterior probability of the estimated ΔHbO response differs between the groups (* = 95% CrI excluding zero; ** = 99% CrI excluding zero). (PD = Parkinson's Disease group; HC = healthy control group; CrI = credibility interval; ROI = region of interest; M1: primary motor cortex; PMC: premotor cortex; SMA: supplementary motor area; PFC: prefrontal cortex; PPC: posterior parietal cortex)

For Review Only

# HbO and HbR time courses

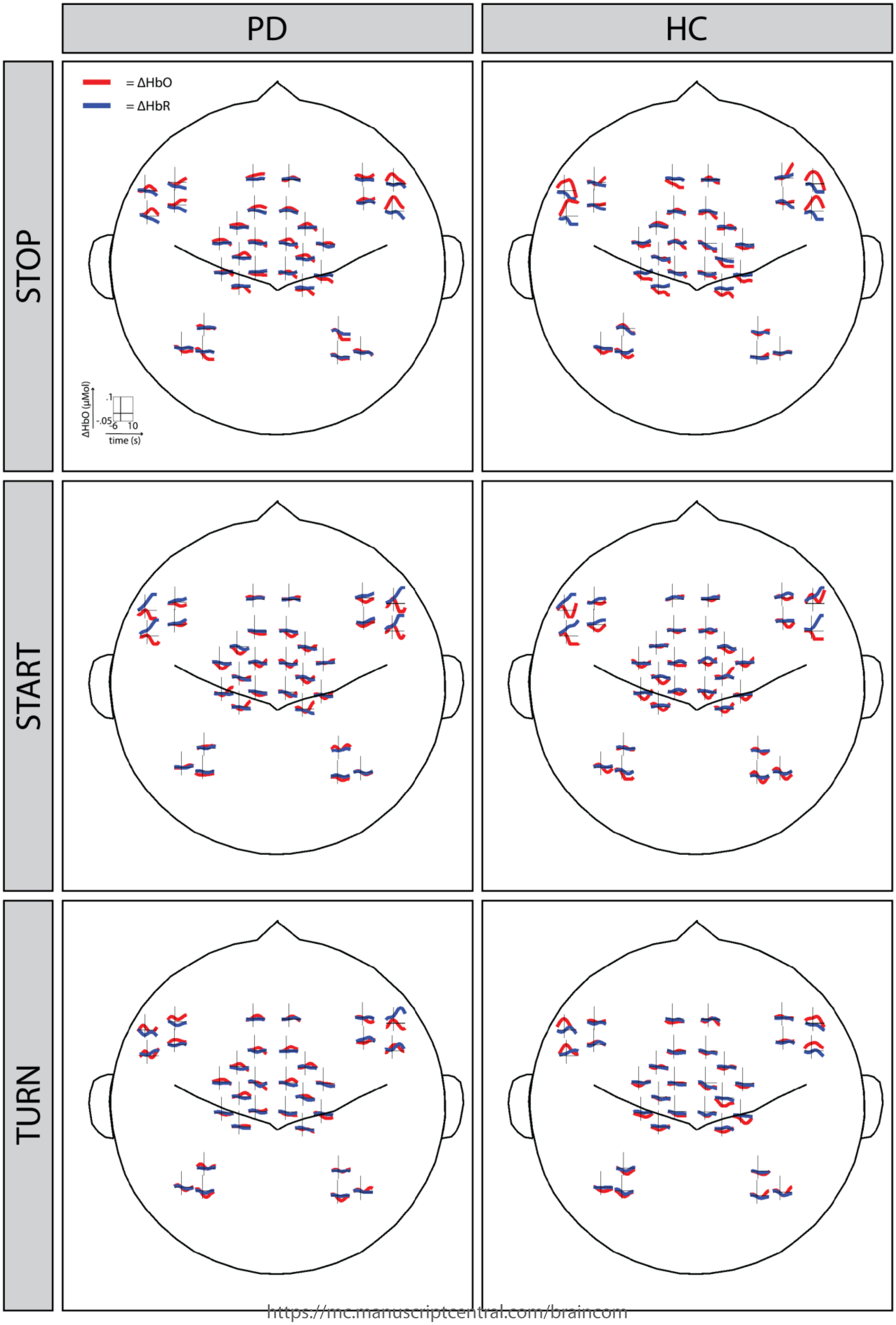

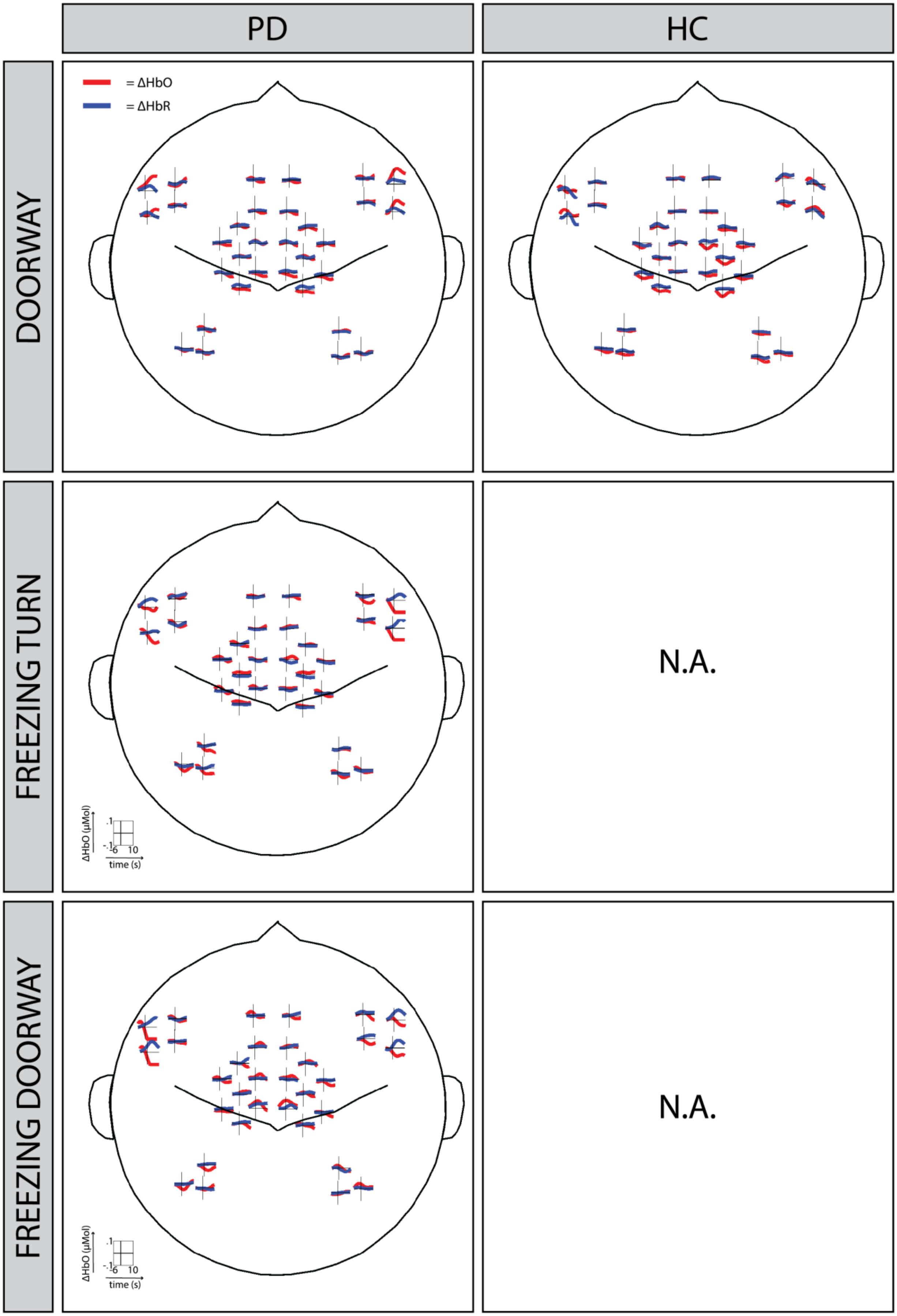
PD
HC
DOORWAY
= ΔHbO
= ΔHbR
FREEZING TURN
N.A.
ΔHbO (μMol)
.1
-.1
-6
10
time (s)
FREEZING DOORWAY
N.A.
ΔHbO (μMol)
.1
-.1
-6
10
time (s)

**Supplementary Fig. 6: Time courses of the HbO and HbR responses during the various events.** Average time course of ΔHbO (red) and ΔHbR (blue) in each channel over participants during the various conditions (rows) in the two study groups (columns). Note that we adapted the scale for the freezing conditions. (HbO = oxygenated hemoglobin, HbR = deoxygenated hemoglobin).

## MNI coordinates of the estimated channel positions

The optode positions were extracted by visualizing the 3D scans in MATLAB with the help of the FieldTrip toolbox,[5,6] and manually selecting the optode positions and anatomical landmarks (nasion, inion, left preauricular point, right preauricular point, and Cz) on the head surface. The average optode positions and anatomical landmarks were subsequently coregistered to the Colin27 brain based on the anatomical landmarks with the help of AtlasViewer.[7] Subsequently, we projected the channel positions to the cortex and calculated the Montreal Neurological Institute (MNI) coordinates of each channel and defined its underlying brain region based on automated anatomical labelling (AAL).[8]

For Review O

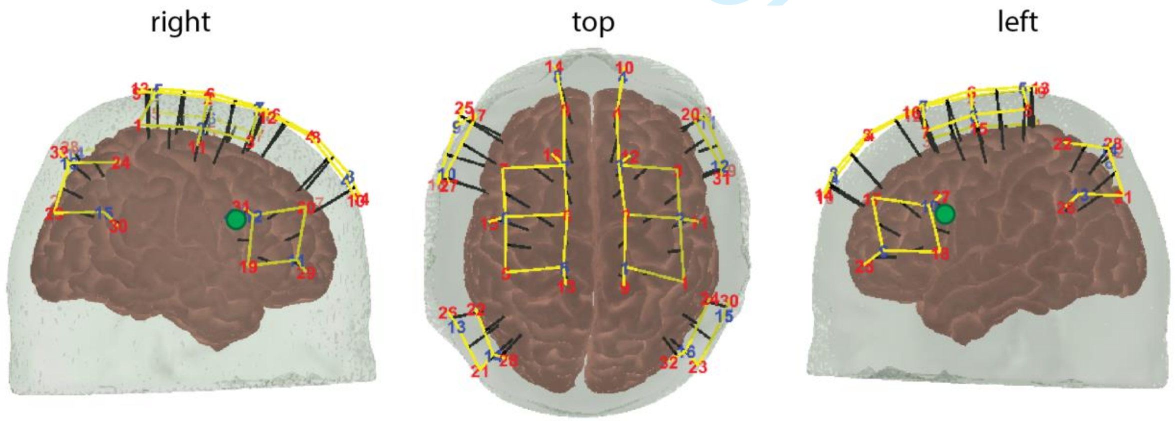


**Supplementary Fig. 7: average optode positions projected over the Colin27 brain.** The average positions of sources are indicated with red numbers, the average position of detectors with blue numbers. The yellow lines represent the channels (long and short channels). The black lines represent the projections of the channels to the cortex. The green dots correspond to the MNI

coordinates reported by Shine et al., as the center of where increased activity was observed during motor blocks in a virtual-reality fMRI paradigm.[9]

**Supplementary Table 3: MNI coordinates of estimated channel positions (long channels) and anatomical labels**

| ROI | hemisphere | source | detector | x | y | z | anatomical label (AAL) |
|---|---|---|---|---|---|---|---|
| M1 | left | 5 | 5 | -16 | -26 | 68 | Postcentral_L |
| | | 6 | 5 | -10 | -13 | 76 | Paracentral_Lobule_L |
| | | 5 | 6 | -29 | -15 | 62 | Precentral_L |
| | right | 1 | 1 | 31 | -31 | 77 | Postcentral_R |
| | | 2 | 1 | 15 | -18 | 64 | Precentral_R |
| | | 1 | 2 | 35 | -21 | 51 | Postcentral_R |
| PMC | left | 6 | 6 | -14 | -6 | 61 | Supp_Motor_Area_L |
| | | 7 | 6 | -28 | 6 | 56 | Frontal_Mid_L |
| | | 7 | 7 | -20 | 24 | 67 | Frontal_Sup_L |
| | right | 2 | 2 | 28 | -4 | 62 | Frontal_Sup_R |
| | | 3 | 2 | 36 | 2 | 47 | Precentral_R |
| | | 3 | 3 | 20 | 18 | 54 | Frontal_Sup_R |
| SMA | left | 6 | 7 | -10 | 10 | 74 | Frontal_Sup_L |
| | | 8 | 7 | -9 | 34 | 64 | Frontal_Sup_Medial_L |
| | | 8 | 8 | -9 | 55 | 43 | Frontal_Sup_Medial_L |
| | right | 2 | 3 | 14 | 14 | 75 | Supp_Motor_Area_R |
| | | 4 | 3 | 10 | 21 | 46 | Cingulum_Mid_R |
| | | 4 | 4 | 15 | 49 | 38 | Frontal_Sup_Medial_R |
| PFC | left | 17 | 9 | -47 | 45 | 16 | Frontal_Inf_Tri_L |
| | | 18 | 9 | -57 | 32 | 10 | Frontal_Inf_Tri_L |
| | | 17 | 10 | -32 | 21 | 18 | Frontal_Inf_Oper_L |
| | | 18 | 10 | -52 | 16 | 19 | Frontal_Inf_Oper_L |
| | right | 20 | 11 | 49 | 43 | 10 | Frontal_Mid_R |
| | | 19 | 11 | 64 | 36 | 1 | Frontal_Inf_Orb_R |
| | | 20 | 12 | 54 | 35 | 25 | Frontal_Mid_R |
| | | 19 | 12 | 66 | 23 | 14 | Frontal_Inf_Tri_R |
| PPC | left | 21 | 13 | -37 | -52 | 30 | Angular_L |
| | | 21 | 14 | -31 | -60 | 39 | Angular_L |
| | | 22 | 14 | -46 | -54 | 59 | Parietal_Inf_L |
| | right | 23 | 15 | 41 | -48 | 28 | Angular_R |
| | | 24 | 16 | 55 | -56 | 56 | Parietal_Inf_R |
| | | 23 | 16 | 44 | -66 | 41 | Occipital_Mid_R |

ROI = region of interest; x,y,z = Montreal Neurological Institute (MNI) coordinates; AAL = automated anatomical labeling; M1 = primary motor cortex; PMC = premotor cortex; SMA = supplementary motor cortex; PFC = prefrontal cortex; PPC = posterior parietal cortex

For Review Only

# References


1 Salarian, A. *et al.* iTUG, a sensitive and reliable measure of mobility. *IEEE Trans Neural Syst Rehabil Eng* **18**, 303-310, doi:10.1109/TNSRE.2010.2047606 (2010).

2 Miller Koop, M., Ozinga, S. J., Rosenfeldt, A. B. & Alberts, J. L. Quantifying turning behavior and gait in Parkinson's disease using mobile technology. *IBRO Rep* **5**, 10-16, doi:10.1016/j.ibror.2018.06.002 (2018).

3 Burkner, P. C. brms: An R Package for Bayesian Multilevel Models Using Stan. *J Stat Softw* **80**, 1-28, doi:10.18637/jss.v080.i01 (2017).

4 Kruschke, J. K. Bayesian Analysis Reporting Guidelines. *Nature Human Behaviour* **5**, 1282-1291, doi:10.1038/s41562-021-01177-7 (2021).

5 Homolle, S. & Oostenveld, R. Using a structured-light 3D scanner to improve EEG source modeling with more accurate electrode positions. *J Neurosci Methods* **326**, 108378, doi:10.1016/j.jneumeth.2019.108378 (2019).

6 Oostenveld, R., Fries, P., Maris, E. & Schoffelen, J. M. FieldTrip: Open source software for advanced analysis of MEG, EEG, and invasive electrophysiological data. *Comput Intell Neurosci* **2011**, 156869, doi:10.1155/2011/156869 (2011).

7 Aasted, C. M. *et al.* Anatomical guidance for functional near-infrared spectroscopy: AtlasViewer tutorial. *Neurophotonics* **2**, 020801, doi:10.1117/1.NPh.2.2.020801 (2015).

8 Tzourio-Mazoyer, N. *et al.* Automated anatomical labeling of activations in SPM using a macroscopic anatomical parcellation of the MNI MRI single-subject brain. *Neuroimage* **15**, 273-289, doi:10.1006/nimg.2001.0978 (2002).

9 Shine, J. M. *et al.* Exploring the cortical and subcortical functional magnetic resonance imaging changes associated with freezing in Parkinson's disease. *Brain* **136**, 1204-1215, doi:10.1093/brain/awt049 (2013).


For Review Only
