# Supplementary material for: Freezing of gait in Parkinson's disease is related to imbalanced stopping–related cortical activity
Source: Brain Commun. 2024 Aug 2;6(5):fcae259. doi: 10.1093/braincomms/fcae259 (PMC11369826; doi:10.1093/braincomms/fcae259)
Supplement: fcae259_Supplementary_Data [file fcae259_supplementary_data.zip › SupplementaryMaterial.pdf]

Supplementary Material

Head angles during the various gait events

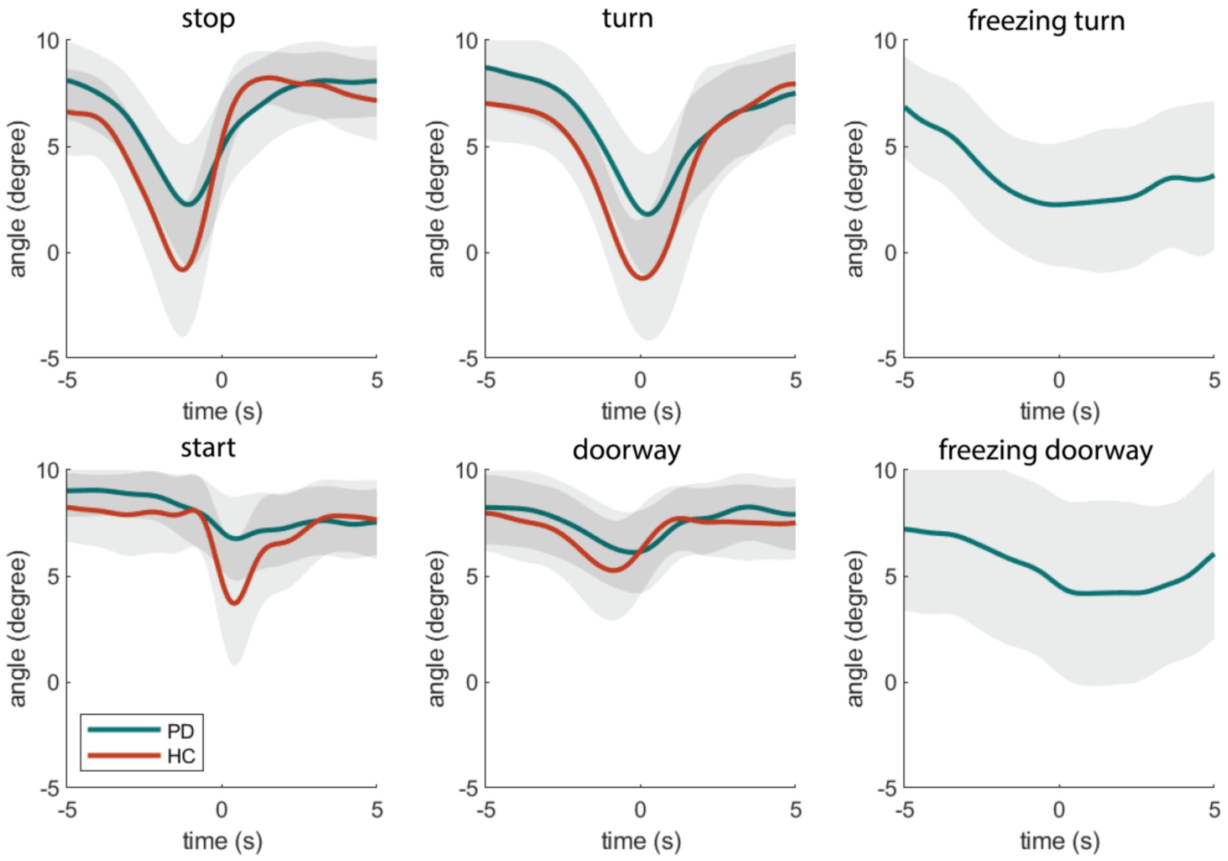

Supplementary Fig. 1: Head tilt during various gait events. The angles represent the angle of the head relative to the neck with a positive angle indicating an upward gaze and a negative angle indicating a downward gaze. The gray areas represent the 95% confidence interval (N_{PD} = 23, N_{HC} = 22) (PD = Parkinson's disease; HC = healthy controls)

Definitions of the successful gait events

Start/stops

Start/stop events were defined as an increase/decrease in horizontal pelvis velocity over a threshold of 0.1 m/s after a start/stop signal was given. The pelvis velocity data was first low pass filtered with a 0.5 Hz 3rd order Butterworth filter.

Doorway passage

The doorway passage events were determined as the crossing point of the center of mass position halfway the gait trajectory. Because position data derived from acceleration data (IMUs) can be subjective to drifts, all doorway events were checked and, if necessary, adjusted based on the video recordings.

Turns

The begin and end of the turns were determined based on the orientation of the pelvis as follows: the orientation data of the pelvis was first converted from quaternions to Euler angles, unwrapped, and low pass filtered with a 5 Hz 3rd order Butterworth filter. The turns were then detected by finding the moments where the pelvis crossed 90 degrees. To define the begin and end of turns, we fitted a regression line before and after the turn to estimate the “normal” pelvis orientation during straight walking (Supplementary Fig. 2).[1,2] The begin and end of the turn were then calculated as the timepoints where the pelvis orientation crossed this regression line with more than 5°.

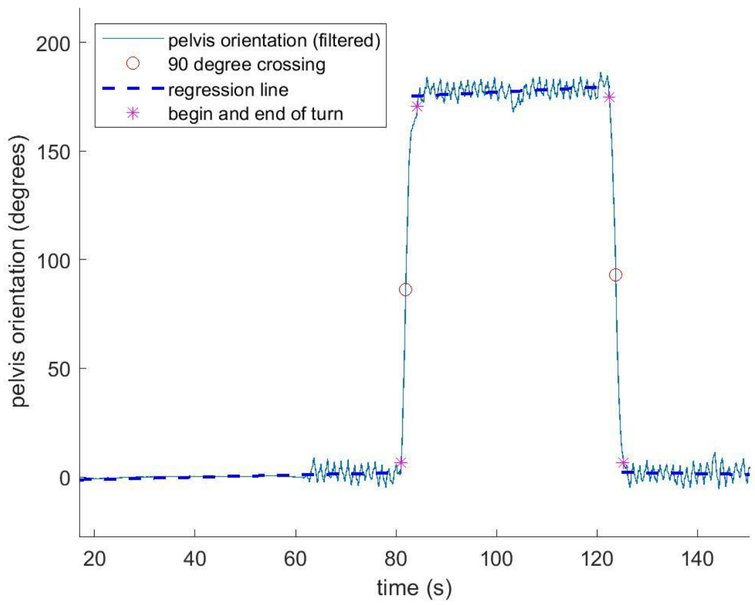

Supplementary Fig. 2: Example of turn detection algorithm. First, a turn is detected by finding the 90° crossing points of the pelvis orientation data. Subsequently, the begin and end of the turns are defined by finding the time points where the (filtered) pelvis orientation exceeds the regression line with more than 5°.

Onset of freezing during turning and doorway passing

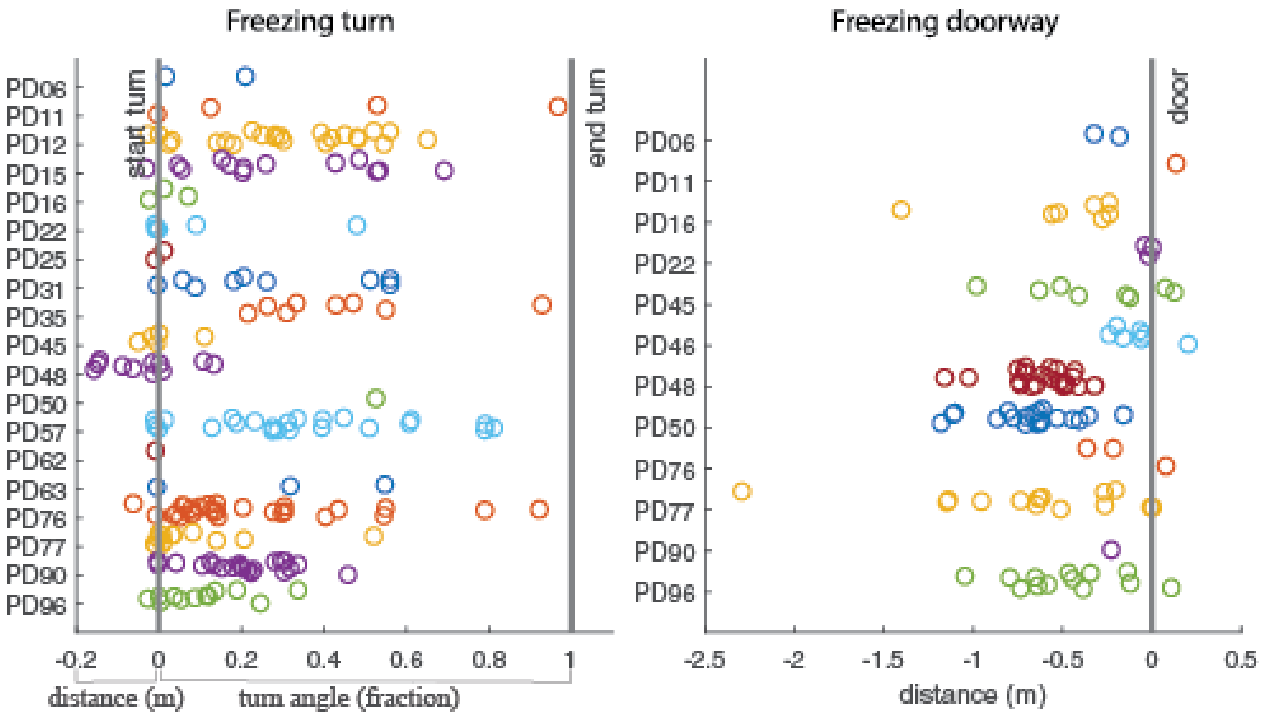

Supplementary Fig. 3: onset of the freezing events relative to the turn/doorway passage. Each circle represents a freezing episode and each participant is shown in a different color. The onset of the freezing events during turning is expressed in fraction of the turn angle with 180° corresponding to 1. If the freezing occurred prior to the onset of the pelvis rotation, we expressed it as the distance (in m) from the onset of the pelvis rotation. The onset of the freezing at the doorway passage is expressed as the distance from the doorway (in m).

Details of Bayesian hierarchical model fitting

All models were fitted using the packages brms[3] (version 2.18.0) in Rstudio (version 2022.07.2) using Markov chain Monte Carlo (MCMC) sampling. For each model, four chains were run with 10,000 iterations per chain of which 500 served as warm-up. We adapted the sampling behavior to avoid divergent transitions by increasing *adapt_delta* to 0.99. Convergence of the chains was checked with caterpillar plots and Gelman-Rubin statistics (Rhat in Supplementary Table 1-2). R-values were all close to 1.00. There were no divergent transitions. The effective sample sizes were all above 10,000,[4] except for the doorway condition PMC region (> 8,000). Note that for this condition we performed a subgroup analysis splitting the group in freezers and non-freezers (FOG+ vs FOG- vs HC). The effective sample sizes of this analysis were all above 10,000 (Supplementary Table 1b).

There was no previous research available to motivate an informed prior, so default flat priors from brms were used. We performed a sensitivity analysis by refitting the models starting from normal distributed priors for the fixed effects, centered at 0 and with a standard deviation of 1 (representing a z-distribution like the transformed fNIRS data). Visual comparison by superimposing the probability density functions did not reveal substantial differences between the two sets of priors.

Supplementary Table 1a: details on MCMC sampling of type 1 models

Condition	ROI	Parameter	Rhat	ESS_ratio	ESS	n_diverg
start	M1	Intercept	1.00	0.70	26575	0
		Group	1.00	0.66	25267	0
	PMC	Intercept	1.00	0.71	26941	0
		Group	1.00	0.72	27216	0
	SMA	Intercept	1.00	0.94	35626	0
		Group	1.00	0.96	36299	0
	PFC	Intercept	1.00	0.87	33105	0
		Group	1.00	0.80	30287	0
	PPC	Intercept	1.00	1.04	39379	0
		Group	1.00	1.16	43939	0
walking	M1	Intercept	1.00	0.59	22566	0
		Group	1.00	0.48	18229	0
	PMC	Intercept	1.00	0.75	28577	0
		Group	1.00	0.77	29176	0
	SMA	Intercept	1.00	0.71	27153	0
		Group	1.00	0.73	27587	0
	PFC	Intercept	1.00	0.90	34141	0
		Group	1.00	0.79	29950	0
	PPC	Intercept	1.00	0.45	17070	0
		Group	1.00	0.44	16691	0
stop	M1	Intercept	1.00	0.86	32801	0
		Group	1.00	0.85	32397	0
	PMC	Intercept	1.00	0.79	30191	0
		Group	1.00	0.77	29427	0
	SMA	Intercept	1.00	0.65	24703	0
		Group	1.00	0.61	23239	0
	PFC	Intercept	1.00	0.72	27286	0
		Group	1.00	0.71	26939	0
	PPC	Intercept	1.00	0.77	29196	0
		Group	1.00	0.68	25668	0
standing	M1	Intercept	1.00	0.46	17494	0
		Group	1.00	0.50	18986	0
	PMC	Intercept	1.00	0.52	19574	0
		Group	1.00	0.53	20014	0
	SMA	Intercept	1.00	0.79	30147	0
		Group	1.00	0.84	31909	0
	PFC	Intercept	1.00	0.62	23534	0
		Group	1.00	0.61	23266	0
	PPC	Intercept	1.00	0.37	13881	0
		Group	1.00	0.34	12893	0
turn	M1	Intercept	1.00	0.61	23045	0
		Group	1.00	0.63	24073	0
	PMC	Intercept	1.00	0.79	29883	0
		Group	1.00	0.76	28711	0
	SMA	Intercept	1.00	0.59	22578	0
		Group	1.00	0.62	23479	0
	PFC	Intercept	1.00	1.41	53601	0
		Group	1.00	1.23	46648	0

	PPC	Intercept	1.00	0.63	24066	0
		Group	1.00	0.62	23482	0
door	M1	Intercept	1.00	0.30	11516	0
		Group	1.00	0.36	13567	0
	PMC	Intercept	1.00	0.24	9222	0
		Group	1.00	0.23	8738	0
	SMA	Intercept	1.00	0.35	13413	0
		Group	1.00	0.35	13271	0
	PFC	Intercept	1.00	1.20	45552	0
		Group	1.00	1.08	40911	0
	PPC	Intercept	1.00	0.42	16107	0
		Group	1.00	0.39	14943	0

ROI = Region of Interest; Rhat = Gelman-Rubin statistics; ESS = effective sampling size; n_diverg = number of divergent transitions

Supplementary Table 1b: details on MCMC sampling of type 1 model with subgroup analysis (doorway passing; FOG+ vs FOG- vs HC)

Condition	ROI	Parameter	Rhat	ESS_ratio	ESS	n_diverg
door_subgr.	M1	Intercept	1.00	0.37	14097	0
		Group1	1.00	0.30	11542	0
		Group2	1.00	0.32	12073	0
	PMC	Intercept	1.00	0.30	11257	0
		Group1	1.00	0.26	9981	0
		Group2	1.00	0.27	10138	0
	SMA	Intercept	1.00	0.57	21587	0
		Group1	1.00	0.48	18199	0
		Group2	1.00	0.49	18708	0
	PFC	Intercept	1.00	0.85	32248	0
		Group1	1.00	0.83	31684	0
		Group2	1.00	0.78	29586	0
	PPC	Intercept	1.00	0.49	18485	0
		Group1	1.00	0.42	16080	0
		Group2	1.00	0.39	14933	0

ROI = Region of Interest; Rhat = Gelman-Rubin statistics; ESS = effective sampling size; n_diverg = number of divergent transitions

Supplementary Table 2: details on MCMC sampling of type 2 models

Condition	ROI	Parameter	Rhat	ESS_ratio	ESS	n_diverg
FOGturn	M1	Intercept	1.00	0.43	16155	0
		Condition1	1.00	0.58	22044	0
		Condition2	1.00	0.71	27060	0
	PMC	Intercept	1.00	0.52	19799	0
		Condition1	1.00	0.93	35207	0
		Condition2	1.00	0.95	36086	0
	SMA	Intercept	1.00	0.51	19407	0
		Condition1	1.00	0.63	24032	0
		Condition2	1.00	0.65	24717	0
	PFC	Intercept	1.00	0.77	29414	0
		Condition1	1.00	0.56	21264	0
		Condition2	1.00	0.68	25951	0
	PPC	Intercept	1.00	0.47	17967	0

		Condition1	1.00	0.62	23743	0
		Condition2	1.00	0.59	22403	0
FOGdoor	M1	Intercept	1.00	0.43	16244	0
		Condition1	1.00	0.39	14729	0
		Condition2	1.00	0.44	16774	0
	PMC	Intercept	1.00	0.30	11501	0
		Condition1	1.00	0.52	19686	0
		Condition2	1.00	0.55	20817	0
	SMA	Intercept	1.00	0.41	15505	0
		Condition1	1.00	0.64	24363	0
		Condition2	1.00	0.73	27691	0
	PFC	Intercept	1.00	0.85	32195	0
		Condition1	1.00	0.75	28423	0
		Condition2	1.00	0.78	29819	0
	PPC	Intercept	1.00	0.41	15726	0
		Condition1	1.00	0.36	13830	0
		Condition2	1.00	0.49	18443	0

ROI = Region of Interest; Rhat = Gelman-Rubin statistics; ESS = effective sampling size; n_diverg = number of divergent transitions

Starting & walking

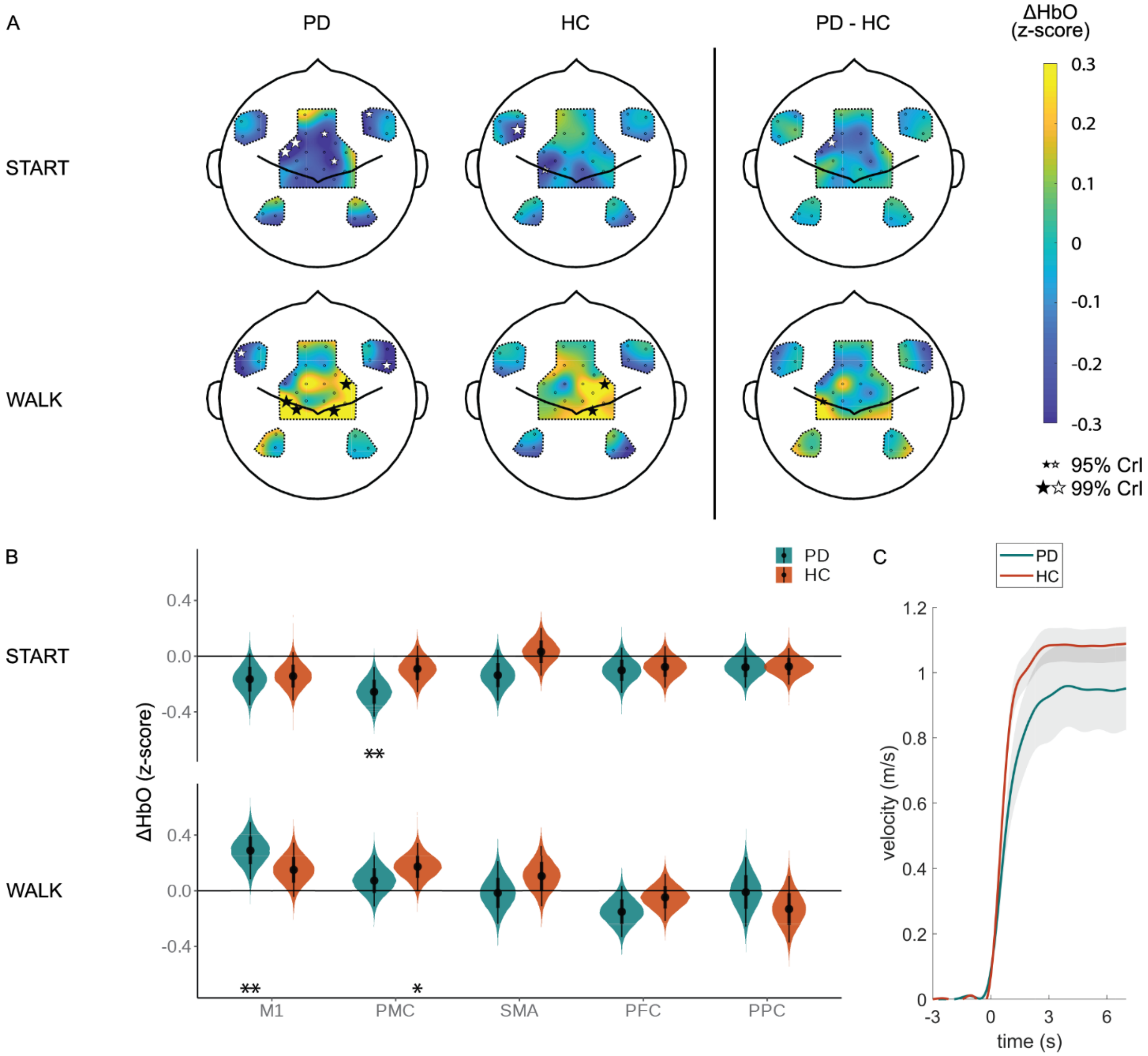

Supplementary Fig. 4: Starting and walking. (**A**) Cortical activity maps of estimated ΔHbO responses 0 to 3 s after starting to walk (start) and 7 to 10 s after starting to walk (walk) compared to baseline (-10 -5 s) as calculated by the Bayesian hierarchical model (N_{PD} = 23, N_{HC} = 22). The black and white stars indicate channels with 95% credibility interval (CrI) of the posterior probabilities excluding zero (small star) or 99% of the CrI excluding zero (large star). The size of the star scales with the probability that the estimated ΔHbO excludes zero. (**B**) Posterior probabilities of the estimated average ΔHbO responses for each ROI as calculated by the Bayesian hierarchical model (N_{PD} = 23, N_{HC} = 22). The stars underneath the violin plots indicate if the

posterior probability of the estimated ΔHbO response is different from baseline; the stars above the violin plots indicate if the posterior probability of the estimated ΔHbO response differs between the groups (* = 95% CrI excluding zero; ** = 99% CrI excluding zero). (**C)** Average walking velocity for the two study groups when starting to walk (t = 0 s). The grey areas indicate the 95% confidence intervals. (PD = Parkinson's Disease group; HC = healthy control group; ΔHbO = change in oxygenated hemoglobin; CrI = credibility interval; ROI = region of interest; M1: primary motor cortex; PMC: premotor cortex; SMA: supplementary motor area; PFC: prefrontal cortex; PPC: posterior parietal cortex)

Doorways analysis without subgroups for PD

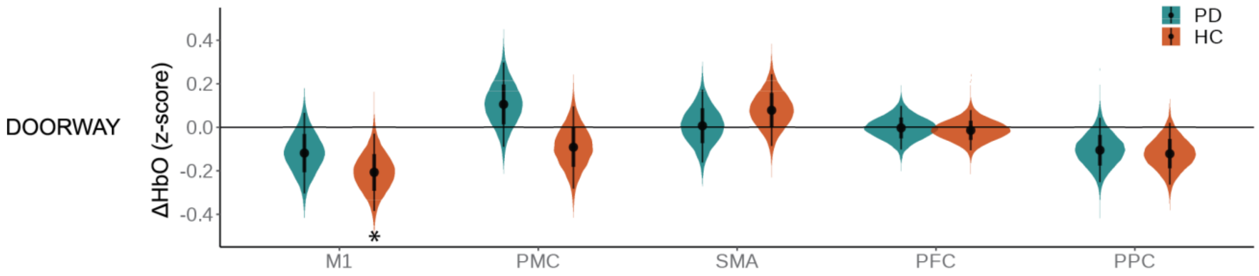

Supplementary Fig. 5: Doorway passing (original analysis without subgroups for PD). Posterior probabilities of the estimated average ΔHbO responses for each ROI as calculated by the Bayesian hierarchical model (N_{PD} = 22, N_{HC} = 22). The stars underneath the violin plots indicate if the posterior probability of the estimated ΔHbO response is different from baseline; the stars above the violin plots indicate if the posterior probability of the estimated ΔHbO response differs between the groups (* = 95% CrI excluding zero; ** = 99% CrI excluding zero). (PD = Parkinson's Disease group; HC = healthy control group; CrI = credibility interval; ROI = region of interest; M1: primary motor cortex; PMC: premotor cortex; SMA: supplementary motor area; PFC: prefrontal cortex; PPC: posterior parietal cortex)

HbO and HbR time courses

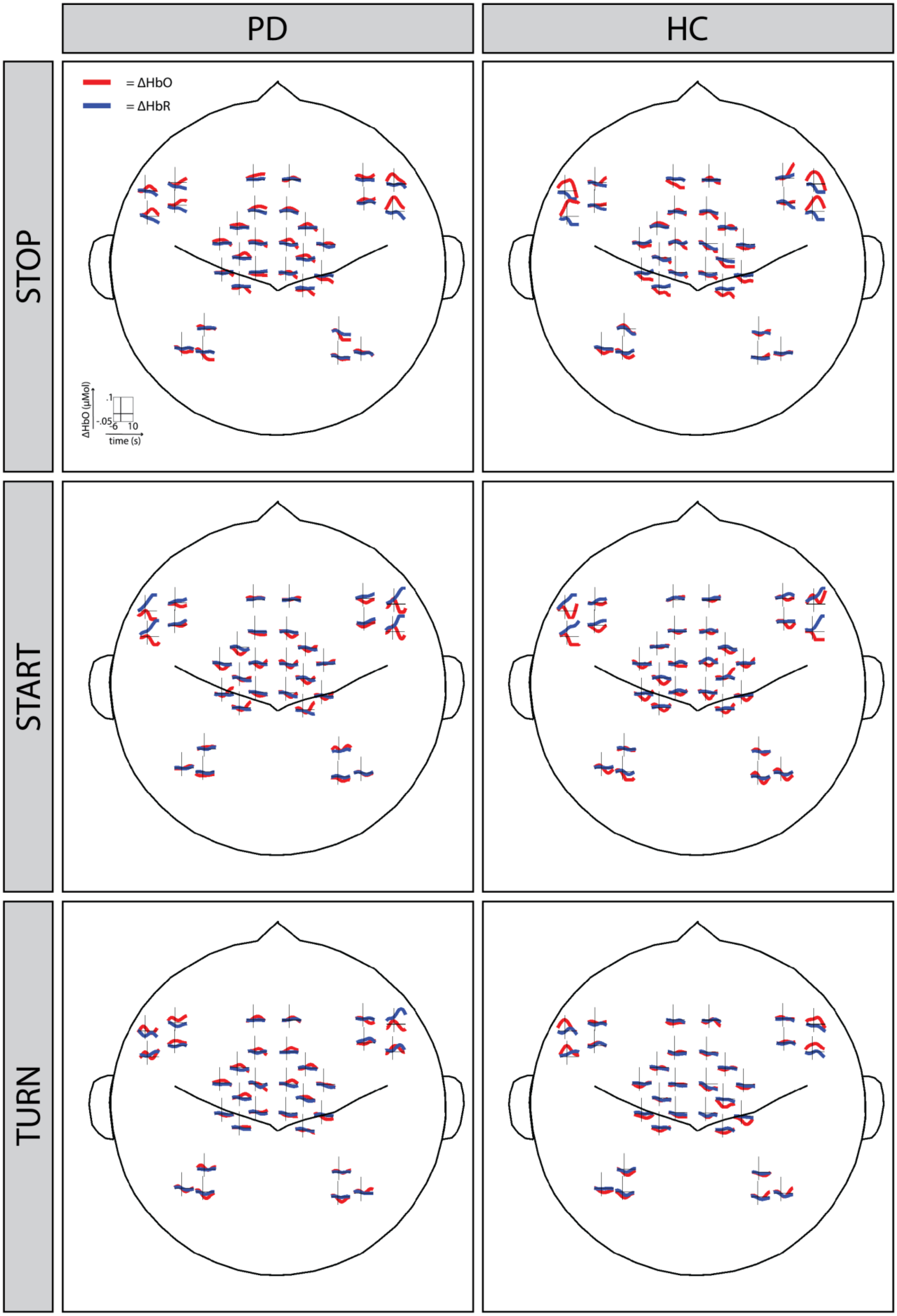

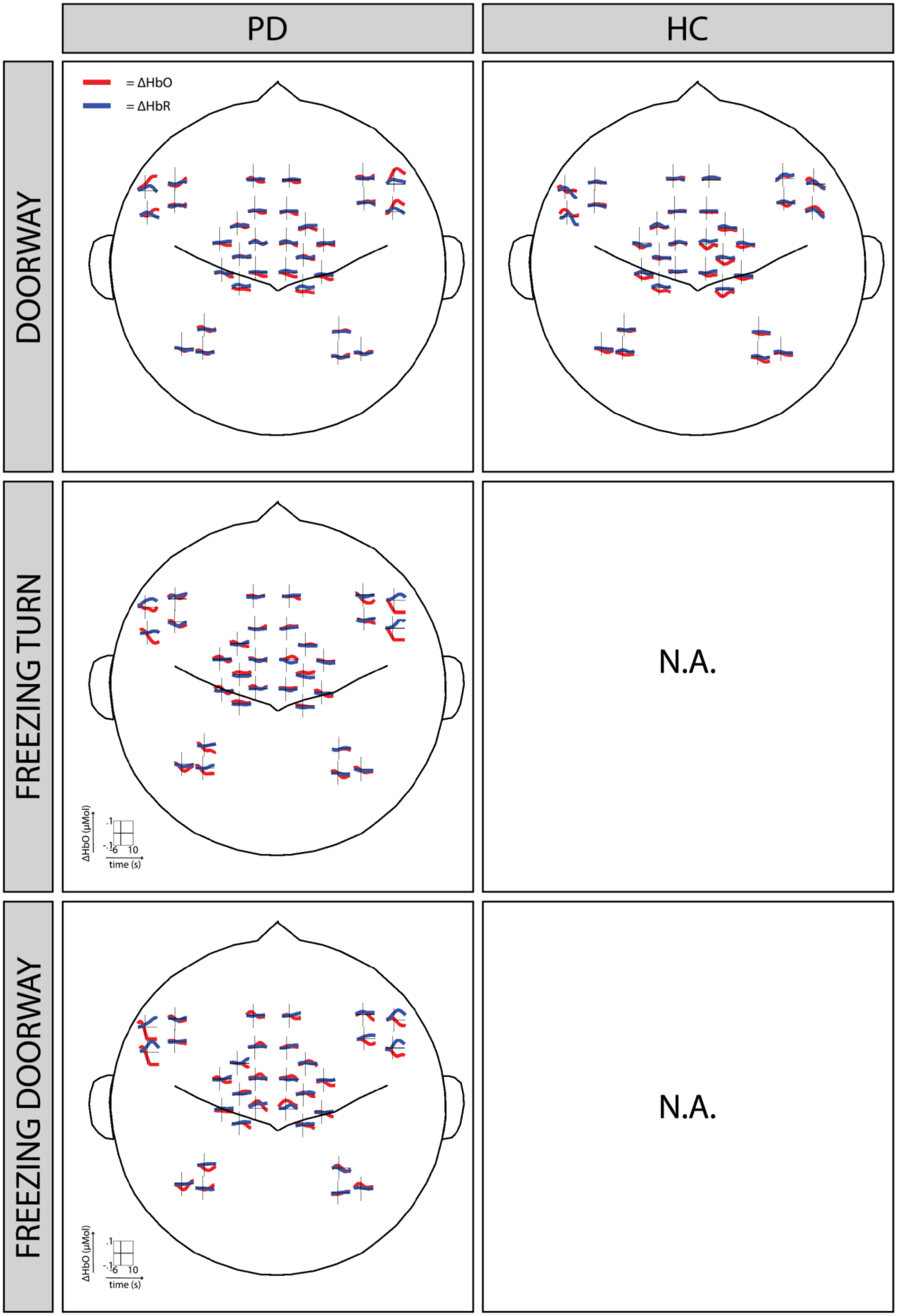

PD
HC
DOORWAY
FREEZING TURN
FREEZING DOORWAY
= ΔHbO
= ΔHbR
N.A.
N.A.
ΔHbO (μMol)
.1
-.1
-6
10
time (s)

Supplementary Fig. 6: Time courses of the HbO and HbR responses during the various events. Average time course of ΔHbO (red) and ΔHbR (blue) in each channel over participants during the various conditions (rows) in the two study groups (columns). Note that we adapted the scale for the freezing conditions. (HbO = oxygenated hemoglobin, HbR = deoxygenated hemoglobin).

MNI coordinates of the estimated channel positions

The optode positions were extracted by visualizing the 3D scans in MATLAB with the help of the FieldTrip toolbox,[5,6] and manually selecting the optode positions and anatomical landmarks (nasion, inion, left preauricular point, right preauricular point, and Cz) on the head surface. The average optode positions and anatomical landmarks were subsequently coregistered to the Colin27 brain based on the anatomical landmarks with the help of AtlasViewer.[7] Subsequently, we projected the channel positions to the cortex and calculated the Montreal Neurological Institute (MNI) coordinates of each channel and defined its underlying brain region based on automated anatomical labelling (AAL).[8]

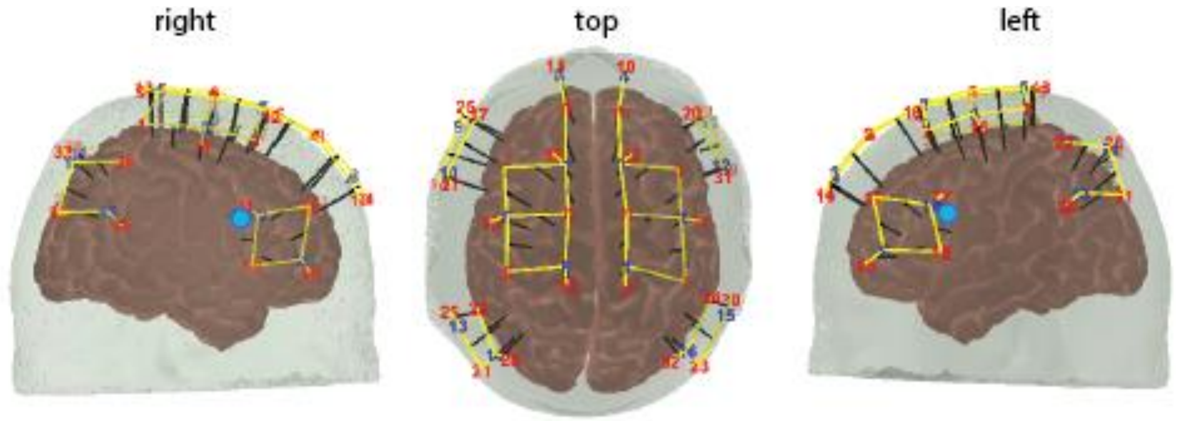

Supplementary Fig. 7: average optode positions projected over the Colin27 brain. The average positions of sources are indicated with red numbers, the average position of detectors with blue numbers. The yellow lines represent the channels (long and short channels). The black lines represent the projections of the channels to the cortex. The blue dots correspond to the MNI

coordinates reported by Shine et al., as the center of where increased activity was observed during motor blocks in a virtual-reality fMRI paradigm.[9]

Supplementary Table 3: MNI coordinates of estimated channel positions (long channels) and anatomical labels

ROI	hemisphere	source	detector	x	y	z	anatomical label (AAL)
M1	left	5	5	-16	-26	68	Postcentral_L
		6	5	-10	-13	76	Paracentral_Lobule_L
		5	6	-29	-15	62	Precentral_L
	right	1	1	31	-31	77	Postcentral_R
		2	1	15	-18	64	Precentral_R
		1	2	35	-21	51	Postcentral_R
PMC	left	6	6	-14	-6	61	Supp_Motor_Area_L
		7	6	-28	6	56	Frontal_Mid_L
		7	7	-20	24	67	Frontal_Sup_L
	right	2	2	28	-4	62	Frontal_Sup_R
		3	2	36	2	47	Precentral_R
		3	3	20	18	54	Frontal_Sup_R
SMA	left	6	7	-10	10	74	Frontal_Sup_L
		8	7	-9	34	64	Frontal_Sup_Medial_L
		8	8	-9	55	43	Frontal_Sup_Medial_L
	right	2	3	14	14	75	Supp_Motor_Area_R
		4	3	10	21	46	Cingulum_Mid_R
		4	4	15	49	38	Frontal_Sup_Medial_R
PFC	left	17	9	-47	45	16	Frontal_Inf_Tri_L
		18	9	-57	32	10	Frontal_Inf_Tri_L
		17	10	-32	21	18	Frontal_Inf_Oper_L
		18	10	-52	16	19	Frontal_Inf_Oper_L
	right	20	11	49	43	10	Frontal_Mid_R
		19	11	64	36	1	Frontal_Inf_Orb_R
		20	12	54	35	25	Frontal_Mid_R
		19	12	66	23	14	Frontal_Inf_Tri_R
PPC	left	21	13	-37	-52	30	Angular_L
		21	14	-31	-60	39	Angular_L
		22	14	-46	-54	59	Parietal_Inf_L
	right	23	15	41	-48	28	Angular_R
		24	16	55	-56	56	Parietal_Inf_R
		23	16	44	-66	41	Occipital_Mid_R

ROI = region of interest; x,y,z = Montreal Neurological Institute (MNI) coordinates; AAL = automated anatomical labeling; M1 = primary motor cortex; PMC = premotor cortex; SMA = supplementary motor cortex; PFC = prefrontal cortex; PPC = posterior parietal cortex

References

1 Salarian, A. *et al.* iTUG, a sensitive and reliable measure of mobility. *IEEE Trans Neural Syst Rehabil Eng* **18**, 303-310, doi:10.1109/TNSRE.2010.2047606 (2010).

2 Miller Koop, M., Ozinga, S. J., Rosenfeldt, A. B. & Alberts, J. L. Quantifying turning behavior and gait in Parkinson's disease using mobile technology. *IBRO Rep* **5**, 10-16, doi:10.1016/j.ibror.2018.06.002 (2018).

3 Burkner, P. C. brms: An R Package for Bayesian Multilevel Models Using Stan. *J Stat Softw* **80**, 1-28, doi:10.18637/jss.v080.i01 (2017).

4 Kruschke, J. K. Bayesian Analysis Reporting Guidelines. *Nature Human Behaviour* **5**, 1282-1291, doi:10.1038/s41562-021-01177-7 (2021).

5 Homolle, S. & Oostenveld, R. Using a structured-light 3D scanner to improve EEG source modeling with more accurate electrode positions. *J Neurosci Methods* **326**, 108378, doi:10.1016/j.jneumeth.2019.108378 (2019).

6 Oostenveld, R., Fries, P., Maris, E. & Schoffelen, J. M. FieldTrip: Open source software for advanced analysis of MEG, EEG, and invasive electrophysiological data. *Comput Intell Neurosci* **2011**, 156869, doi:10.1155/2011/156869 (2011).

7 Aasted, C. M. *et al.* Anatomical guidance for functional near-infrared spectroscopy: AtlasViewer tutorial. *Neurophotonics* **2**, 020801, doi:10.1117/1.NPh.2.2.020801 (2015).

8 Tzourio-Mazoyer, N. *et al.* Automated anatomical labeling of activations in SPM using a macroscopic anatomical parcellation of the MNI MRI single-subject brain. *Neuroimage* **15**, 273-289, doi:10.1006/nimg.2001.0978 (2002).

9 Shine, J. M. *et al.* Exploring the cortical and subcortical functional magnetic resonance imaging changes associated with freezing in Parkinson's disease. *Brain* **136**, 1204-1215, doi:10.1093/brain/awt049 (2013).
