# Supplementary material for: Freezing of gait in Parkinson's disease is related to imbalanced stopping–related cortical activity
Source: Brain Commun. 2024 Aug 2;6(5):fcae259. doi: 10.1093/braincomms/fcae259 (PMC11369826; doi:10.1093/braincomms/fcae259)
Supplement: fcae259_Supplementary_Data [file Original_submission_decision_letter .pdf › Original_submission_decision_letter .pdf]

Decision Letter One

18-Mar-2024

Dear Mrs. Cockx,

Thank you for your recent submission BRAINCOM-2023-473 entitled "Freezing of gait in Parkinson’s disease is related to imbalanced stopping-related cortical activity" to Brain Communications.

Your manuscript has been reviewed, and while the reviewers had largely positive comments, they have some constructive critiques that I think you could address in a minor revision of the manuscript. Therefore, I invite you to respond to the reviewer(s)' comments and revise your manuscript. The reviewer(s)’ comments are included at the bottom of this message.

In addition to the reviewers' comments, please also address our Scientific Editor's comments added below to comply with our requirements for presentation of scientific data at Brain Communications.

Please format your revised manuscript according to our Instructions to Authors which can be found at: https://academic.oup.com/braincomms/pages/General_Instructions
A summary of key points is attached to help with this. Please note that there can be substantial delays to publication if you don't resubmit in line with our requirements.

To revise your manuscript, click the link below or log into https://mc.manuscriptcentral.com/braincom and enter your Author Centre, where you will find your manuscript title listed under "Manuscripts with Decisions." Under "Actions," click on "Create a Revision." Your manuscript number will be appended to denote a revision.

*** PLEASE NOTE: This is a two-step process. After clicking on the link, you will be directed to a webpage to confirm. ***

https://mc.manuscriptcentral.com/braincom?URL_MASK=94d22becad324878be0e2dc4c7f33b3c

Please upload a revised manuscript with the changes you have made shown either in coloured text or using "track changes".

When submitting your revised manuscript, you will be asked to respond to the reviewer(s) comments. Please be as specific as possible in your response to all of the comments.

The editorial team at Brain Communications also aims to increase transparency in scientific publishing and to demonstrate the value that peer review brings to the scientific process. Please therefore consider whether you are willing to have reviewer comments on your paper and your response published as supplementary data with the paper, if it is accepted. This is entirely optional and will only be done if both the authors and reviewers agree. When submitting your revision you will have the opportunity to update the answer you entered previously.

IMPORTANT: Your original files are available to you when you upload your revised manuscript. Please delete any redundant files before completing the submission.

Because we are trying to facilitate timely publication of manuscripts submitted to Brain Communications, we would appreciate your revised manuscript as soon as possible. If it is not possible for you to resubmit within 3 months, the option to submit a revision will expire, and we would then consider your revised paper as a new submission. However, if you need a little more time, please get in touch and we can extend this deadline for you.

Once again, thank you for submitting your manuscript to Brain Communications and I look forward to receiving your revision.

Best wishes

Dr. Alberto Lleo
Associate Editor, Brain Communications

Professor Tara Spires-Jones
Founding Editor, Brain Communications

Reviewer(s)' Comments to Author:

Reviewer: 1

Comments to the Author

This study compared fNIRS-related activity in the prefrontal (PFC), supplementary motor area (SMA), pre-motor cortex (PMC), primary motor cortex and posterior parietal cortex in 23 people with Parkinson's disease (PD) experiencing daily freezing to 22 healthy age and sex-matched controls, during a stopping task and freezing-provoking tasks of passing through a doorway and turning. An additional aim was to compare brain activity in these regions of interest during stopping and during the freezing tasks when they did and did not induce freezing.

The findings showed that with PD presented more SMA and PMC activity during the freezing-provoking tasks compared with controls; in both group, the SMA and PFC activity during the freezing-provoking tasks was correlated to that during stopping. During freezing episodes, SMA activity was similar to that during stopping while PFC activity was lower, suggesting that a mismatch in SMA and PFC activity could contribute to freezing of gait.

I enjoyed reading this manuscript which presented a comprehensive study of potential mechanisms underlying freezing of gait in people with Parkinson's disease. It extended the previous work in that area which had mostly been limited to the study of the PFC and presented balanced arguments throughout the discussion of the results. It concluded with an interesting hypothesis of freezing of gait being related to failure of the PFC, in particular the inferior frontal gyrus, to compensate for excessive SMA activity. The study used rigorous methodology, in particular robust processing pipelines and processes for the fNIRS data. There is an abundance of nice figures presenting the results. I only have minor comments. Please see below.

In reading the study, i felt that the experiment was missing a group of people with PD non-freezers as an intermediate group. I see that the authors acknowledge this limitation (lines 479-482).

Introduction
Line 103. What were the hypotheses a-priori?

Methods
Line 268. A brief statement of how freezing of gait was defined would be useful rather than just referring the reader to the Gilat reference. What happened after a freezing episode? Did the participants continue the trial?
Line 276. Did some participants lack control data for the freezing-provoking tasks because of consistently freezing during these tasks? If so, how many?
Line 297. How were these regions of interest computed? Average of the channels as per supplementary Table 3?
Did the authors look at hemispheric differences?

Results
Table 1.
I am surprised to see that there was no statistically significant difference in the Trails-making tests between the PD and Healthy control groups.
What does the % time frozen refers to?

Reviewer: 2 – Houyou Fan

Comments to the Author

Dear authors!

Your fNIRS data of 23 PD patients and 22HCs showing that – cortical activity during stopping involving activation of the SMA and prefrontal cortex (PFC), and activity in the PFC during actual freezing events was lower than during voluntary stopping. During these freezing-provoking tasks, the freezers displayed higher activity in the premotor areas than controls. So these findings merit publication.
I have a small question about this article.

Why not differentiate between OFF or ON state freezing as an inclusion criterium? As u mentioned, the participants with PD performed all procedures in the OFF state. Will the outcome be different if the

patients are OFF or ON -freezing patients? Considering the limited number of patients, a further analysis of specific freezing type is needed to get unbiased outcomes.

Comments from the Scientific Editorial team:

-Please add an Abbreviated Summary (up to 50 words) of your paper that captures the main purpose and conclusions of the work. The summary should state the main results written in the third person ('[1st author surname] et al. report that …') and conclusion. This summary will be used in the contents list online and should not contain any abbreviations other than those on the accepted abbreviations list and accepted gene/protein names, making it accessible to the non-specialist. Please upload it as a Word document. Allowed abbreviations: AIDS; ANOVA; AMPA; ATP; A,T,C,G; CNS; CSF; CT; DNA; ECG; EEG; EMG; GABA; HIV; MRI; NMDA; PET; PCR; RNA.

-Please provide a graphical abstract. A graphical abstract is a visual representation of the central finding or methodology of the paper. It will be displayed in the table of contents. Authors should strive to make the graphical abstract informative, interesting, visually appealing, and straightforward (no caption needed, no abbreviations except gene names). The technical requirements for the graphical abstracts are:

- Size: 120 x 120 mm (300-600dpi) or 3000 x 3000 pixels
- Font: Arial 12-16 pt
- File type: tiff or PDF
- Aspect ratio: 1:1 preferred

-Figure 2A, Supplementary figures 3, 7: Please revisit your colour choice in this figure as the green-red combination is not colour-blind friendly.

-Figures 3, 5 and Box 1, Supplementary figures 4, 5: For each statistical analysis part of a figure, please report in the figure legend the statistical test used. Please give details of the sample size (N=) used in the statistical test, and if the number is <10 show the single data-points. Please note: We do not accept analyses where individual measurements are the experimental unit when there is likely to be biological variability between animals, culture replicates, etc. For this type of data, we require either that a mean or median is taken per animal/culture replicate followed by statistical analyses, or that more sophisticated analyses are performed such as mixed effects modelling which can account for random effects.

-Figure 4: For each statistical analysis part of a figure, please report in the figure legend the statistical test used.

-Supplementary figure 1: For each statistical analysis part of a figure, please report in the figure legend the statistical test used. Please give details of the sample size (N=) used in the statistical test, and relevant test statistic values alongside p values (for example F or t values for ANOVAs and t-tests).

-Supplementary figure 2: Please provide a figure legend so that the figure may be easily interpreted as stand-alone figures.

-Supplementary figures 3, 6: For each statistical analysis part of a figure, please give details of the sample size (N=) used.

-At the revision stage, if you plan to edit the current figures and tables, or to make new figures and tables, please make sure that these items fit a typeset journal page (A4).
