# Supplementary material for: Freezing of gait in Parkinson's disease is related to imbalanced stopping–related cortical activity
Source: Brain Commun. 2024 Aug 2;6(5):fcae259. doi: 10.1093/braincomms/fcae259 (PMC11369826; doi:10.1093/braincomms/fcae259)
Supplement: fcae259_Supplementary_Data [file Original_submission_Response_to_reviewers_comments.pdf › Original_submission_Response_to_reviewers_comments.pdf]

Response to Reviewers

Reviewer: 1

Comments to the Author

This study compared fNIRS-related activity in the prefrontal (PFC), supplementary motor area (SMA), pre-motor cortex (PMC), primary motor cortex and posterior parietal cortex in 23 people with Parkinson's disease (PD) experiencing daily freezing to 22 healthy age and sex-matched controls, during a stopping task and freezing-provoking tasks of passing through a doorway and turning. An additional aim was to compare brain activity in these regions of interest during stopping and during the freezing tasks when they did and did not induce freezing.

The findings showed that with PD presented more SMA and PMC activity during the freezing-provoking tasks compared with controls; in both group, the SMA and PFC activity during the freezing-provoking tasks was correlated to that during stopping. During freezing episodes, SMA activity was similar to that during stopping while PFC activity was lower, suggesting that a mismatch in SMA and PFC activity could contribute to freezing of gait.

I enjoyed reading this manuscript which presented a comprehensive study of potential mechanisms underlying freezing of gait in people with Parkinson's disease. It extended the previous work in that area which had mostly been limited to the study of the PFC and presented balanced arguments throughout the discussion of the results. It concluded with an interesting hypothesis of freezing of gait being related to failure of the PFC, in particular the inferior frontal gyrus, to compensate for excessive SMA activity. The study used rigorous methodology, in particular robust processing pipelines and processes for the fNIRS data. There is an abundance of nice figures presenting the results. I only have minor comments. Please see below.

In reading the study, i felt that the experiment was missing a group of people with PD non-freezers as an intermediate group. I see that the authors acknowledge this limitation (lines 479-482).

Introduction

Line 103. What were the hypotheses a-priori?

We have added our hypotheses to the last paragraph of the introduction:

> **Line 106-116:** The goal of this work was two-fold. We first investigated whether people with Parkinson's who experience freezing ("freezers") recruited different cortical areas than age-matched controls when voluntarily stopping and when successfully (i.e., without freezing) performing gait tasks involving changes of motor programs: making 180° turns, passing through a doorway, and starting to walk. We hypothesized that the people with Parkinson's would display increased activity in higher-order cortical areas (such as the SMA, PMC, PFC and PPC) than the controls to compensate for their loss of automatic motor control.[31-33] Second, within the group of freezers, we examined which cortical areas showed freezing-related activity and how their activity differed from stops and from the same gait events without freezing (e.g., successful turns and doorway passages). Based on a previous fNIRS[27] and fMRI[34] study, we expected to observe *higher* activity in the PFC during freezing than during the other gait events.

Moreover, we also refer to these hypotheses in the discussion:

> **Line 440-442:** We compared cortical activity of people with freezing to age-matched healthy controls and – in line with our hypothesis – we observed higher and more widespread activity in the premotor areas during turning and gait termination.

Line 445-447: Although this suggests that freezing is related to stopping, freezing was different from stopping by having *lower* PFC activity. Note that this lower PFC activity is opposite from what was hypothesized from previous literature.

Line 526-527: An important note is that the relatively low PFC (IFG) activity during freezing is opposite of what has been reported previously and thus also opposite from what we had hypothesized. (…)

Methods

Line 268. A brief statement of how freezing of gait was defined would be useful rather than just referring the reader to the Gilat reference. What happened after a freezing episode? Did the participants continue the trial?

We have added the definition of Nutt et al. and some clarification from the paper of Gilat that was used to define a freezing episode:

Line 273-276: We defined freezing as "a brief, episodic absence or marked reduction of forward progression of the feet despite the intention to walk".[4] The end of an episode was marked as the last toe-off after which the participant was able to perform at least two effective alternating steps.[68]

Participants were instructed to continue the trial after encountering a freezing episode. We have added this earlier on in the Method section ("Gait task"):

Line 176-178: If the participants experienced a freezing episode, they were instructed to continue the task to the best of their ability.

Line 276. Did some participants lack control data for the freezing-provoking tasks because of consistently freezing during these tasks? If so, how many?

Yes, for the turning task 7 participants showed freezing at each turn, and 6 participants did not show any freezing during turning. For the doorway task, 1 participant always showed freezing and 14 participants never showed freezing at the doorway. We have accounted for this imbalance in the data by applying hierarchical models as is stated at **line 295-298**:

Note that each PD participant contributed a various number of freezing events and successful gait events, yielding an imbalanced data design. Hierarchical models account for imbalanced data by *shrinkage*, meaning that the data from participants contributing less or more variable data are pulled towards the group mean.[71,72]

We additionally added information about the number of participants included in each of the statistical analyses in the figure legends (see for example figure 3)

Line 297. How were these regions of interest computed? Average of the channels as per supplementary Table 3?

We averaged the channels belonging to the same region of interest as displayed in the layout of Figure 2. This layout was designed a priori to cover the given regions of interest as good as possible and to contain a similar number of channels in each region (to keep the statistics between regions comparable). We used the calculated channel positions of Supplementary Table 3 to check whether the channels indeed corresponded to the intended regions. This corresponded reasonably well. We explain this procedure in the following lines:

Line 310-312: …we averaged the HbO values of the channels belonging to the same ROI corresponding to the layout of Fig. 2.

Line 195: The cap was designed to optimally cover the areas previously associated with freezing[16,43-47]
Line 200-204: We calculated the Montreal Neurological Institute (MNI) coordinates of each channel and estimated their underlying brain regions defined by automated anatomical labelling to check whether the channels indeed corresponded to the intended regions of interest[51] and to allow for a comparison to previous studies[16,34,46-49] (see supplementary material).

Did the authors look at hemispheric differences?
We only looked at hemispheric difference for turning as we did not have a priori hypotheses about the hemispheres for the other tasks. Because there were no significant hemispheric effects for turning, we pooled the data. We explain this in the following line:
Line 318-319: Left and right turns were pooled as they did not display significant direction effects, nor interaction effects with the hemisphere.

Results
Table 1.
I am surprised to see that there was no statistically significant difference in the Trails-making tests between the PD and Healthy control groups.
We were also surprised by this result and have verified that this result is indeed correct. Although the PD group overall showed higher processing times for the Trail-making test, this difference was not significant.

What does the % time frozen refers to?
This refers to the % time spent with freezing relative to the total time of the gait task (Gilat, 2019) and is considered a better measure for freezing severity than number of freezing episodes during a task. We have added this information in Table 1 and on line 337.
Line 337-338: The % time frozen (i.e., the % time spent with freezing relative to the total time of the gait task) and MDS-UPDRS scores were centered and standardized before model fitting.

Reviewer: 2

Comments to the Author
Dear authors!
Your fNIRS data of 23 PD patients and 22HCs showing that – cortical activity during stopping involving activation of the SMA and prefrontal cortex (PFC), and activity in the PFC during actual freezing events was lower than during voluntary stopping. During these freezing-provoking tasks, the freezers displayed higher activity in the premotor areas than controls. So these findings merit publication.
I have a small question about this article.
Why not differentiate between OFF or ON state freezing as an inclusion criterium? As u mentioned, the participants with PD performed all procedures in the OFF state. Will the outcome be different if the patients are OFF or ON -freezing patients? Considering the limited number of patients, a further analysis of specific freezing type is needed to get unbiased outcomes.
We thank the reviewer for their critical feedback. The reason we did not differentiate between ON and OFF state freezing as an official inclusion criterium as this would have required a formal

evaluation of freezing frequency ON and OFF medication. Given that we only tested the freezers OFF medication, we were not able to formally differentiate between these types of freezing. However, based on informal information from our participants, probably none of them displayed ON state freezing, so we focused on the results of this study in the light of OFF state freezing. We have clarified this in the manuscript on **line 123-126**:

> We did not differentiate between OFF or ON state freezing as an inclusion criterium as we did not formally evaluate freezing frequency in the OFF *and* ON state. However, almost all participants declared to have more or the same amount of freezing OFF medication.

Comments from the Scientific Editorial team:

-Please add an Abbreviated Summary (up to 50 words) of your paper that captures the main purpose and conclusions of the work. The summary should state the main results written in the third person ('[1st author surname] et al. report that …') and conclusion. This summary will be used in the contents list online and should not contain any abbreviations other than those on the accepted abbreviations list and accepted gene/protein names, making it accessible to the non-specialist. Please upload it as a Word document. Allowed abbreviations: AIDS; ANOVA; AMPA; ATP; A,T,C,G; CNS; CSF; CT; DNA; ECG; EEG; EMG; GABA; HIV; MRI; NMDA; PET; PCR; RNA.
Cockx et al. investigated freezing-related cortical activity during free ambulation in people with Parkinson's disease using functional near-infrared spectroscopy. Their results suggest that freezing results from an oversensitivity to stopping signals and an imbalanced activity between the supplementary motor area and the prefrontal cortex within the stopping network.

-Please provide a graphical abstract. A graphical abstract is a visual representation of the central finding or methodology of the paper. It will be displayed in the table of contents. Authors should strive to make the graphical abstract informative, interesting, visually appealing, and straightforward (no caption needed, no abbreviations except gene names). The technical requirements for the graphical abstracts are:
- Size: 120 x 120 mm (300-600dpi) or 3000 x 3000 pixels
- Font: Arial 12-16 pt
- File type: tiff or PDF
- Aspect ratio: 1:1 preferred

We have added a graphical abstract to our resubmission.

-Figure 2A, Supplementary figures 3, 7: Please revisit your colour choice in this figure as the green-red combination is not colour-blind friendly.
We have adjusted figure 2A and Supplementary Figure 7 so they are now colour-blind friendly. We did not adjust Supplementary Figure 3 as here the colors are of minor importance for this figure and only represent the participant ID's that are also displayed on the vertical axis.

-Figures 3, 5 and Box 1, Supplementary figures 4, 5: For each statistical analysis part of a figure, please report in the figure legend the statistical test used. Please give details of the sample size (N=) used in the statistical test, and if the number is <10 show the single data-points. Please note: We do not accept analyses where individual measurements are the experimental unit when there is likely to be biological variability between animals, culture replicates, etc. For this type of data,

we require either that a mean or median is taken per animal/culture replicate followed by statistical analyses, or that more sophisticated analyses are performed such as mixed effects modelling which can account for random effects.
We have added this information for each figure. No statistical testing was performed in Box 1, so no text was added here.

-Figure 4: For each statistical analysis part of a figure, please report in the figure legend the statistical test used.
We have added this information.

-Supplementary figure 1: For each statistical analysis part of a figure, please report in the figure legend the statistical test used. Please give details of the sample size (N=) used in the statistical test, and relevant test statistic values alongside p values (for example F or t values for ANOVAs and t-tests).
We have added this information.

-Supplementary figure 2: Please provide a figure legend so that the figure may be easily interpreted as stand-alone figures.
We have added this information.

-Supplementary figures 3, 6: For each statistical analysis part of a figure, please give details of the sample size (N=) used.
No statistical analysis was performed in these figures, so no extra information was added her.

-At the revision stage, if you plan to edit the current figures and tables, or to make new figures and tables, please make sure that these items fit a typeset journal page (A4).
